# Perceived legitimacy of layperson and expert content moderators

**DOI:** 10.1093/pnasnexus/pgaf111

**Published:** 2025-05-20

**Authors:** Cameron Martel, Adam J Berinsky, David G Rand, Amy X Zhang, Paul Resnick

**Affiliations:** Sloan School of Management, Massachusetts Institute of Technology, Cambridge, MA 02142, USA; Department of Political Science, Massachusetts Institute of Technology, Cambridge, MA 02139, USA; Sloan School of Management, Massachusetts Institute of Technology, Cambridge, MA 02142, USA; Institute for Data, Systems, and Society, Massachusetts Institute of Technology, Cambridge, MA 02142, USA; Department of Brain and Cognitive Sciences, Massachusetts Institute of Technology, Cambridge, MA 02139, USA; Allen School of Computer Science and Engineering, University of Washington, Seattle, WA 98195, USA; School of Information, University of Michigan, Ann Arbor, MI 48109, USA

**Keywords:** content moderation, misinformation, legitimacy, social media, partisanship

## Abstract

Content moderation is a critical aspect of platform governance on social media and of particular relevance to addressing the belief in and spread of misinformation. However, current content moderation practices have been criticized as unjust. This raises an important question—who do Americans want deciding whether online content is harmfully misleading? We conducted a nationally representative survey experiment (*n* = 3,000) in which US participants evaluated the legitimacy of hypothetical content moderation juries tasked with evaluating whether online content was harmfully misleading. These moderation juries varied on whether they were described as consisting of experts (e.g. domain experts), laypeople (e.g. social media users), or nonjuries (e.g. computer algorithm). We also randomized features of jury composition (size and necessary qualifications) and whether juries engaged in discussion during content evaluation. Overall, participants evaluated expert juries as more legitimate than layperson juries or a computer algorithm. However, modifying layperson jury features helped increase legitimacy perceptions—nationally representative or politically balanced composition enhanced legitimacy, as did increased size, individual juror knowledge qualifications, and enabling juror discussion. Maximally legitimate layperson juries were comparably legitimate with expert panels. Republicans perceived experts as less legitimate compared with Democrats, but still more legitimate than baseline layperson juries. Conversely, larger lay juries with news knowledge qualifications who engaged in discussion were perceived as more legitimate across the political spectrum. Our findings shed light on the foundations of institutional legitimacy in content moderation and have implications for the design of online moderation systems.

**Significance Statement**

Social media platforms have been criticized for their content moderation, particularly concerning online misinformation. Some have argued the platforms take too little action, while others have decried tech companies as political censors. Yet little work has examined who the public wants making these moderation decisions—which is integral for developing broadly accepted policies. We show US participants a series of content moderation juries—some consisting of experts and some consisting of laypeople. We find that people prefer expert to layperson juries—but that increasing layperson jury size, qualifications, and allowing juror discussion each help close this legitimacy gap. Our results shed light on what characteristics increase legitimacy and may help inform the design of both expert and layperson moderation systems.

## Introduction

Social media companies have come under increasing scrutiny for how they moderate content on their platforms. This question of platform governance is particularly relevant when it comes to addressing the belief in and spread of misinformation and harmfully misleading online content (1, 2). Some have criticized large tech companies like Facebook for not doing enough to reduce the dissemination of misleading content, particularly during the COVID-19 pandemic (3, 4) and in the wake of the 2016 and 2020 US presidential elections (5, 6). Conversely, social media platforms have also been accused of political censorship, particularly by those on the political right (7). For instance, Republican lawmakers in Florida and Texas have attempted to pass laws prohibiting tech companies from moderating posts from political candidates (8), Meta has recently announced an end to its partnership with third-party fact-checkers in the United States owing to what its chief executive Mark Zuckerberg has called “too much censorship” (9), and Donald Trump has even issued an executive order titled “Restoring Freedom of Speech and Ending Federal Censorship” aimed at making it more difficult for social media platforms to moderate digital content (10). However, underlying these disagreements and policy developments is an important question—who do social media users actually want making content moderation decisions of potential misinformation?

Understanding what content moderation decision-makers and processes the public find legitimate is an important prerequisite for building robust and effective mechanisms for addressing instances of harmfully misleading content online. The moderation of social media platforms constitutes a new form of news and expression governance (11). Thus, just as it is valuable to understand perceptions of procedural fairness and their impact on the legitimacy of laws and courts (12, 13), so too is it valuable to examine who Americans perceive as legitimate adjudicators of what constitutes harmfully misleading content on social media. As a result, social media platform policies have increasingly been analyzed through a lens of Internet governance (14), and empirical work has examined user preferences for content moderation of potentially harmful falsehoods, such as hate speech (15) and false information (16). And while the policy decisions of social media platforms, unlike public institutions, are ultimately under the purview of private companies, these platforms still rely on active usership and can be responsive towards the expressed perceptions and preferences of their user base. Thus, herein we investigate what types of content moderators and moderation procedures the public perceives as legitimate for social media platforms to use.

One approach many major tech companies have at various times used for handling on-platform misinformation is partnership with professional third-party fact-checking organizations. This system involves platforms sending content to professional fact-checkers that may be false or misleading—then posts evaluated as such by the fact-checkers may be labeled, downranked, or removed depending on the platform's policies regarding action on such content. Until recently, Facebook and Instagram partnered with fact-checkers to identify false content for labeling and demotion (17, 18); TikTok currently partners with fact-checkers and purports to remove entirely some false content (19). Interventions such as warning labels and label-based downranking have been shown to effectively reduce engagement with and consequences of identified posts (20, 21). However, relying only on professional fact-checkers has several limitations. The number and growth of fact-checking organizations is limited (22), which limits the ability of fact-checkers to keep pace with the scale of on-platform misinformation. Expert moderation is also dependent on which types of experts platforms choose to partner with and fund—for instance, US-based platforms may have greater ties with English-language experts, resulting in inequitable policy enforcement for non-English misinformation (23). Additionally, expert moderation has come under extreme scrutiny from those on the political right—resulting in major platforms like Facebook and Instagram ending their partnerships with third-party fact-checkers in the United States (9).

As a result, several social media platforms also employ various nonexpert moderation approaches. One is using crowdsourced assessments from platform users. Recent work suggests that layperson crowds can help identify misinformation (24–26). Several tech platforms have employed various forms of crowd moderation. X (formerly Twitter) operates Community Notes—a feature whereby users can write free-response notes that provide additional context or counter information about tweets, and other users vote on whether the notes should be attached to the tweets for all other users to see (27–29). Meta has also incorporated community content reviews whereby nonexperts are recruited and paid to fact-check content (30), and, more recently, has announced a plan to transition content moderation from partnership with third-party fact-checkers to developing a Community Notes model of their own (31). YouTube has also recently begun development of a crowdsourced fact-checking initiative (32).

Another method used by social media platforms to identify misinformation at scale is the use of algorithmic identification. Over the years, researchers have innovated and tested various misinformation classifiers (33), including most recently some promising evidence as to the ability of large-language models such as GPT to fact-check the news (34, 35). In practice, most major tech platforms have proprietary algorithms that they use to identify potentially misleading claims. For instance, Facebook uses AI tools to detect harmful content and to perform image- and claim-matching across posts containing related content (36). Algorithms designed to identify misleading content also help inform which posts are sent to community reviewers and professional fact-checkers for review (30).

However, each of these approaches—experts, laypeople, and algorithmic identification—has come under criticism. There are high levels of distrust in fact-checkers and journalists, particularly among Republicans and those on the political right (37–39). Such trends are consistent with declining confidence and trust in American institutions more generally (40, 41), and are particularly troubling for tech platforms that may be seen as disproportionately censoring politically discordant viewpoints (7, 42). Digital juries of laypeople may have more potential to be trusted, particularly on the political right, given their conception as a civics-oriented method of democratizing the content moderation process (43). However, some preliminary evidence suggests that experts may still be more effective or more legitimate content moderators. For instance, warning labels are more effective when attributed to fact-checkers than layperson crowds (44). Algorithmic content moderation also suffers from low perceived confidence—labels attributed to AI are even less effective than those from the public (44), and visible AI moderator systems are subject to less perceived trust and accountability in the moderation process (45). Furthermore, a recent experiment of 100 Facebook users found that moderation decisions made by expert panels were perceived as more legitimate than decisions from layperson juries or algorithms (46).

Together, this literature suggests that despite distrust of experts, layperson crowds are not automatically a more institutionally legitimate alternative. This poses an important question—are there specific types of crowds that may be perceived as more legitimate relative to experts? For instance, layperson crowds have the flexibility to consist of different possible configurations, such as politically balanced (47) or nationally representative (48, 49) samples. Such compositions may be preferable given concerns of political bias or inequity in existing moderation systems. Existing work has also demonstrated that crowds selecting for individuals higher in political knowledge achieve better performance (24, 50)—suggesting that enhancing perceived layperson competence may promote both the perceived and actual evaluations of layperson crowds. In addition to varying the composition of moderators, different approaches may also vary important procedural aspects—such as whether moderators make decisions independently or whether they may deliberate with one another (51, 52). Understanding the compositional and procedural features that improve crowdsourced moderation legitimacy is particularly important given the intention of social media platforms like X, Facebook, Instagram, and YouTube to continue developing and relying on Community Notes style initiatives for sustaining content moderation efforts (27, 31, 32).

Our current work investigates Americans’ content moderation perceptions and preferences. We examine how individuals perceive various expert and layperson digital juries (43) tasked with deciding what social media content is harmfully misleading. Specifically, we evaluate perceptions of content moderation institutional legitimacy in the crucial cases where individuals disagree with the moderation decision made. We also shed light on specific compositional and procedural features that increase legitimacy perceptions of moderation panels. Our findings provide insight into when and how both expert and layperson panels may be best employed for investigating potentially misleading online content. We also examine partisan differences in such preferences. Prior work has found greater unwillingness among Republicans than Democrats or independents to endorse removal of misinformation or penalization of accounts spreading misleading content (16, 53), as well as greater distrust among those on the political right of fact-checkers, journalists (37–39), and even domain experts like scientists (54). Here, we assess whether there are partisan differences in the perceived institutional legitimacy of expert and layperson content moderation compositions and procedures.

We conducted a nationally representative quota-matched survey of 3,000 US respondents on YouGov between 2003 July 17 and 2023 August 23. This sample size was predetermined by our budget constraints and preregistered accordingly (https://aspredicted.org/M1G_FXT). The main survey task was a rating- and choice-based conjoint style experiment in which we varied features of content moderation juries tasked with deciding whether pieces of online content should be labeled “harmfully misleading” on a social media platform such as Facebook. Participants were told that each jury would be provided with background information on the content to be evaluated and the ability to search for more information (25).

Importantly, each participant was asked to assess the legitimacy of decisions under the precondition that the participant *disagreed* with a decision that the jury made. Prior work on institutional legitimacy argues that considerations of legitimacy are most important when there is an “objection precondition”—that is, disagreement with an institution's decision may trigger disappointment or disapproval, which in turn could result in individuals questioning whether said institution has the right to make the decision to begin with (55). Gibson even defines legitimate institutions as “those recognized as appropriate decision-making bodies even when one disagrees with their outputs” (55). And empirically, outcome alignment of moderation decisions is an important factor affecting perceived legitimacy (46). Thus, the real policy challenge is to devise a process that people accept as legitimate even when they disagree with the outcome (56). Therefore, we made the experimental design choice to focus on this decisive assessment in order to evaluate levels of perceived legitimacy of content moderation procedures even in the face of disagreement and to examine what characteristics and procedural features of content moderation can improve legitimacy under this critical test case.

For each jury profile, we randomized four characteristics: (i) who is evaluating the online content, (ii) the size of the jury, (iii) the qualifications of jury members, and (iv) if the jury discusses among themselves or not. We chose to vary these characteristics for several reasons. Theoretically, prior work has investigated the relationship between legitimacy and attributes such as expertise, jury size (57), and deliberation (58). Pragmatically, these features were chosen to reflect basic yet implementable features of actual potential content moderation panels—any actual content moderation panel would need to predetermine who and how many individuals are making the decision, whether there should be any qualification or vetting process, and whether decision-making should be independent or deliberative.

We randomized jury profiles to be one of nine possible compositions—three possible expert juries (professional fact-checkers, professional journalists, or domain experts [e.g. health professionals for medical information]), three possible layperson juries (jury randomly selected from a nationally representative pool, jury randomly selected from a pool of users of the social media platform, or a politically balanced jury), and three nonjuries which we expected to have lower baseline legitimacy evaluations (coin flip, head of the social media company [e.g. Mark Zuckerberg of Facebook], or a computer algorithm). All expert juries were fixed at a size of three members and with “professional qualifications.” Layperson juries were randomized as sizes of three members, 30 members, or 3,000 members. Layperson juries were also randomized as either having passed a test demonstrating a minimum level of news knowledge and reasoning ability, or as having no minimum qualifications. Both expert and layperson juries were randomized as either requiring that jurors evaluate content independently (without discussion) or that jurors discuss content with each other during the evaluation process. For nonjuries, size, qualifications, and discussions were all fixed as “not applicable.”

Each participant evaluated 20 unique juries in 10 total pairs. Juries were randomized such that each participant viewed six expert juries, 12 layperson juries, and two nonjuries. For each jury pair, a participant first evaluated them one at a time, viewing a grid summary of a single jury's characteristics and answering five 7-point Likert-scale questions on their perceptions of the jury's legitimacy (adapted from (46); e.g. “Jury 1 should be the authority making moderation decisions”; see “Legitimacy ratings” section in Methods). Concretely, we conceptualize and operationalize legitimacy through a measure of institutional legitimacy adapted from (46) and (12) in which these five questions each assess a separate component measure of perceived institutional legitimacy—outcome satisfaction, trustworthiness, fairness and impartiality, institutional commitment, and decisional jurisdiction (see Methods for measures of internal consistency between items).

**Fig. 1.**
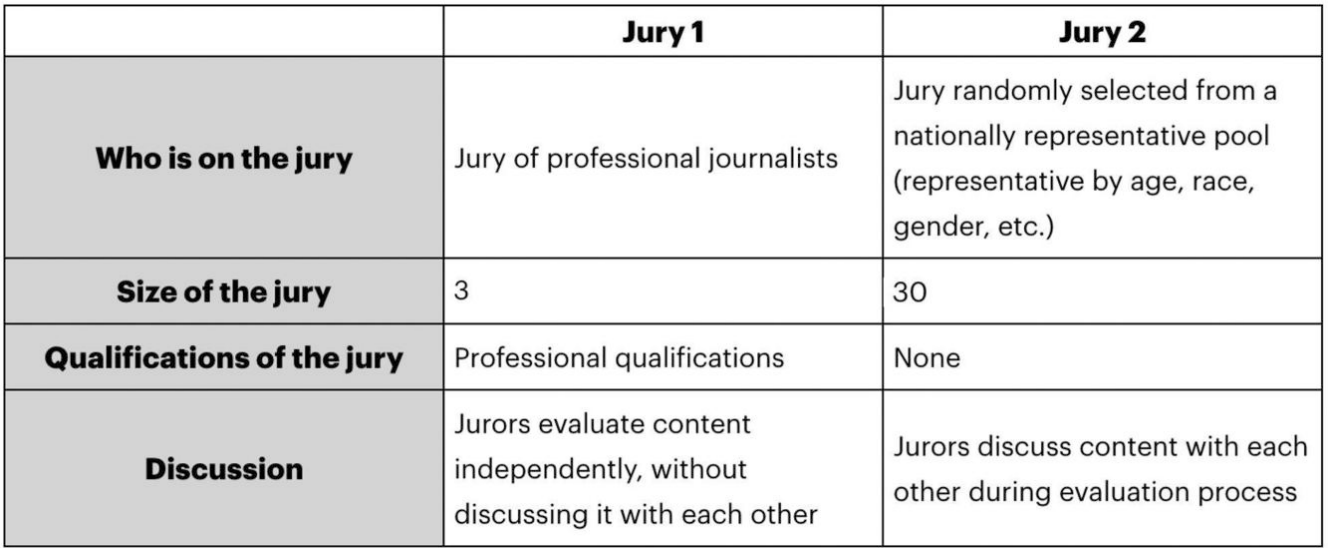
Example content moderation jury pair. Each participant viewed 10 pairs of content moderation juries. For each jury within a pair, participants first answered a five-item battery of perceived legitimacy questions. Then, as above, participants viewed both juries again and were asked which they would prefer to have evaluate online content.

Then, participants viewed a combined table showing both jury feature sets together (see Fig. 1) and were asked which jury they would prefer to have evaluating online content (see “Jury choice” section in Methods).

For each individual jury, we use linear regression to predict average perceived legitimacy rating. We use domain experts (without discussion) as our holdout jury type. Our predictors include dummy variables for whether the jury was a coin flip, head of the social media company (e.g. Mark Zuckerberg of Facebook), or a computer algorithm (our nonjuries); dummy variables for whether the jury was a nationally representative jury of laypeople, a politically balanced jury of laypeople, or a jury of users from the social media platform (our layperson juries); dummies for whether the jury was professional fact-checkers or professional journalists (our expert juries; excluding hold out of domain experts); all interactions between layperson dummies, size (30, 3,000), qualifications, and discussion; and all interactions between expert dummies and discussion; with cluster robust standard errors by participant (see Eq. 2 in Methods). We specify baseline jury features as a size of 3, no qualifications (for laypeople), and no discussion.

We also conduct similar analyses at the jury pair level, using a linear probability model to predict the choice of juries by these same predictors. Domain expert juries (without discussion) are again our holdout level.

Our experimental procedures and analyses were preregistered here: https://aspredicted.org/M1G_FXT. For more details, see Methods section. Our full survey codebook, data, and analysis code are also available on our OSF page here: https://osf.io/7tjhb/.

As preregistered, we also conduct nearly identical analyses, as described above, except collapsing our nine jury compositions into three groups: nonjuries, laypeople, and experts. These results are reported in full in SI Section S4. We also conduct robustness checks for all analyses as follows: (i) filtering for highly attentive participants (59) and (ii) accounting for intrarespondent reliability as assessed via a repeat conjoint item we included at the end of our main task (60). All robustness analyses and results are included in our Supplementary material (see SI Sections S6–S8)—our results are qualitatively similar across all robustness assessments. We also reconduct all our analyses with analytic weighting by YouGov calculated US population weights—all results are nearly identical to our sample results reported in the main text (see Supplementary material for population-weighted results).

## Results

**Fig. 2.**
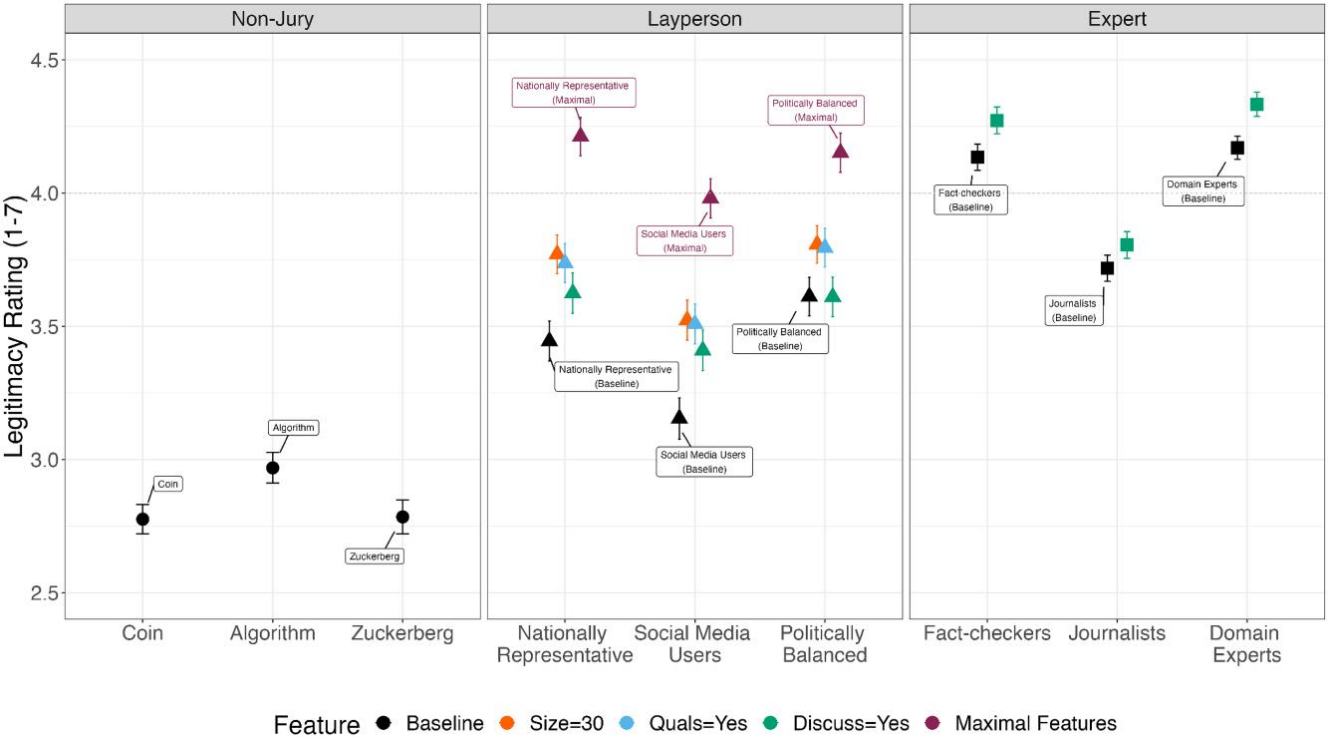
Jury-level legitimacy ratings. The average legitimacy ratings for all nine juries, with different size, qualification, and discussion features. Estimates reflect sample-level averages. Error bars reflect 95% CIs. Absolute legitimacy ratings should be interpreted as those under our objection precondition wherein participants were told to suppose they disagreed with a decision from the content moderation jury. See Fig. S5 for the corresponding choice outcome variable plot. See Figs. S2 and S3 for results collapsed across jury category type (nonjury, layperson, and expert).

In our model, domain experts had nominally the greatest baseline perceived legitimacy (note that absolute legitimacy ratings should be interpreted given our objection precondition design; intercept = 4.170, SE = 0.022, *t* = 186.895, *P* < 0.001; see Fig. 2; Table S2[1]), marginally greater than that of professional fact-checkers (*b* = −0.035, SE = 0.020, *t* = −1.766, *P* = 0.078) and greater than our third expert option of journalists (*b* = −0.452, SE = 0.021, *t* = −21.113, *P* < 0.001). As expected, all three nonjuries exhibited considerably lower perceived legitimacy: computer algorithm (*b* = −1.201, SE = 0.033, *t* = −36.775, *P* < 0.001), a coin flip (*b* = −1.393, SE = 0.034, *t* = −41.359, *P* < 0.001), and the head of the social media company (*b* = −1.385, SE = 0.035, *t* = −39.276, *P* < 0.001).

Baseline layperson juries were evaluated as having legitimacy ratings between that of experts and nonjuries. Relative to domain experts, social media users (*b* = −1.016, SE = 0.042, *t* = −24.062, *P* < 0.001), a nationally representative panel (*b* = −0.725, SE = 0.040, *t* = −18.060, *P* < 0.001), and a politically balanced panel (*b* = −0.558, SE = 0.038, *t* = −14.615, *P* < 0.001) were each perceived as significantly less legitimate under baseline conditions.

**Fig. 3.**
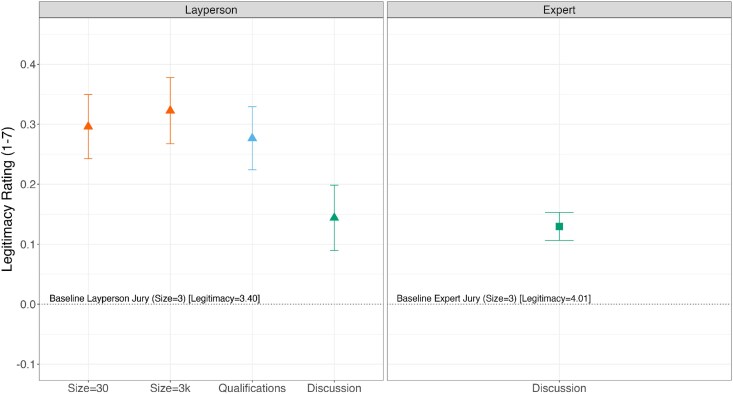
Category-level effects of jury features on perceived legitimacy. Left: Relative to the baseline layperson jury with a size of three members (average legitimacy = 3.40), the effects of individually increasing jury size to 30, increasing jury size to 3,000, adding minimum qualifications, or allowing discussion among jury members. Right: Relative to the baseline expert jury (average legitimacy = 4.01), the effect of allowing discussion among jury members. Estimates reflect sample-level effects. Error bars reflect 95% CIs. See Fig. S4 for the corresponding choice outcome variable plot.

However, additional jury composition features helped close the legitimacy gap. All three layperson jury types exhibited significantly increased legitimacy perceptions when jury size was increased from 3 to 30 or 3,000 (*P* < 0.001; though notably, there was no additional benefit of an increase to 3,000). All three layperson jury types also exhibited significantly increased legitimacy when minimal knowledge qualifications were instituted (*P* < 0.001). Figure 3 shows the effects of varying these attributes as estimated from our model collapsing across jury types—size and qualifications increase the perceived legitimacy of layperson juries.

Allowing jury discussion also increased legitimacy perceptions for most jury compositions. This was the case for domain experts (*b* = 0.163, SE = 0.017, *t* = 9.463, *P* < 0.001), and we did not find a significantly different discussion effect on most other expert and layperson jury types (see Fig. 3 for discussion effects on collapsed layperson and expert panels). Two exceptions were journalists (interaction: *b* = −0.075, SE = 0.022, *t* = −3.345, *P* < 0.001) and politically balanced layperson juries (interaction: *b* = −0.164, SE = 0.051, *t* = −3.196, *P* = 0.002), for which the discussion effect was significantly smaller than that for domain experts.

The combined effects of multiple features for layperson juries were largely additive—such that nationally representative and politically balanced juries with maximal features (size of 30, qualifications, discussion) exhibited comparable legitimacy (maximal nationally representative jury mean legitimacy = 4.212, 95% CI = [4.140–4.284]; maximal politically balanced jury mean legitimacy = 4.151, 95% CI = [4.078–4.225]) to baseline domain experts (no discussion; baseline domain expert jury mean legitimacy = 4.170, 95% CI = [4.126–4.213]; *z*-test of coefficients: *z* = 0.986, *P* = 0.324; *z* = −0.415, *P* = 0.678, respectively).

At the jury pair level, we observed qualitatively similar results (see Table S2[3]). Here, we found that maximal nationally representative (mean choice probability= 0.676, 95% CI = [0.647–0.705]) and politically balanced lay juries (mean choice probability = 0.670, 95% CI = [0.641–0.700]) matched the choice probability in our paired design of domain experts with discussion (mean choice probability = 0.661, 95% CI = [0.644–0.678]), the highest performing expert panel (*z*-test of coefficients: *z* = 0.882, *P* = 0.378; *z* = 0.569, *P* = 0.570, respectively; see Fig. S5).

### Results by partisanship

Given prior work indicating that US Republicans have greater distrust of experts like fact-checkers (37–39) and that even crowd ratings have been shown to be less effective for those on the political right in some instances (61), we also investigated how partisanship may moderate our findings. To do so, we conducted all aforementioned analyses, except adding partisanship (hold out = Republican, Independent, Democrat; classified using 7-point partisanship scale, with Independents as midpoint responders) and allowing for all interactions.

**Fig. 4.**
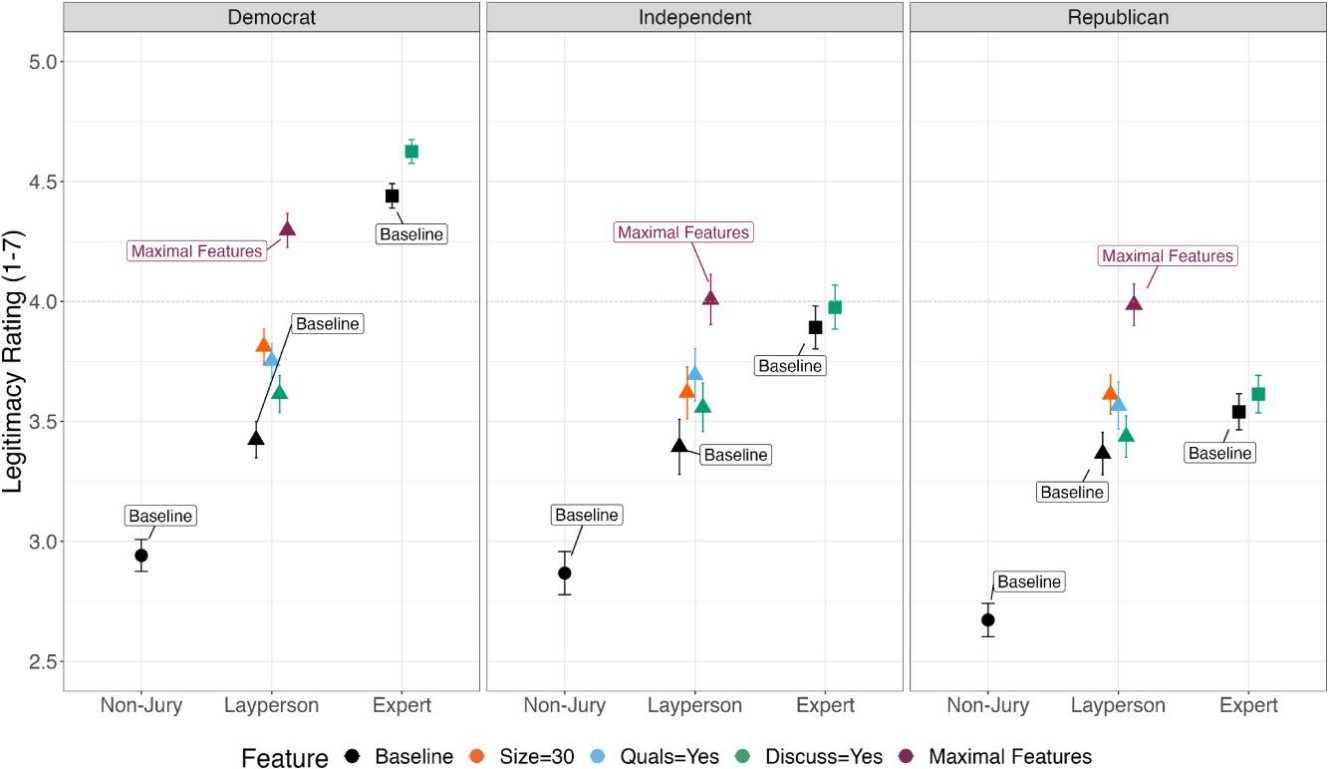
Legitimacy ratings by partisanship. Collapsed by category: nonjury, layperson, expert. Estimates reflect sample-level averages (see supplementary tables for population-weighted estimates). Error bars reflect 95% CIs. Absolute legitimacy ratings should be interpreted as those under our objection precondition wherein participants were told to suppose they disagreed with a decision from the content moderation jury. See Fig. S6a for the corresponding choice outcome variable plot. See Fig. S7 for results disaggregated by all nine juries.

For simplicity, we first assessed our analyses collapsing within nonjury, layperson, and expert jury types. Republicans evaluated baseline expert juries as more legitimate than both layperson juries and nonjuries (*P* < 0.001; see Fig. 4; for full statistics, see Table S3[1])—though Democrats and Independents each perceived experts as more legitimate than did Republicans (*P* < 0.001). Furthermore, Democrats and Independents perceived a greater legitimacy gap between baseline experts and laypeople compared to Republicans (*P* < 0.001)—though nonjury and layperson legitimacy perceptions were relatively similar for all partisans. Increased size, qualifications, and allowing discussion each further increased the legitimacy of layperson juries for Republicans (*P* < 0.001). This was largely the same across the political spectrum—though increasing layperson jury size to 30 and allowing for expert discussion had larger effects on increasing legitimacy for Democrats relative to Republicans (interaction term *P* < 0.027). For Republicans, layperson juries with maximal features were perceived as more legitimate than expert juries with discussion (*z*-test of coefficients: *z* = 6.26, *P* < 0.001). The converse was true for Democrats, who still preferred the best expert configuration to the best laypeople configuration (*z*-test of coefficients: *z* = −7,44, *P* < 0.001). Independents perceived maximal layperson (mean legitimacy = 4.008, 95% CI = [3.903–4.113]) and expert juries (mean legitimacy = 3.976, 95% CI = [3.884–4.068]) as similarly legitimate (*z*-test of coefficients: *z* = 0.454, *P* = 0.650). All results were qualitatively similar when examining jury choice in our paired design (Table S3[3]).

We further reexamined our analyses assessing all nine possible jury types, including partisanship as a moderator. Republicans evaluated domain experts as the most legitimate baseline jury composition, though less so compared with Democrats’ (*P* < 0.001) and Independents’ (*P* = 0.006) evaluations of domain experts (see Fig. S7; for full statistics, see Table S4[1]). This legitimacy gap was even greater between Republicans and Democrats for fact-checkers (*P* < 0.001), such that Republicans generally perceived all expert panels as less legitimate than Democrats did, despite domain experts still being the most favorably evaluated baseline jury by all partisans. For Republicans, increasing jury size and adding minimum knowledge qualifications increased legitimacy perceptions for all three layperson jury types (*P* < 0.014)—with one exception being that we did not find evidence that qualifications increase the legitimacy of politically balanced juries (*P* = 0.685). Size and knowledge qualification effects on layperson juries were relatively similar for Democrats and Independents. For Republicans, discussion increased the perceived legitimacy of domain experts (*P* < 0.001) and similarly so for fact-checkers and all three layperson jury types (*P* > 0.390). Interestingly, the benefit of discussion among journalists was significantly less than that for domain experts (*P* = 0.029). Overall, evaluations of nonjuries and layperson juries were similar for Republicans, Democrats, and Independents, and additional qualifications had similar effects across the political spectrum. The main cross-partisan difference was lower perceived legitimacy of experts by Republicans—though domain experts and fact-checkers were still the two most legitimate baseline juries even for those on the political right. All results were similar when examining jury choice as our dependent variable (Table S4[3]).

## Discussion

It is critical for social media platforms to deploy content moderation systems capable of effectively addressing issues such as the spread of harmfully misleading content (62, 63). In order to bolster public trust in such initiatives, these moderation systems should be perceived as institutionally legitimate, even in the face of disagreement. However, prior work has brought into question public trust in experts, crowds, and other systems by which platforms may evaluate potentially misleading content. Our results indicate that despite concerns of distrust in expertise, content moderation panels consisting of experts such as domain specialists, fact-checkers, and—to a lesser extent—journalists are generally perceived as more legitimate decision-makers than layperson juries, and both are perceived as more legitimate than algorithms. However, modifying compositional and procedural aspects of moderation juries can bolster legitimacy evaluations. Increasing the size of a lay jury, requiring minimum knowledge or reasoning qualifications, and allowing for discussion among jurors each increase perceived jury legitimacy and the probability that individuals would prefer a given moderation panel in pairwise comparisons. Furthermore, these effects are additive, such that some layperson juries with increased size, qualifications, and discussion can equal, or even surpass, the legitimacy and preferability of expert juries.

We also examined whether such effects were observed across the political spectrum. Republicans perceived expert panels as less legitimate relative to Democrats but still evaluated experts as more legitimate than laypeople at baseline. In turn, jury features such as size, qualifications, and discussion more readily closed the legitimacy gap between laypeople and experts for Republicans than Democrats. Republicans and Democrats perceived layperson juries as comparably legitimate, suggesting that partisan asymmetries in content moderation legitimacy perceptions are largely instantiated in expert evaluations. These results also suggest that further interventions to promote the perceived legitimacy of various types of moderators should consider the partisanship of the target audience—promoting expertise for Republicans may help raise the absolute level of legitimacy for experts, while promoting laypeople crowds for Democrats could help further close the relative legitimacy gap between expert and layperson panels.

Our results have several interesting theoretical implications. First, although Republicans perceive domain experts as the most legitimate baseline jury, their evaluations are considerably lower than that of Democrats—consistent with literature on anti-intellectualism (64, 65) and high levels of distrust in media professionals and scientific experts, particularly among Republicans (37–39, 54, 66, 67). However, we still observe a significant and consistent effect of expertise among layperson juries via requiring minimum knowledge qualifications for jurors. This contradicts the notion that Republicans do not find characteristics such as news knowledge or reasoning ability as beneficial for legitimacy. Rather, these findings suggest that Republicans find experts as less legitimate moderators for reasons other than their qualifications and expertise per se. Lower legitimacy of experts may be because of who the experts are rather than what they know—echoing concerns of political bias among moderators and fact-checkers (7, 37). Republicans and Democrats alike seem to value expertise independently—but differences arise in the evaluations of particular classes of experts, indicating that individuals may have dissociable evaluations of expert skill and bias (68).

Second, our findings provide some evidence as to how the public perceives features prevalent in research on the “wisdom of crowds.” Increasing the size of layperson juries from 3 to 30 members reliably enhanced perceptions of legitimacy—consistent with theoretical and empirical data that bigger groups produce more accurate crowd estimates (69). However, a further increase from 30 to 3,000 members produced no significant additional benefit (see Fig. 3), suggesting declines in the marginal impact of further increasing group size in this content moderation jury setting. We also find that for most expert and layperson jury types, allowing for discussion among jurors increases the perceived legitimacy of the jury. While jury deliberation is consistent with US legal jury practices (51) and theories of deliberative democracy (52), empirical evidence is mixed and context specific as to whether deliberation among group members improves crowd judgments. In some cases, independent judgments can benefit aggregate assessments by reducing the influence of poorer performing group members (70)—though in other cases, group collaboration or being able to see the assessments of other evaluators can improve overall judgments (71). Whether discussion and deliberation actually improve crowd wisdom depends on various group and task parameters (72). Interestingly, Americans evidently believe that content moderation juries are a setting where discussion should help, rather than hinder, decision-making.

Our results also have practical implications for social media platforms and content moderation system regulators. Despite concerns and a partisan gap between Democrats and Republicans, experts, such as domain specialists and fact-checkers, are perceived as the most legitimate content moderators, holding other jury characteristics constant. Our results suggest that platforms should continue to utilize media professionals and other experts for content moderation decisions, and draw into question whether recent policy changes, such as Meta's decision to end its partnership with professional fact-checkers (9), actually reflect the preferences of even its right-leaning users.

That said, experts are still limited in number and the breadth of content coverage they may provide. Thus, it is important to also understand whether alternative moderation systems may also be perceived as legitimate. When they are representative or politically balanced, are bigger, have individual knowledge qualifications, and are allowed to discuss, lay juries may indeed match the perceived legitimacy of expert juries. These results suggest that both expert and layperson moderation procedures can increase the institutional legitimacy of online moderation practices.

Both expert and layperson panels are perceived as more legitimate than a generic computer algorithm approach. In practice, expert and layperson content moderation juries may be used to either make decisions about potentially misleading content or potentially help inform the standards by which algorithmic identification should be trained on and evaluated. The latter in particular may help translate the legitimacy benefits of experts or well-qualified layperson panels to more scalable algorithmic approaches and help quell future debates about the over- or under-reach of moderation of misleading content. Recent advancements in large-language models and generative AI may also have notable effects on perceptions of algorithmic content moderation. For instance, participants who engaged in a conspiracy theory debunking conversation with GPT-4 exhibited a large increase in trust in AI (73)—providing some evidence that engaging with automated moderation efforts may increase endorsement of algorithmic moderation—provided such moderation is effective and accurate.

The current research has several limitations to note. First, in our legitimacy rating analyses, we find relatively low absolute levels of legitimacy perception. For Democrats, the only layperson juries that exceed the scale midpoint of legitimacy are those with maximal qualifications; for Republicans, only domain experts with discussion and the best-performing layperson juries even approximate the scale midpoint. Importantly, however, these absolute legitimacy magnitudes only reflect those under the objection precondition of our experimental design, in which participants were instructed to suppose that each panel had made a decision that they themselves disagreed with. Given that outcome alignment is a strong predictor of legitimacy perceptions following realized moderation decisions (46), it is to be expected that overall legitimacy evaluations are low given this paradigm. Polling on Americans’ support for content moderation of misinformation in general is typically quite favorable (74–76). In our paradigm, it may simply be much harder to have high absolute levels of acceptance of disagreeable outcomes as institutionally legitimate in the content moderation space. That said, even in this critical disagreement test case, we also do not observe very low absolute legitimacy ratings for most layperson and expert juries (typically within half a scale point of the scale midpoint). Further, our focus is primarily on the relative differences between content moderation juries when manipulating their compositional and procedural features. Through these analyses, we find that even under conditions of disagreement, these features matter—expertise, representativeness, and procedures like qualifications and discussion each improve legitimacy perceptions.

Second, and relatedly, all legitimacy evaluations were about content moderation panels without showing any actual decisions made by such entities—that is, we investigated the ex-ante legitimacy perceptions and preferability of hypothetical content moderation juries making decisions described as disagreeable in the abstract. One limitation on generalizability as a result is our current work only investigates absolute and relative perceptions of legitimacy under the precondition of decision disagreement. While this is a critical test case for assessing instances where legitimacy is most likely to be questioned (55, 56), it is also important for future work to investigate cases where moderators agree with participants—and how legitimacy evaluations across juries may interact with varying levels of agreement with jury decisions. A second limitation on generalizability is that we do not provide any concrete instances or examples of moderation decisions on specific pieces of online content. In our current work, we investigate the important first step of ex-ante legitimacy considerations without additional considerations of actual realized decisions. However, given the importance of realized outcome alignment on ex-post legitimacy and variation in moderation preferences by content type and severity (16, 46, 77, 78), future work should examine how legitimacy evaluations and actual interventions (e.g. warning labels or additional context) delivered by these moderation panels may vary when delivered via actual content decisions. Future research may also examine whether the implementation of legitimate expert or crowd panels positively affects moderation rule compliance and behavioral acceptance of the moderation process on-platform—for instance, as measured by recidivism of posting misleading content or via complaint rates of moderation decisions. In particular, it would be interesting to investigate how individuals update their legitimacy perceptions of content moderation panels over time given exposure to multiple moderation decisions varying on levels of agreement between the jury and focal user, as well as varying on decisions to moderate or to not moderate certain pieces of content (e.g. whether potentially misleading content should or should not receive a label).

Third, our findings are specific to Americans and the US context (and are robust to general population weighting)—future work may extend such questions to legitimacy perceptions cross-nationally, as has been done with assessing the efficacy of misinformation interventions and crowdsourcing (79, 80). Legitimacy perceptions and preferences may vary across different relevant groups within the US context—for instance, between the user bases of particular social media platforms. Investigating this possibility may be important for the development of platform-specific moderation strategies. Relatedly, we specifically told participants to think about moderation panels on a platform such as Facebook—but individuals may prefer different panels across different platforms (e.g. the more professionally oriented LinkedIn). Further research may investigate this possibility.

Our findings are also specific to the context of investigating the perceived legitimacy of policies hypothetically enacted by private tech companies. Much prior work on institutional legitimacy examines attitudes and perceptions on public institutions, such as courts (12). It is important, however, not to conflate the perceived legitimacy of private content moderation with that of public or governmental speech moderation—the latter of which we do not investigate here. Prior polling has measured support for both tech companies and the US government taking steps to restrict online false information (76). In our current work, we only probe perceptions towards policies that tech companies may enact to identify potentially harmfully misleading online content. These legitimacy perceptions likely differ from what policies individuals would find legitimate for the government to enact—but such perceptions of private moderation efforts are still important and impactful for ascertaining what types of polices are likely to be robust and broadly accepted on social media platforms. Future work may examine what types of juries or related procedures people find legitimate for public institutions to use when ascertaining whether content or statements may be false or misleading.

In sum, we show evidence from a nationally representative sample of Americans that the public perceives experts, such as domain specialists and fact-checkers as the most legitimate moderators of misleading online content—but that layperson juries can be increasingly and comparably preferable if they are representative or balanced, large, well-qualified, and engage in deliberative discussion. Our findings shed light on the foundations of legitimacy in content moderation and may help inform the combined usage of expert and layperson evaluators participating in legitimate platform governance.

## Methods

### Participants

As preregistered, we recruited a quota-matched, nationally representative sample of 3,000 US residents from YouGov between 2023 July 17 and 2023 August 23. Following our preregistration, this included only participants who passed two trivial attention checks at the beginning of the survey (e.g. captcha).

### Materials

For our main conjoint experiment task, participants were told the following: “We will show you several possible juries. Each jury has been asked to evaluate whether some piece of online content should be labeled ‘harmfully misleading’ on a social media platform such as Facebook. Suppose that all jury members are provided with some background information on the content they evaluate and an opportunity to search for more information.” Participants were next informed that: “Each jury profile will list (i) who is evaluating the online content, (ii) the size of the jury, (iii) the qualifications of jury members, and (iv) if the jury discusses among themselves or not.” They were also told to: “Suppose each jury made a decision about a particular piece of content—and you disagree with their decision.” Finally, participants were told: “For each jury, we will ask you a series of questions. We are generally interested in how legitimate you consider each jury, even if you disagree with a decision that jury made. Examine each jury profile carefully before answering the questions that follow.” Before starting the task, participants were asked to answer a free-response question about what characteristics, in general, they think would make juries tasked with determining whether online content is harmfully misleading reach more (or less) legitimate decisions.

Next, participants began the main conjoint task. Each participant was presented with 20 juries across 10 jury pairs. Each jury varied on who was on the jury, its size, requisite qualifications, and allowance of discussion. There were three possible nonjuries: computer algorithm, coin flip, and head of the social media company (e.g. Mark Zuckerberg of Facebook). Nonjuries were specified as “NA” for size, qualifications, and discussion. There were three possible layperson juries: a jury randomly selected from a pool of users of the social media platform, a jury randomly selected from a nationally representative pool (representative by age, race, gender, etc.), and a politically balanced jury. Layperson juries could also vary by size (3, 30, and 3,000), whether there were minimum qualifications required (“None,” “Passed a test demonstrating minimum level of news knowledge and reasoning ability”), and discussion (“Jurors evaluate content independently, without discussing it with each other,” “Jurors discuss content with each other during evaluation process”). There were three possible expert juries: a jury of professional journalists, a jury of professional fact-checkers, and a jury of domain experts (e.g. health professionals for medical information). Expert juries had a fixed size of three and fixed qualifications of “Professional qualifications,” though could vary in discussion allowance. For full conjoint jury possibilities and exact feature wordings, see Table 1.

**Table 1.** All possible jury features and conditional randomization procedure.

	Who is on the jury	Size of the jury	Qualifications of the jury	Discussion
Nonjury	[Coin flip] X [Computer algorithm] X [Head of the social media company (e.g. Mark Zuckerberg of Facebook)]	NA	NA	NA
Layperson	[Jury randomly selected from a nationally representative pool (representative by age, race, gender, etc.)] X [Jury randomly selected from a pool of users of the social media platform] X [Politically balanced jury]	[3] X [30] X [3,000]	[None] X [Passed a test demonstrating minimum level of news knowledge and reasoning ability]	[Jurors evaluate content independently, without discussing it with each other] X [Jurors discuss content with each other during evaluation process]
Expert	[Jury of professional fact-checkers] X [Jury of professional journalists] X [Jury of domain experts (e.g. health professionals for medical information)]	3	Professional qualifications	[Jurors evaluate content independently, without discussing it with each other] X [Jurors discuss content with each other during evaluation process]

Exact wordings of each feature are reproduced.

In sum, there were 45 total possible jury configurations (three nonjuries, six expert juries, and 36 layperson juries). Each participant saw 60% layperson, 30% expert, and 10% nonjuries given the different number of possible juries by category. Juries were randomized to participants in these proportions in a random order, and juries were equally randomized within each major jury category per participant.

### Legitimacy ratings

Participants first individually evaluated each jury within a jury pair. Participants saw a grid detailing the features of a jury (similar to a column from Fig. 1) and then were asked: “Imagine Jury X made a decision you disagreed with. Please answer the following questions about Jury X.” Participants then answered the following five questions adopted from (46): “I would be satisfied with Jury X handling the evaluation decision”; “Jury X can be trusted”; “Jury X cannot be fair and impartial” (reverse-coded); “Social media platforms should use Jury X to make content moderation decisions”; “Jury X should be the authority making moderation decisions” (1 = strongly disagree, 2 = disagree, 3 = somewhat disagree, 4 = neutral, 5 = somewhat agree, 6 = agree, 7 = strongly agree). As described in (46), these items were included in order to capture five different measures of institutional legitimacy as conceptualized in (12)—outcome satisfaction, trustworthiness, fairness and impartiality, institutional commitment, and decisional jurisdiction, respectively. And as preregistered, these five items were averaged as a combined legitimacy score and used as the primary outcome variable reported throughout the main text. In addition to these five items, participants were also asked the exploratory (nonpreregistered) outcome variable “Jury X has the skill to make accurate moderation decisions” for each jury. This final exploratory variable is not examined in the current work analyzed but is reported here for full methodological transparency.

We calculated Cronbach's alpha to assess the internal reliability between our scale items (originally reported as 0.92 in (46)). For the first jury participants saw, Cronbach's alpha was 0.81 (95% CI = [0.80–0.82]). Cronbach's alpha for the final unique jury participants saw (Jury 20) was similar, calculated as 0.85 (95% CI = [0.84–0.86]). Internal reliability was the same for the final jury participants evaluated (Jury 22), which was a repeated version of Jury 1 (Cronbach's alpha = 0.85, 95% CI = [0.84–0.86]).

### Jury choice

After answering the legitimacy rating items for both juries within a jury pair, participants saw a combined table summarizing the features of both juries (Fig. 1) and were asked “Which jury would you prefer to have evaluate online content?” (Jury X, Jury Y; coded as 1 = jury was chosen, 0 = jury was not chosen). For a visual of this procedure, see Fig. S1. As preregistered, this jury choice outcome variable is also reported in the main text and Supplementary material as a primary outcome variable.

### Procedure

Participants first completed two trivial attention screeners (e.g. captcha), a brief political item on US president age and vice president responsibilities, and an instructional attention item adapted from (59). Participants then received instructions about the conjoint experiment task and what to expect from each content moderation jury, as detailed above. After a single free-response item on legitimacy features, participants began the main experimental task wherein they evaluated 20 juries in 10 pairs on their perceived legitimacy and jury preference. Participants also completed a 21st jury pair, which was identical to the first jury pair received except with the order of juries flipped—this was included to enable robustness checks accounting for intrarespondent reliability as suggested in (60).

### Analysis strategy

As per our preregistration, we conduct separate series of analyses to predict legitimacy rating (the average of our five legitimacy scale items) and jury choice (binary outcome at the jury level; 1 = jury was chosen, 0 = jury was not chosen). We preregistered conducting simplified linear regressions predicting these outcome variables collapsing across specific juries at the nonjury, layperson, and expert level, such that expert juries (no discussion) were our holdout comparison group. This model included as predictors: a dummy for nonjury, a dummy for layperson jury, the interaction between layperson and size (hold out = 3, 30, and 3,000), the interaction between layperson and qualifications, the interaction between layperson and discussion, the interaction between layperson, size, and qualifications, the interaction between layperson, size, and discussion, the interaction between layperson, qualifications, and discussion, the interaction between layperson, size, qualifications, and discussion, and a dummy for discussion; with cluster robust standard errors at the participant level (and two-way cluster robust standard errors at the participant and jury pair level for our choice dependent variable). The reduced form of this model is below in Eq. 1. Our results from these simplified preregistered analyses are reported in full in SI Section S4, and our results largely mirror those from our full models reported in the main text separating our nine specific juries.


(1) $$\begin{array}{c} DV_{\mathrm{legit},\mathrm{choice}}=\beta_{0}+\beta_{1}\mathrm{NonJury}+\beta_{2}\mathrm{Layperson}+\\ \beta_{3}(\mathrm{Layperson}\cdot \mathrm{Size}30)+\\ \beta_{4}(\mathrm{Layperson}\cdot \mathrm{Size}3,000)+\\ \beta_{5}(\mathrm{Layperson}\cdot \mathrm{Qualifications})+\\ \beta_{6}(\mathrm{Layperson}\cdot \mathrm{Discussion})+\\ \beta_{7}(\mathrm{Layperson}\cdot \mathrm{Size}30\cdot \mathrm{Qualifications})+\\ \beta_{8}(\mathrm{Layperson}\cdot \mathrm{Size}3,000\cdot \mathrm{Qualifications})+\\ \beta_{9}(\mathrm{Layperson}\cdot \mathrm{Size}30\cdot \mathrm{Discussion})+\\ \beta_{10}(\mathrm{Layperson}\cdot \mathrm{Size}3,000\cdot \mathrm{Discussion})+\\ \beta_{11}(\mathrm{Layperson}\cdot \mathrm{Qualifications}\cdot \mathrm{Discussion})+\\ \beta_{12}(\mathrm{Layperson}\cdot \mathrm{Size}30\cdot \mathrm{Qualifications}\cdot \mathrm{Discussion})+\\ \beta_{13}(\mathrm{Layperson}\cdot \mathrm{Size}3,000\cdot \mathrm{Qualifications}\cdot \mathrm{Discussion})+\\ \begin{array}{c} \beta_{14}\mathrm{Discussion}+\\ \epsilon \end{array} \end{array}$$

The preregistered main analyses we report in the main text disaggregate by our nine specific juries, specifying domain experts (no discussion) as our holdout jury. The nonjury and layperson dummies in our simplified model are replaced with three-level factors of the specific juries in each category. We also add a two-level factor for the other two expert juries and allow for an interaction between this expert variable and discussion. The reduced form of this main model is below in Eq. 2.


(2) $$\begin{array}{cc} & \begin{array}{c} DV_{\mathrm{legit},\mathrm{choice}}=\beta_{0}+\beta_{1a}\mathrm{Coin}+\beta_{1b}\mathrm{Algo}+\beta_{1c}\mathrm{Zuck}+\\ \beta_{2a}\mathrm{NatRep}+\\ \beta_{2b}\mathrm{SMUsers}+\\ \beta_{2c}\mathrm{PolBal}+\\ \beta_{3a}(\mathrm{NatRep}\cdot \mathrm{Size}30)+\\ \beta_{3b}(\mathrm{SMUsers}\cdot \mathrm{Size}30)+\\ \beta_{3c}(\mathrm{PolBal}\cdot \mathrm{Size}30)+\\ \beta_{4a}(\mathrm{NatRep}\cdot \mathrm{Size}3,000)+\\ \beta_{4b}(\mathrm{SMUsers}\cdot \mathrm{Size}3,000)+\\ \beta_{4c}(\mathrm{PolBal}\cdot \mathrm{Size}3,000)+\\ \beta_{5a}(\mathrm{NatRep}\cdot \mathrm{Qualifications})+\\ \beta_{5b}(\mathrm{SMUsers}\cdot \mathrm{Qualifications})+\\ \beta_{5c}(\mathrm{PolBal}\cdot \mathrm{Qualifications})+\\ \beta_{6a}(\mathrm{NatRep}\cdot \mathrm{Discussion})+\\ \begin{array}{c} \beta_{6b}(\mathrm{SMUsers}\cdot \mathrm{Discussion})+\\ \beta_{6c}(\mathrm{PolBal}\cdot \mathrm{Discussion})+ \end{array} \end{array}\\ & \begin{array}{c} \begin{array}{c} \beta_{7a}(\mathrm{NatRep}\cdot \mathrm{Size}30\cdot \mathrm{Qualifications})+\\ \beta_{7b}(\mathrm{SMUsers}\cdot \mathrm{Size}30\cdot \mathrm{Qualifications})+\\ \beta_{7c}(\mathrm{PolBal}\cdot \mathrm{Size}30\cdot \mathrm{Qualifications})+ \end{array}\\ \beta_{8a}(\mathrm{NatRep}\cdot \mathrm{Size}3,000\cdot \mathrm{Qualifications})+\\ \beta_{8b}(\mathrm{SMUsers}\cdot \mathrm{Size}3,000\cdot \mathrm{Qualifications})+\\ \beta_{8c}(\mathrm{PolBal}\cdot \mathrm{Size}3,000\cdot \mathrm{Qualifications})+\\ \beta_{9a}(\mathrm{NatRep}\cdot \mathrm{Size}30\cdot \mathrm{Discussion})+\\ \beta_{9b}(\mathrm{SMUsers}\cdot \mathrm{Size}30\cdot \mathrm{Discussion})+\\ \beta_{9c}(\mathrm{PolBal}\cdot \mathrm{Size}30\cdot \mathrm{Discussion})+\\ \beta_{10a}(\mathrm{NatRep}\cdot \mathrm{Size}3,000\cdot \mathrm{Discussion})+\\ \beta_{10b}(\mathrm{SMUsers}\cdot \mathrm{Size}3,000\cdot \mathrm{Discussion})+\\ \beta_{10c}(\mathrm{PolBal}\cdot \mathrm{Size}3,000\cdot \mathrm{Discussion})+\\ \beta_{11a}(\mathrm{NatRep}\cdot \mathrm{Qualifications}\cdot \mathrm{Discussion})+\\ \beta_{11b}(\mathrm{SMUsers}\cdot \mathrm{Qualifications}\cdot \mathrm{Discussion})+\\ \beta_{11c}(\mathrm{PolBal}\cdot \mathrm{Qualifications}\cdot \mathrm{Discussion})+ \end{array}\\ & \begin{array}{c} \beta_{12a}(\mathrm{NatRep}\cdot \mathrm{Size}30\cdot \mathrm{Qualifications}\cdot \mathrm{Discussion})+\\ \beta_{12b}(\mathrm{SMUsers}\cdot \mathrm{Size}30\cdot \mathrm{Qualifications}\cdot \mathrm{Discussion})+\\ \beta_{12c}(\mathrm{PolBal}\cdot \mathrm{Size}30\cdot \mathrm{Qualifications}\cdot \mathrm{Discussion})+\\ \beta_{13a}(\mathrm{NatRep}\cdot \mathrm{Size}3,000\cdot \mathrm{Qualifications}\cdot \mathrm{Discussion})+\\ \beta_{13b}(\mathrm{SMUsers}\cdot \mathrm{Size}3,000\cdot \mathrm{Qualifications}\cdot \mathrm{Discussion})+\\ \beta_{13c}(\mathrm{PolBal}\cdot \mathrm{Size}3,000\cdot \mathrm{Qualifications}\cdot \mathrm{Discussion})+\\ \beta_{14}Discussion+\\ \beta_{15}FCers+\\ \beta_{16}Journ+\\ \beta_{17a}(\mathrm{FCers}\cdot \mathrm{Discussion})+\\ \beta_{17b}(\mathrm{Journ}\cdot \mathrm{Discussion})+\\ \epsilon \end{array} \end{array}$$

Our preregistration did not specify whether we would analytically weight our analyses by the general population weight provided by YouGov based on their propensity score function (considering age, gender, race/ethnicity, years of education, region, and presidential vote choice). We report sample estimates in our main text, but all analyses are reproduced with general population weighting in our Supplementary tables—all results remain highly similar.

As preregistered, we also conduct analyses including partisanship as a potential moderator. Partisanship was classified according to 7-point party ID, and then separated into Democrats, Republicans, and true Independents (Democrat if 7-point party ID included “Democrat”; Republican if 7-point party ID included “Republican”; Independent if party ID was “Independent”).

We also conducted robustness checks filtering for highly attentive participants (assessed pretreatment) and accounting for intrarespondent reliability. All results were largely robust to both assessments (SI Sections S6–S8).

## Supplementary Material

pgaf111_Supplementary_Data

## Preprints

This manuscript was posted as a preprint at: https://osf.io/preprints/psyarxiv/5n9tp_v1.

### Data Availability

Preregistration, survey materials, data, and analysis code are all available on OSF: https://osf.io/7tjhb/. Participants provided informed consent, and our studies were determined exempt by the Massachusetts Institute of Technology Committee on the Use of Humans as Experimental Subjects form E-3646.
