# Supplementary material for: Perceived legitimacy of layperson and expert content moderators
Source: PNAS Nexus. 2025 May 20;4(5):pgaf111. doi: 10.1093/pnasnexus/pgaf111 (PMC12063528; doi:10.1093/pnasnexus/pgaf111)
Supplement: pgaf111_Supplementary_Data [file pgaf111_supplementary_data.pdf]

Supplemental Information

for

"Perceived legitimacy of layperson and expert content moderators"

Martel et al.

Table of Contents.

1. Supplemental Methods.
2. Main Analyses – Results.
3. Partisanship Moderation Analyses – Results.
4. Supplemental Main Results - Collapsed Jury Type Analyses.
5. Additional Supplemental Figures.
6. Secondary Analyses – Attention Filter.
7. Partisanship Moderation Analyses – Attention Filter.
8. Secondary Analyses – Measurement Error Corrections.
9. References.

1. Supplemental Methods.

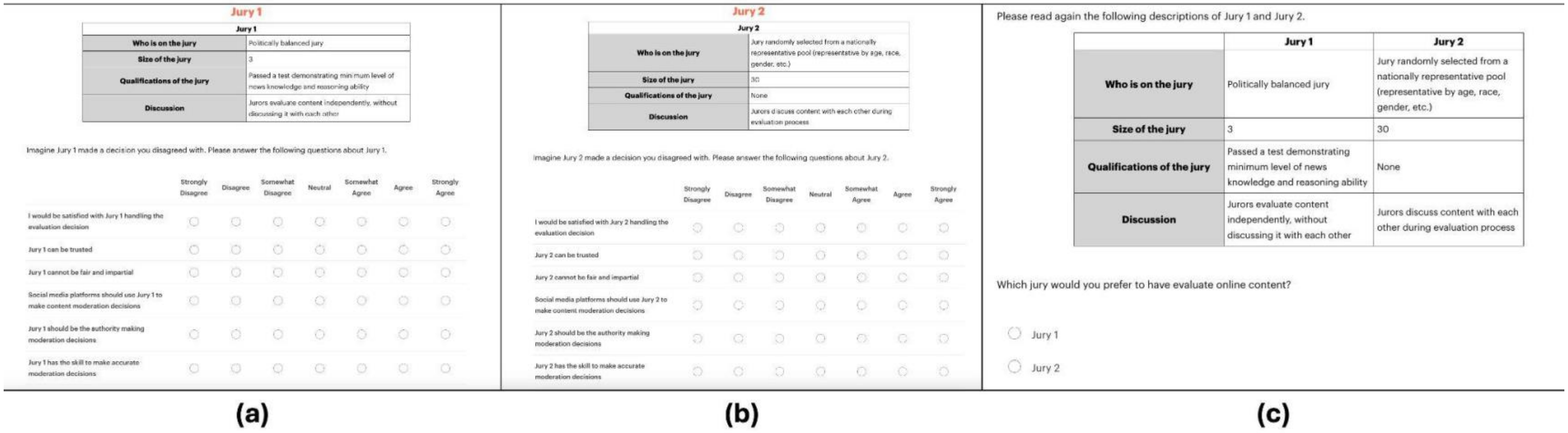

Figure S1. Conjoint survey task procedure. (a) Participants first evaluate Likert-scale legitimacy items for a single jury. (b) Participants next evaluate Likert-scale legitimacy items for a second jury. (c) Participants then see a combined table re-summarizing both jury features and are asked to choose which jury they would prefer to have evaluate online content. Participants complete this procedure 10 unique times (as well as an 11th repeated pair at the end to estimate intra-respondent reliability).

2. Main Analyses – Results.

Table S1. *Legitimacy rating or jury choice predicted by category-level jury types and features. Columns (2) and (4) show results with analytic weighting by population-weights.*

	Dependent variable:			
	Legitimacy (1-7) Sample	Legitimacy (1-7) Population	Choice Sample	Choice Population
	(1)	(2)	(3)	(4)
NonJury	-1.165***	-1.168***	-0.342***	-0.340***
	(0.024)	(0.025)	(0.010)	(0.010)
	t = -48.606	t = -45.916	t = -35.258	t = -33.685
	p = 0.000	p = 0.000	p = 0.000	p = 0.000
Layperson	-0.603***	-0.614***	-0.216***	-0.216***
	(0.027)	(0.028)	(0.011)	(0.011)
	t = -22.138	t = -21.774	t = -18.830	t = -19.351
	p = 0.000	p = 0.000	p = 0.000	p = 0.000
Discuss	0.129***	0.131***	0.053***	0.053***
	(0.012)	(0.013)	(0.006)	(0.007)
	t = 10.863	t = 10.125	t = 9.282	t = 7.766
	p = 0.000	p = 0.000	p = 0.000	p = 0.000
Layperson:Size30	0.296***	0.300***	0.124***	0.122***
	(0.027)	(0.028)	(0.012)	(0.010)
	t = 10.851	t = 10.675	t = 10.799	t = 12.686
	p = 0.000	p = 0.000	p = 0.000	p = 0.000
Layperson:Size3000	0.323***	0.326***	0.158***	0.153***
	(0.028)	(0.029)	(0.012)	(0.012)

	t = 11.478	t = 11.356	t = 13.305	t = 12.333
	p = 0.000	p = 0.000	p = 0.000	p = 0.000
Layperson:Qual	0.276***	0.276***	0.129***	0.127***
	(0.027)	(0.028)	(0.008)	(0.009)
	t = 10.313	t = 9.818	t = 16.342	t = 13.896
	p = 0.000	p = 0.000	p = 0.000	p = 0.000
Layperson:Discuss	0.014	0.025	0.0001	0.002
	(0.028)	(0.029)	(0.012)	(0.012)
	t = 0.515	t = 0.844	t = 0.008	t = 0.205
	p = 0.607	p = 0.399	p = 0.994	p = 0.838
Layperson:Size30:Qual	0.008	0.018	0.024	0.021
	(0.037)	(0.039)	(0.017)	(0.015)
	t = 0.209	t = 0.470	t = 1.462	t = 1.441
	p = 0.835	p = 0.639	p = 0.144	p = 0.150
Layperson:Size3000:Qual	0.029	0.033	0.018	0.019
	(0.038)	(0.040)	(0.016)	(0.017)
	t = 0.764	t = 0.819	t = 1.081	t = 1.140
	p = 0.446	p = 0.413	p = 0.280	p = 0.255
Layperson:Size30:Discuss	0.00001	-0.013	-0.0004	0.001
	(0.037)	(0.039)	(0.017)	(0.016)
	t = 0.0004	t = -0.344	t = -0.022	t = 0.032
	p = 1.000	p = 0.731	p = 0.983	p = 0.975
Layperson:Size3000:Discuss	-0.013	-0.011	-0.021	-0.019
	(0.037)	(0.039)	(0.017)	(0.015)
	t = -0.355	t = -0.270	t = -1.275	t = -1.201
	p = 0.723	p = 0.788	p = 0.203	p = 0.230
Layperson:Qual:Discuss	-0.014	-0.002	-0.003	0.001
	(0.038)	(0.040)	(0.010)	(0.010)
	t = -0.369	t = -0.060	t = -0.262	t = 0.124
	p = 0.712	p = 0.953	p = 0.794	p = 0.902
Layperson:Size30:Qual:Discuss	-0.001	-0.006	-0.008	-0.007
	(0.051)	(0.055)	(0.019)	(0.019)

	t = -0.011	t = -0.113	t = -0.393	t = -0.389
	p = 0.992	p = 0.911	p = 0.695	p = 0.698
Layperson:Size3000:Qual:Discuss	-0.009	-0.028	-0.014	-0.021
	(0.055)	(0.058)	(0.022)	(0.022)
	t = -0.169	t = -0.486	t = -0.641	t = -0.969
	p = 0.867	p = 0.628	p = 0.522	p = 0.333
Constant	4.007***	4.009***	0.544***	0.545***
	(0.021)	(0.022)	(0.006)	(0.006)
	t = 191.954	t = 182.139	t = 84.128	t = 92.019
	p = 0.000	p = 0.000	p = 0.000	p = 0.000

Note: *p<.05, **p<.01, ***p<.001

OLS predicting legitimacy or choice of juries by non-jury, layperson, and expert categories. Baseline levels are expert jury, size of 3, no qualifications or discussion.

Table S2. *Legitimacy rating or jury choice predicted by jury-specific levels and features. Columns (2) and (4) show results with analytic weighting by population-weights.*

	Dependent variable:			
	Legitimacy (1-7) Sample	Legitimacy (1-7) Population	Choice Sample	Choice Population
	(1)	(2)	(3)	(4)
Coin	-1.393***	-1.399***	-0.415***	-0.421***
	(0.034)	(0.035)	(0.011)	(0.012)
	t = -41.359	t = -39.563	t = -36.602	t = -35.508
	p = 0.000	p = 0.000	p = 0.000	p = 0.000
Algo	-1.201***	-1.204***	-0.369***	-0.374***
	(0.033)	(0.035)	(0.018)	(0.019)
	t = -36.775	t = -34.808	t = -20.330	t = -19.600
	p = 0.000	p = 0.000	p = 0.000	p = 0.000
Zuck	-1.385***	-1.370***	-0.417***	-0.418***
	(0.035)	(0.037)	(0.010)	(0.011)
	t = -39.276	t = -36.557	t = -43.762	t = -37.980
	p = 0.000	p = 0.000	p = 0.000	p = 0.000
NatRep	-0.725***	-0.734***	-0.259***	-0.272***
	(0.040)	(0.042)	(0.022)	(0.024)
	t = -18.060	t = -17.544	t = -11.613	t = -11.185
	p = 0.000	p = 0.000	p = 0.000	p = 0.000
SMUsers	-1.016***	-1.016***	-0.327***	-0.332***
	(0.042)	(0.044)	(0.018)	(0.019)
	t = -24.062	t = -22.934	t = -18.601	t = -17.472
	p = 0.000	p = 0.000	p = 0.000	p = 0.000
PolBal	-0.558***	-0.567***	-0.240***	-0.237***

	(0.038)	(0.039)	(0.027)	(0.029)
	t = -14.615	t = -14.413	t = -8.963	t = -8.202
	p = 0.000	p = 0.000	p = 0.000	p = 0.000
FCers	-0.035	-0.028	-0.021**	-0.031***
	(0.020)	(0.021)	(0.008)	(0.007)
	t = -1.766	t = -1.365	t = -2.676	t = -4.118
	p = 0.078	p = 0.173	p = 0.008	p = 0.00004
Journ	-0.452***	-0.444***	-0.156***	-0.162***
	(0.021)	(0.023)	(0.012)	(0.015)
	t = -21.113	t = -19.518	t = -13.203	t = -10.483
	p = 0.000	p = 0.000	p = 0.000	p = 0.000
Discuss	0.163***	0.161***	0.057***	0.052***
	(0.017)	(0.019)	(0.006)	(0.010)
	t = 9.463	t = 8.586	t = 10.367	t = 5.335
	p = 0.000	p = 0.000	p = 0.000	p = 0.00000
NatRep:Size30	0.326***	0.343***	0.130***	0.136***
	(0.048)	(0.050)	(0.028)	(0.026)
	t = 6.762	t = 6.920	t = 4.662	t = 5.263
	p = 0.000	p = 0.000	p = 0.00001	p = 0.00000
NatRep:Size3000	0.373***	0.386***	0.190***	0.191***
	(0.047)	(0.050)	(0.026)	(0.028)
	t = 7.885	t = 7.670	t = 7.207	t = 6.893
	p = 0.000	p = 0.000	p = 0.000	p = 0.000
SMUsers:Size30	0.369***	0.362***	0.124***	0.114***
	(0.050)	(0.052)	(0.019)	(0.020)
	t = 7.338	t = 6.905	t = 6.466	t = 5.815
	p = 0.000	p = 0.000	p = 0.000	p = 0.000
SMUsers:Size3000	0.438***	0.403***	0.163***	0.163***
	(0.049)	(0.052)	(0.026)	(0.026)
	t = 8.934	t = 7.794	t = 6.179	t = 6.203
	p = 0.000	p = 0.000	p = 0.000	p = 0.000
PolBal:Size30	0.196***	0.195***	0.121***	0.115***

	(0.044)	(0.045)	(0.027)	(0.027)
	t = 4.456	t = 4.297	t = 4.539	t = 4.306
	p = 0.00001	p = 0.00002	p = 0.00001	p = 0.00002
PolBal:Size3000	0.157***	0.187***	0.121***	0.105***
	(0.047)	(0.049)	(0.022)	(0.024)
	t = 3.330	t = 3.830	t = 5.477	t = 4.299
	p = 0.001	p = 0.0002	p = 0.00000	p = 0.00002
NatRep:Qual	0.292***	0.294***	0.129***	0.135***
	(0.047)	(0.049)	(0.020)	(0.023)
	t = 6.265	t = 6.004	t = 6.363	t = 5.865
	p = 0.000	p = 0.000	p = 0.000	p = 0.000
SMUsers:Qual	0.355***	0.348***	0.124***	0.123***
	(0.050)	(0.053)	(0.020)	(0.025)
	t = 7.096	t = 6.605	t = 6.070	t = 4.904
	p = 0.000	p = 0.000	p = 0.000	p = 0.00000
PolBal:Qual	0.183***	0.184***	0.133***	0.122***
	(0.048)	(0.049)	(0.030)	(0.035)
	t = 3.797	t = 3.742	t = 4.416	t = 3.511
	p = 0.0002	p = 0.0002	p = 0.00002	p = 0.0005
NatRep:Discuss	0.017	0.026	-0.014	0.001
	(0.049)	(0.053)	(0.026)	(0.029)
	t = 0.345	t = 0.490	t = -0.538	t = 0.025
	p = 0.731	p = 0.625	p = 0.591	p = 0.980
SMUsers:Discuss	0.093	0.103*	0.026	0.030
	(0.050)	(0.052)	(0.030)	(0.033)
	t = 1.865	t = 1.968	t = 0.892	t = 0.918
	p = 0.063	p = 0.050	p = 0.373	p = 0.359
PolBal:Discuss	-0.164**	-0.146**	-0.024	-0.020
	(0.051)	(0.053)	(0.032)	(0.033)
	t = -3.196	t = -2.737	t = -0.757	t = -0.590
	p = 0.002	p = 0.007	p = 0.449	p = 0.556

Discuss:FCers	-0.026	-0.026	0.005	0.013
	(0.022)	(0.024)	(0.012)	(0.010)
	t = -1.145	t = -1.093	t = 0.406	t = 1.259
	p = 0.253	p = 0.275	p = 0.685	p = 0.208
Discuss:Journ	-0.075***	-0.063**	-0.017	-0.009
	(0.022)	(0.024)	(0.010)	(0.013)
	t = -3.345	t = -2.581	t = -1.662	t = -0.650
	p = 0.001	p = 0.010	p = 0.097	p = 0.516
NatRep:Size30:Qual	-0.049	-0.051	0.025	0.020
	(0.069)	(0.073)	(0.033)	(0.035)
	t = -0.706	t = -0.694	t = 0.742	t = 0.587
	p = 0.480	p = 0.488	p = 0.458	p = 0.557
NatRep:Size3000:Qual	-0.022	-0.031	-0.004	-0.012
	(0.067)	(0.070)	(0.037)	(0.044)
	t = -0.322	t = -0.435	t = -0.100	t = -0.282
	p = 0.748	p = 0.664	p = 0.921	p = 0.778
SMUsers:Size30:Qual	-0.033	-0.006	0.038	0.033
	(0.064)	(0.068)	(0.029)	(0.035)
	t = -0.518	t = -0.094	t = 1.329	t = 0.954
	p = 0.605	p = 0.925	p = 0.184	p = 0.341
SMUsers:Size3000:Qual	-0.040	0.005	0.028	0.023
	(0.068)	(0.072)	(0.038)	(0.038)
	t = -0.598	t = 0.068	t = 0.742	t = 0.599
	p = 0.551	p = 0.946	p = 0.459	p = 0.550
PolBal:Size30:Qual	0.103	0.112	0.010	0.011
	(0.062)	(0.065)	(0.046)	(0.047)
	t = 1.658	t = 1.721	t = 0.217	t = 0.231
	p = 0.098	p = 0.086	p = 0.829	p = 0.818
PolBal:Size3000:Qual	0.150*	0.134	0.029	0.051
	(0.067)	(0.069)	(0.025)	(0.032)
	t = 2.248	t = 1.930	t = 1.142	t = 1.575
	p = 0.025	p = 0.054	p = 0.254	p = 0.116
NatRep:Size30:Discuss	0.018	0.005	0.009	-0.004
	(0.067)	(0.071)	(0.034)	(0.032)

	t = 0.268	t = 0.075	t = 0.260	t = -0.113
	p = 0.789	p = 0.941	p = 0.796	p = 0.911
NatRep:Size3000:Discuss	-0.059	-0.046	-0.054	-0.058
	(0.067)	(0.071)	(0.036)	(0.037)
	t = -0.891	t = -0.644	t = -1.482	t = -1.578
	p = 0.374	p = 0.520	p = 0.139	p = 0.115
SMUsers:Size30:Discuss	-0.146*	-0.131	-0.033	-0.015
	(0.069)	(0.071)	(0.031)	(0.031)
	t = -2.101	t = -1.835	t = -1.070	t = -0.494
	p = 0.036	p = 0.067	p = 0.285	p = 0.622
SMUsers:Size3000:Discuss	-0.136*	-0.100	-0.071	-0.070
	(0.067)	(0.071)	(0.039)	(0.038)
	t = -2.038	t = -1.403	t = -1.819	t = -1.869
	p = 0.042	p = 0.161	p = 0.069	p = 0.062
PolBal:Size30:Discuss	0.127	0.095	0.023	0.023
	(0.067)	(0.071)	(0.039)	(0.047)
	t = 1.881	t = 1.344	t = 0.588	t = 0.488
	p = 0.061	p = 0.180	p = 0.557	p = 0.626
PolBal:Size3000:Discuss	0.155*	0.125	0.063	0.076*
	(0.068)	(0.072)	(0.033)	(0.034)
	t = 2.280	t = 1.735	t = 1.926	t = 2.225
	p = 0.023	p = 0.083	p = 0.055	p = 0.027
NatRep:Qual:Discuss	-0.036	-0.024	0.036	0.034
	(0.066)	(0.072)	(0.025)	(0.030)
	t = -0.554	t = -0.338	t = 1.464	t = 1.109
	p = 0.580	p = 0.736	p = 0.144	p = 0.268
SMUsers:Qual:Discuss	-0.123	-0.108	-0.041	-0.029
	(0.074)	(0.078)	(0.024)	(0.022)
	t = -1.666	t = -1.376	t = -1.729	t = -1.320
	p = 0.096	p = 0.169	p = 0.084	p = 0.187
PolBal:Qual:Discuss	0.116	0.133	-0.002	0.001
	(0.067)	(0.069)	(0.042)	(0.049)
	t = 1.747	t = 1.926	t = -0.055	t = 0.029
	p = 0.081	p = 0.055	p = 0.956	p = 0.977

NatRep:Size30:Qual:Discuss	0.037	0.037	-0.041	-0.034
	(0.096)	(0.104)	(0.033)	(0.040)
	t = 0.389	t = 0.357	t = -1.268	t = -0.846
	p = 0.698	p = 0.721	p = 0.205	p = 0.398
NatRep:Size3000:Qual:Discuss	0.037	0.001	-0.020	-0.026
	(0.097)	(0.104)	(0.041)	(0.051)
	t = 0.380	t = 0.005	t = -0.497	t = -0.518
	p = 0.704	p = 0.997	p = 0.619	p = 0.605
SMUsers:Size30:Qual:Discuss	0.149	0.109	0.029	0.017
	(0.098)	(0.103)	(0.034)	(0.034)
	t = 1.518	t = 1.063	t = 0.858	t = 0.487
	p = 0.129	p = 0.288	p = 0.391	p = 0.627
SMUsers:Size3000:Qual:Discuss	0.091	0.044	0.053	0.040
	(0.098)	(0.104)	(0.054)	(0.050)
	t = 0.928	t = 0.422	t = 0.981	t = 0.801
	p = 0.354	p = 0.674	p = 0.327	p = 0.424
PolBal:Size30:Qual:Discuss	-0.184^{*}	-0.177	-0.010	-0.010
	(0.088)	(0.094)	(0.055)	(0.061)
	t = -2.085	t = -1.888	t = -0.183	t = -0.157
	p = 0.038	p = 0.059	p = 0.856	p = 0.876
PolBal:Size3000:Qual:Discuss	-0.161	-0.153	-0.078^{**}	-0.087^{*}
	(0.096)	(0.102)	(0.030)	(0.041)
	t = -1.668	t = -1.505	t = -2.613	t = -2.129
	p = 0.096	p = 0.133	p = 0.009	p = 0.034
Constant	4.170^{***}	4.166^{***}	0.603^{***}	0.609^{***}
	(0.022)	(0.023)	(0.008)	(0.010)
	t = 186.895	t = 177.521	t = 72.428	t = 62.961
	p = 0.000	p = 0.000	p = 0.000	p = 0.000

Note: $^{*}p<.05, ^{**}p<.01, ^{***}p<.001$

OLS predicting legitimacy or choice of juries by multiple jury categories. Baseline levels are domain expert jury, size of 3, no qualifications or discussion.

3. Partisanship Moderation Analyses – Results.

Table S3. *Legitimacy rating or jury choice predicted by category-level jury types, features, and partisanship. Columns (2) and (4) show results with analytic weighting by population-weights.*

	Dependent variable:			
	Legitimacy (1-7) Sample	Legitimacy (1-7) Population	Choice Sample	Choice Population
	(1)	(2)	(3)	(4)
NonJury	-0.868***	-0.859***	-0.253***	-0.253***
	(0.036)	(0.037)	(0.019)	(0.019)
	t = -23.840	t = -23.062	t = -13.212	t = -13.151
	p = 0.000	p = 0.000	p = 0.000	p = 0.000
Layperson	-0.174***	-0.175***	-0.095***	-0.086***
	(0.044)	(0.045)	(0.022)	(0.025)
	t = -3.928	t = -3.906	t = -4.281	t = -3.369
	p = 0.0001	p = 0.0001	p = 0.00002	p = 0.001
Discuss	0.074***	0.081***	0.030*	0.025
	(0.020)	(0.022)	(0.014)	(0.014)
	t = 3.692	t = 3.714	t = 2.158	t = 1.802
	p = 0.0003	p = 0.0003	p = 0.031	p = 0.072
SubjectPartyInd	0.352***	0.365***	0.069***	0.065***
	(0.060)	(0.062)	(0.017)	(0.018)
	t = 5.899	t = 5.835	t = 4.008	t = 3.553
	p = 0.000	p = 0.000	p = 0.0001	p = 0.0004
SubjectPartyDem	0.900***	0.936***	0.130***	0.131***
	(0.046)	(0.048)	(0.015)	(0.015)
	t = 19.477	t = 19.403	t = 8.968	t = 8.853
	p = 0.000	p = 0.000	p = 0.000	p = 0.000
Layperson:Size30	0.246***	0.239***	0.125***	0.115***
	(0.048)	(0.050)	(0.024)	(0.027)
	t = 5.071	t = 4.742	t = 5.240	t = 4.189
	p = 0.00000	p = 0.00001	p = 0.00000	p = 0.00003

Layperson:Size3000	0.352***	0.351***	0.172***	0.159***
	(0.052)	(0.052)	(0.022)	(0.022)
	t = 6.783	t = 6.737	t = 7.982	t = 7.341
	p = 0.000	p = 0.000	p = 0.000	p = 0.000
Layperson:Qual	0.200***	0.188***	0.121***	0.108***
	(0.050)	(0.054)	(0.022)	(0.026)
	t = 4.002	t = 3.483	t = 5.496	t = 4.119
	p = 0.0001	p = 0.0005	p = 0.00000	p = 0.00004
Layperson:Discuss	-0.003	-0.003	0.025	0.016
	(0.048)	(0.050)	(0.022)	(0.023)
	t = -0.068	t = -0.056	t = 1.115	t = 0.706
	p = 0.946	p = 0.956	p = 0.265	p = 0.480
NonJury:SubjectPartyInd	-0.156*	-0.161*	-0.087**	-0.087**
	(0.064)	(0.067)	(0.027)	(0.028)
	t = -2.455	t = -2.412	t = -3.173	t = -3.063
	p = 0.015	p = 0.016	p = 0.002	p = 0.003
NonJury:SubjectPartyDem	-0.631***	-0.656***	-0.167***	-0.167***
	(0.053)	(0.055)	(0.022)	(0.022)
	t = -11.933	t = -11.846	t = -7.485	t = -7.684
	p = 0.000	p = 0.000	p = 0.000	p = 0.000
Layperson:SubjectPartyInd	-0.324***	-0.346***	-0.114***	-0.126***
	(0.073)	(0.075)	(0.031)	(0.036)
	t = -4.411	t = -4.606	t = -3.618	t = -3.469
	p = 0.00002	p = 0.00001	p = 0.0003	p = 0.001
Layperson:SubjectPartyDem	-0.842***	-0.851***	-0.213***	-0.228***
	(0.061)	(0.062)	(0.029)	(0.031)
	t = -13.904	t = -13.639	t = -7.351	t = -7.360
	p = 0.000	p = 0.000	p = 0.000	p = 0.000
Discuss:SubjectPartyInd	0.010	0.011	0.023	0.043
	(0.032)	(0.035)	(0.018)	(0.022)
	t = 0.319	t = 0.315	t = 1.261	t = 1.947
	p = 0.750	p = 0.753	p = 0.208	p = 0.052
Discuss:SubjectPartyDem	0.111***	0.092**	0.038*	0.039
	(0.028)	(0.030)	(0.018)	(0.020)
	t = 4.041	t = 3.097	t = 2.099	t = 1.925
	p = 0.0001	p = 0.002	p = 0.036	p = 0.055
Layperson:Size30:Qual	0.079	0.107	0.004	0.008
	(0.064)	(0.071)	(0.042)	(0.046)

	t = 1.234	t = 1.514	t = 0.084	t = 0.173
	p = 0.218	p = 0.131	p = 0.933	p = 0.863
Layperson:Size3000:Qual	0.008	0.036	-0.015	0.0003
	(0.069)	(0.074)	(0.026)	(0.030)
	t = 0.119	t = 0.493	t = -0.568	t = 0.011
	p = 0.906	p = 0.623	p = 0.571	p = 0.992
Layperson:Size30:Discuss	0.063	0.103	-0.010	0.007
	(0.064)	(0.068)	(0.023)	(0.030)
	t = 0.979	t = 1.519	t = -0.449	t = 0.248
	p = 0.328	p = 0.129	p = 0.654	p = 0.805
Layperson:Size3000:Discuss	-0.047	-0.020	-0.057**	-0.035
	(0.066)	(0.069)	(0.018)	(0.022)
	t = -0.715	t = -0.292	t = -3.173	t = -1.631
	p = 0.475	p = 0.771	p = 0.002	p = 0.103
Layperson:Qual:Discuss	0.040	0.059	-0.049	-0.026
	(0.067)	(0.073)	(0.027)	(0.031)
	t = 0.598	t = 0.798	t = -1.784	t = -0.847
	p = 0.550	p = 0.425	p = 0.075	p = 0.397
Layperson:Size30:SubjectPartyInd	-0.020	0.010	0.027	0.037
	(0.076)	(0.079)	(0.035)	(0.048)
	t = -0.268	t = 0.133	t = 0.779	t = 0.766
	p = 0.789	p = 0.895	p = 0.436	p = 0.444
Layperson:Size3000:SubjectPartyInd	-0.130	-0.118	-0.009	0.004
	(0.081)	(0.082)	(0.032)	(0.034)
	t = -1.617	t = -1.449	t = -0.280	t = 0.126
	p = 0.106	p = 0.148	p = 0.780	p = 0.900
Layperson:Size30:SubjectPartyDem	0.142*	0.149*	-0.022	-0.010
	(0.064)	(0.066)	(0.025)	(0.028)
	t = 2.227	t = 2.263	t = -0.891	t = -0.349
	p = 0.026	p = 0.024	p = 0.374	p = 0.728
Layperson:Size3000:SubjectPartyDem	0.006	0.008	-0.041	-0.025
	(0.066)	(0.068)	(0.032)	(0.030)
	t = 0.094	t = 0.122	t = -1.304	t = -0.835
	p = 0.926	p = 0.903	p = 0.193	p = 0.404
Layperson:Qual:SubjectPartyInd	0.100	0.139	0.006	0.022
	(0.079)	(0.081)	(0.041)	(0.046)
	t = 1.269	t = 1.705	t = 0.137	t = 0.484
	p = 0.205	p = 0.089	p = 0.891	p = 0.629
Layperson:Qual:SubjectPartyDem	0.129*	0.138*	0.007	0.025

	(0.063)	(0.067)	(0.032)	(0.040)
	t = 2.034	t = 2.048	t = 0.224	t = 0.641
	p = 0.042	p = 0.041	p = 0.823	p = 0.522
Layperson:Discuss:SubjectPartyInd	0.083	0.107	-0.038	-0.034
	(0.078)	(0.081)	(0.052)	(0.050)
	t = 1.064	t = 1.317	t = -0.728	t = -0.682
	p = 0.288	p = 0.188	p = 0.467	p = 0.496
Layperson:Discuss:SubjectPartyDem	0.009	0.020	-0.053	-0.035
	(0.065)	(0.067)	(0.032)	(0.031)
	t = 0.141	t = 0.301	t = -1.654	t = -1.133
	p = 0.888	p = 0.764	p = 0.099	p = 0.258
Layperson:Size30:Qual:Discuss	-0.078	-0.137	0.038	0.019
	(0.088)	(0.101)	(0.041)	(0.051)
	t = -0.887	t = -1.367	t = 0.915	t = 0.362
	p = 0.376	p = 0.172	p = 0.361	p = 0.718
Layperson:Size3000:Qual:Discuss	0.0003	-0.056	0.068**	0.038
	(0.100)	(0.105)	(0.026)	(0.035)
	t = 0.003	t = -0.533	t = 2.579	t = 1.108
	p = 0.998	p = 0.595	p = 0.010	p = 0.268
Layperson:Size30:Qual:SubjectPartyInd	-0.099	-0.160	-0.015	-0.024
	(0.104)	(0.110)	(0.062)	(0.077)
	t = -0.952	t = -1.461	t = -0.234	t = -0.313
	p = 0.342	p = 0.144	p = 0.815	p = 0.755
Layperson:Size3000:Qual:SubjectPartyInd	-0.023	-0.069	0.017	0.007
	(0.108)	(0.114)	(0.052)	(0.057)
	t = -0.214	t = -0.608	t = 0.322	t = 0.123
	p = 0.831	p = 0.543	p = 0.748	p = 0.902
Layperson:Size30:Qual:SubjectPartyDem	-0.109	-0.115	0.063	0.052
	(0.085)	(0.091)	(0.050)	(0.058)
	t = -1.277	t = -1.264	t = 1.254	t = 0.896
	p = 0.202	p = 0.207	p = 0.210	p = 0.371
Layperson:Size3000:Qual:SubjectPartyDem	0.090	0.055	0.078*	0.053
	(0.089)	(0.095)	(0.039)	(0.049)
	t = 1.004	t = 0.575	t = 1.986	t = 1.070
	p = 0.316	p = 0.566	p = 0.048	p = 0.285
Layperson:Size30:Discuss:SubjectPartyInd	-0.061	-0.164	-0.052	-0.076
	(0.104)	(0.110)	(0.059)	(0.065)
	t = -0.589	t = -1.486	t = -0.888	t = -1.162
	p = 0.557	p = 0.138	p = 0.375	p = 0.246

Layperson:Size3000:Discuss:SubjectPartyInd	0.014	-0.024	0.038	0.008
	(0.104)	(0.107)	(0.059)	(0.053)
	t = 0.138	t = -0.226	t = 0.642	t = 0.141
	p = 0.891	p = 0.822	p = 0.521	p = 0.888
Layperson:Size30:Discuss:SubjectPartyDem	-0.112	-0.179*	0.064*	0.046
	(0.087)	(0.090)	(0.032)	(0.045)
	t = -1.296	t = -2.004	t = 2.010	t = 1.036
	p = 0.196	p = 0.046	p = 0.045	p = 0.301
Layperson:Size3000:Discuss:SubjectPartyDem	0.092	0.059	0.080*	0.052
	(0.088)	(0.092)	(0.037)	(0.041)
	t = 1.045	t = 0.637	t = 2.148	t = 1.264
	p = 0.297	p = 0.525	p = 0.032	p = 0.207
Layperson:Qual:Discuss:SubjectPartyInd	-0.218*	-0.265*	0.071	0.039
	(0.108)	(0.116)	(0.067)	(0.074)
	t = -2.025	t = -2.281	t = 1.065	t = 0.535
	p = 0.043	p = 0.023	p = 0.287	p = 0.593
Layperson:Qual:Discuss:SubjectPartyDem	-0.022	-0.015	0.085*	0.063
	(0.088)	(0.094)	(0.038)	(0.046)
	t = -0.254	t = -0.157	t = 2.261	t = 1.352
	p = 0.800	p = 0.876	p = 0.024	p = 0.177
Layperson:Size30:Qual:Discuss:SubjectPartyInd	0.199	0.332*	0.015	0.052
	(0.140)	(0.158)	(0.079)	(0.095)
	t = 1.417	t = 2.098	t = 0.191	t = 0.546
	p = 0.157	p = 0.036	p = 0.849	p = 0.586
Layperson:Size3000:Qual:Discuss:SubjectPartyInd	0.172	0.251	-0.105	-0.075
	(0.154)	(0.162)	(0.071)	(0.075)
	t = 1.113	t = 1.550	t = -1.488	t = -1.006
	p = 0.266	p = 0.122	p = 0.137	p = 0.315
Layperson:Size30:Qual:Discuss:SubjectPartyDem	0.104	0.157	-0.120	-0.101
	(0.120)	(0.129)	(0.065)	(0.080)
	t = 0.871	t = 1.212	t = -1.833	t = -1.261
	p = 0.384	p = 0.226	p = 0.067	p = 0.208
Layperson:Size3000:Qual:Discuss:SubjectPartyDem	-0.120	-0.079	-0.146*	-0.114
	(0.130)	(0.137)	(0.057)	(0.074)
	t = -0.925	t = -0.573	t = -2.540	t = -1.549
	p = 0.356	p = 0.567	p = 0.012	p = 0.122
Constant	3.540***	3.521***	0.474***	0.475***
	(0.038)	(0.040)	(0.012)	(0.012)
	t = 92.508	t = 87.880	t = 39.980	t = 38.941
	p = 0.000	p = 0.000	p = 0.000	p = 0.000

Note: $^{*}p<.05, ^{**}p<.01, ^{***}p<.001$

OLS predicting legitimacy or choice of juries by non-jury, layperson, and expert categories, with partisanship moderator. Baseline levels are expert jury, size of 3, no qualifications or discussion; and Republican party.

Table S4. *Legitimacy rating or jury choice predicted by jury-specific levels, features, and partisanship. Columns (2) and (4) show results with analytic weighting by population-weights.*

	Dependent variable:			
	Legitimacy (1-7) Sample	Legitimacy (1-7) Population	Choice Sample	Choice Population
	(1)	(2)	(3)	(4)
Coin	-1.143***	-1.137***	-0.348***	-0.356***
	(0.056)	(0.057)	(0.022)	(0.019)
	t = -20.489	t = -19.965	t = -15.905	t = -19.246
	p = 0.000	p = 0.000	p = 0.000	p = 0.000
Algo	-1.022***	-1.003***	-0.338***	-0.336***
	(0.054)	(0.055)	(0.029)	(0.026)
	t = -18.952	t = -18.142	t = -11.577	t = -12.758
	p = 0.000	p = 0.000	p = 0.000	p = 0.000
Zuck	-1.327***	-1.312***	-0.401***	-0.409***
	(0.058)	(0.061)	(0.027)	(0.024)
	t = -23.032	t = -21.589	t = -14.981	t = -17.099
	p = 0.000	p = 0.000	p = 0.000	p = 0.000
NatRep	-0.448***	-0.473***	-0.192***	-0.195***
	(0.071)	(0.075)	(0.031)	(0.034)
	t = -6.288	t = -6.262	t = -6.184	t = -5.693
	p = 0.000	p = 0.000	p = 0.000	p = 0.000
SMUsers	-0.640***	-0.628***	-0.248***	-0.240***
	(0.071)	(0.074)	(0.033)	(0.029)
	t = -8.962	t = -8.483	t = -7.465	t = -8.130
	p = 0.000	p = 0.000	p = 0.000	p = 0.000
PolBal	-0.316***	-0.292***	-0.170***	-0.162***
	(0.067)	(0.068)	(0.046)	(0.049)
	t = -4.721	t = -4.297	t = -3.667	t = -3.305
	p = 0.00001	p = 0.00002	p = 0.0003	p = 0.001
FCers	-0.239***	-0.224***	-0.107***	-0.116***
	(0.037)	(0.039)	(0.025)	(0.022)
	t = -6.412	t = -5.752	t = -4.346	t = -5.349
	p = 0.000	p = 0.000	p = 0.00002	p = 0.00000
Journ	-0.646***	-0.649***	-0.217***	-0.225***
	(0.040)	(0.042)	(0.015)	(0.015)

	t = -16.097	t = -15.385	t = -14.788	t = -14.996
	p = 0.000	p = 0.000	p = 0.000	p = 0.000
Discuss	0.112***	0.115***	0.020	0.007
	(0.031)	(0.033)	(0.014)	(0.014)
	t = 3.652	t = 3.475	t = 1.388	t = 0.484
	p = 0.0003	p = 0.001	p = 0.166	p = 0.629
SubjectPartyInd	0.178**	0.194**	0.010	0.007
	(0.064)	(0.067)	(0.024)	(0.029)
	t = 2.779	t = 2.911	t = 0.436	t = 0.230
	p = 0.006	p = 0.004	p = 0.663	p = 0.818
SubjectPartyDem	0.688***	0.727***	0.051*	0.053**
	(0.051)	(0.053)	(0.025)	(0.020)
	t = 13.545	t = 13.685	t = 2.020	t = 2.663
	p = 0.000	p = 0.000	p = 0.044	p = 0.008
NatRep:Size30	0.307***	0.330***	0.119***	0.106***
	(0.091)	(0.094)	(0.028)	(0.030)
	t = 3.390	t = 3.506	t = 4.219	t = 3.575
	p = 0.001	p = 0.0005	p = 0.00003	p = 0.0004
NatRep:Size3000	0.341***	0.392***	0.167***	0.163***
	(0.091)	(0.097)	(0.031)	(0.032)
	t = 3.755	t = 4.051	t = 5.308	t = 5.164
	p = 0.0002	p = 0.0001	p = 0.00000	p = 0.00000
SMUsers:Size30	0.215*	0.197*	0.114**	0.103**
	(0.086)	(0.088)	(0.035)	(0.033)
	t = 2.507	t = 2.232	t = 3.265	t = 3.094
	p = 0.013	p = 0.026	p = 0.002	p = 0.002
SMUsers:Size3000	0.397***	0.350***	0.166***	0.155***
	(0.091)	(0.095)	(0.032)	(0.032)
	t = 4.366	t = 3.685	t = 5.166	t = 4.769
	p = 0.00002	p = 0.0003	p = 0.00000	p = 0.00001
PolBal:Size30	0.274***	0.245**	0.161**	0.154**
	(0.082)	(0.086)	(0.057)	(0.055)
	t = 3.352	t = 2.846	t = 2.813	t = 2.789
	p = 0.001	p = 0.005	p = 0.005	p = 0.006
PolBal:Size3000	0.317***	0.311***	0.184***	0.160***
	(0.088)	(0.088)	(0.042)	(0.039)
	t = 3.618	t = 3.516	t = 4.378	t = 4.059
	p = 0.0003	p = 0.0005	p = 0.00002	p = 0.00005
NatRep:Qual	0.258**	0.269**	0.144***	0.136***

	(0.085)	(0.089)	(0.030)	(0.033)
	t = 3.050	t = 3.033	t = 4.790	t = 4.139
	p = 0.003	p = 0.003	p = 0.00001	p = 0.00004
SMUsers:Qual	0.287**	0.252*	0.084*	0.070
	(0.094)	(0.102)	(0.040)	(0.044)
	t = 3.039	t = 2.464	t = 2.106	t = 1.568
	p = 0.003	p = 0.014	p = 0.036	p = 0.117
PolBal:Qual	0.037	0.021	0.125**	0.105*
	(0.092)	(0.094)	(0.045)	(0.053)
	t = 0.407	t = 0.222	t = 2.783	t = 1.963
	p = 0.685	p = 0.824	p = 0.006	p = 0.050
NatRep:Discuss	-0.041	-0.023	0.024	0.026
	(0.088)	(0.097)	(0.033)	(0.025)
	t = -0.468	t = -0.234	t = 0.739	t = 1.017
	p = 0.640	p = 0.815	p = 0.461	p = 0.310
SMUsers:Discuss	-0.029	-0.050	0.023	0.019
	(0.092)	(0.095)	(0.050)	(0.048)
	t = -0.309	t = -0.530	t = 0.454	t = 0.387
	p = 0.757	p = 0.597	p = 0.650	p = 0.700
PolBal:Discuss	-0.079	-0.062	0.047	0.050
	(0.091)	(0.094)	(0.048)	(0.045)
	t = -0.860	t = -0.656	t = 0.992	t = 1.122
	p = 0.390	p = 0.513	p = 0.322	p = 0.262
Discuss:FCers	-0.028	-0.026	0.036	0.050
	(0.039)	(0.045)	(0.029)	(0.027)
	t = -0.711	t = -0.594	t = 1.259	t = 1.901
	p = 0.477	p = 0.553	p = 0.208	p = 0.058
Discuss:Journ	-0.087*	-0.074	-0.007	0.003
	(0.040)	(0.044)	(0.023)	(0.024)
	t = -2.197	t = -1.686	t = -0.322	t = 0.131
	p = 0.029	p = 0.092	p = 0.748	p = 0.896
Coin:SubjectPartyInd	-0.014	-0.013	-0.054	-0.047
	(0.094)	(0.099)	(0.028)	(0.033)
	t = -0.146	t = -0.134	t = -1.894	t = -1.426
	p = 0.884	p = 0.894	p = 0.059	p = 0.154
Coin:SubjectPartyDem	-0.588***	-0.613***	-0.142***	-0.143***
	(0.076)	(0.079)	(0.034)	(0.027)
	t = -7.713	t = -7.756	t = -4.209	t = -5.290
	p = 0.000	p = 0.000	p = 0.00003	p = 0.00000

Algo:SubjectPartyInd	-0.058	-0.080	-0.017	-0.030
	(0.090)	(0.093)	(0.053)	(0.050)
	t = -0.645	t = -0.863	t = -0.325	t = -0.604
	p = 0.520	p = 0.389	p = 0.746	p = 0.547
Algo:SubjectPartyDem	-0.422***	-0.468***	-0.073	-0.085*
	(0.074)	(0.078)	(0.040)	(0.034)
	t = -5.685	t = -5.981	t = -1.839	t = -2.518
	p = 0.000	p = 0.000	p = 0.066	p = 0.012
Zuck:SubjectPartyInd	0.123	0.122	-0.015	-0.008
	(0.092)	(0.097)	(0.035)	(0.047)
	t = 1.345	t = 1.257	t = -0.414	t = -0.172
	p = 0.179	p = 0.209	p = 0.679	p = 0.864
Zuck:SubjectPartyDem	-0.245**	-0.260**	-0.046	-0.040
	(0.081)	(0.085)	(0.033)	(0.026)
	t = -3.022	t = -3.046	t = -1.381	t = -1.542
	p = 0.003	p = 0.003	p = 0.168	p = 0.124
NatRep:SubjectPartyInd	-0.043	-0.043	-0.020	-0.032
	(0.109)	(0.112)	(0.029)	(0.036)
	t = -0.395	t = -0.386	t = -0.696	t = -0.891
	p = 0.693	p = 0.700	p = 0.487	p = 0.374
NatRep:SubjectPartyDem	-0.652***	-0.627***	-0.133**	-0.146**
	(0.094)	(0.099)	(0.043)	(0.050)
	t = -6.913	t = -6.314	t = -3.105	t = -2.946
	p = 0.000	p = 0.000	p = 0.002	p = 0.004
SMUsers:SubjectPartyInd	-0.286*	-0.335**	-0.080	-0.097
	(0.119)	(0.123)	(0.063)	(0.065)
	t = -2.403	t = -2.735	t = -1.264	t = -1.506
	p = 0.017	p = 0.007	p = 0.207	p = 0.133
SMUsers:SubjectPartyDem	-0.741***	-0.745***	-0.146***	-0.172***
	(0.096)	(0.100)	(0.044)	(0.040)
	t = -7.746	t = -7.427	t = -3.325	t = -4.282
	p = 0.000	p = 0.000	p = 0.001	p = 0.00002
PolBal:SubjectPartyInd	-0.152	-0.173	-0.077	-0.080
	(0.106)	(0.107)	(0.054)	(0.068)
	t = -1.432	t = -1.614	t = -1.420	t = -1.182
	p = 0.153	p = 0.107	p = 0.156	p = 0.238
PolBal:SubjectPartyDem	-0.503***	-0.558***	-0.122*	-0.138*
	(0.089)	(0.091)	(0.055)	(0.058)
	t = -5.668	t = -6.111	t = -2.211	t = -2.377
	p = 0.000	p = 0.000	p = 0.028	p = 0.018

FCers:SubjectPartyInd	0.220^{***}	0.206^{***}	0.095^{**}	0.100^{**}
	(0.056)	(0.057)	(0.029)	(0.034)
	t = 3.925	t = 3.631	t = 3.249	t = 2.967
	p = 0.0001	p = 0.0003	p = 0.002	p = 0.004
FCers:SubjectPartyDem	0.352^{***}	0.333^{***}	0.149^{***}	0.141^{***}
	(0.047)	(0.049)	(0.033)	(0.033)
	t = 7.554	t = 6.730	t = 4.485	t = 4.294
	p = 0.000	p = 0.000	p = 0.00001	p = 0.00002
Journ:SubjectPartyInd	0.301^{***}	0.305^{***}	0.080^{**}	0.074^{**}
	(0.061)	(0.063)	(0.025)	(0.026)
	t = 4.935	t = 4.862	t = 3.158	t = 2.837
	p = 0.00000	p = 0.00001	p = 0.002	p = 0.005
Journ:SubjectPartyDem	0.283^{***}	0.295^{***}	0.090^{**}	0.090^{**}
	(0.050)	(0.053)	(0.031)	(0.028)
	t = 5.637	t = 5.510	t = 2.916	t = 3.264
	p = 0.00000	p = 0.00000	p = 0.004	p = 0.002
Discuss:SubjectPartyInd	0.014	0.010	0.044	0.066
	(0.048)	(0.051)	(0.030)	(0.039)
	t = 0.289	t = 0.204	t = 1.443	t = 1.686
	p = 0.773	p = 0.839	p = 0.150	p = 0.092
Discuss:SubjectPartyDem	0.100^{*}	0.081	0.055^{**}	0.060^{**}
	(0.040)	(0.043)	(0.020)	(0.021)
	t = 2.491	t = 1.863	t = 2.803	t = 2.809
	p = 0.013	p = 0.063	p = 0.006	p = 0.005
NatRep:Size30:Qual	-0.105	-0.104	-0.024	-0.014
	(0.124)	(0.131)	(0.026)	(0.044)
	t = -0.851	t = -0.793	t = -0.920	t = -0.315
	p = 0.395	p = 0.428	p = 0.358	p = 0.754
NatRep:Size3000:Qual	-0.044	-0.067	-0.032	-0.036
	(0.126)	(0.133)	(0.041)	(0.051)
	t = -0.349	t = -0.504	t = -0.785	t = -0.707
	p = 0.727	p = 0.614	p = 0.433	p = 0.480
SMUsers:Size30:Qual	0.088	0.136	0.052	0.049
	(0.116)	(0.123)	(0.055)	(0.056)
	t = 0.763	t = 1.106	t = 0.951	t = 0.886
	p = 0.446	p = 0.269	p = 0.342	p = 0.376
SMUsers:Size3000:Qual	-0.079	0.004	0.019	0.026
	(0.129)	(0.143)	(0.058)	(0.061)
	t = -0.616	t = 0.027	t = 0.321	t = 0.421

	p = 0.538	p = 0.979	p = 0.749	p = 0.674
PolBal:Size30:Qual	0.192	0.234	-0.034	-0.029
	(0.117)	(0.125)	(0.090)	(0.087)
	t = 1.641	t = 1.871	t = -0.379	t = -0.334
	p = 0.101	p = 0.062	p = 0.705	p = 0.739
PolBal:Size3000:Qual	0.160	0.190	-0.020	0.022
	(0.122)	(0.121)	(0.043)	(0.049)
	t = 1.314	t = 1.565	t = -0.470	t = 0.448
	p = 0.189	p = 0.118	p = 0.639	p = 0.655
NatRep:Size30:Discuss	0.112	0.137	-0.026	-0.005
	(0.123)	(0.131)	(0.046)	(0.047)
	t = 0.914	t = 1.042	t = -0.579	t = -0.109
	p = 0.361	p = 0.298	p = 0.563	p = 0.914
NatRep:Size3000:Discuss	0.059	0.060	-0.034	-0.024
	(0.124)	(0.135)	(0.057)	(0.057)
	t = 0.475	t = 0.445	t = -0.600	t = -0.413
	p = 0.635	p = 0.657	p = 0.549	p = 0.680
SMUsers:Size30:Discuss	-0.015	0.078	-0.007	0.020
	(0.122)	(0.126)	(0.039)	(0.043)
	t = -0.121	t = 0.616	t = -0.182	t = 0.455
	p = 0.904	p = 0.538	p = 0.856	p = 0.650
SMUsers:Size3000:Discuss	-0.096	-0.029	-0.055	-0.039
	(0.124)	(0.135)	(0.039)	(0.050)
	t = -0.776	t = -0.218	t = -1.394	t = -0.774
	p = 0.438	p = 0.828	p = 0.164	p = 0.439
PolBal:Size30:Discuss	0.054	0.062	-0.007	-0.003
	(0.117)	(0.120)	(0.043)	(0.041)
	t = 0.459	t = 0.518	t = -0.154	t = -0.083
	p = 0.646	p = 0.605	p = 0.878	p = 0.934
PolBal:Size3000:Discuss	-0.069	-0.054	-0.071	-0.035
	(0.122)	(0.126)	(0.049)	(0.045)
	t = -0.563	t = -0.432	t = -1.453	t = -0.788
	p = 0.574	p = 0.666	p = 0.147	p = 0.431
NatRep:Qual:Discuss	0.086	0.081	-0.017	-0.006
	(0.121)	(0.130)	(0.034)	(0.038)
	t = 0.711	t = 0.625	t = -0.506	t = -0.168
	p = 0.478	p = 0.533	p = 0.613	p = 0.867
SMUsers:Qual:Discuss	-0.081	-0.018	-0.022	0.004
	(0.136)	(0.147)	(0.020)	(0.029)

	t = -0.598	t = -0.120	t = -1.107	t = 0.144
	p = 0.550	p = 0.905	p = 0.269	p = 0.886
PolBal:Qual:Discuss	0.232	0.235	-0.061	-0.032
	(0.123)	(0.128)	(0.069)	(0.075)
	t = 1.890	t = 1.832	t = -0.886	t = -0.421
	p = 0.059	p = 0.067	p = 0.376	p = 0.674
NatRep:Size30:SubjectPartyInd	-0.071	-0.060	0.067	0.102
	(0.133)	(0.135)	(0.050)	(0.054)
	t = -0.536	t = -0.441	t = 1.349	t = 1.873
	p = 0.592	p = 0.659	p = 0.178	p = 0.062
NatRep:Size3000:SubjectPartyInd	-0.071	-0.127	-0.002	0.007
	(0.132)	(0.136)	(0.046)	(0.045)
	t = -0.538	t = -0.933	t = -0.054	t = 0.163
	p = 0.591	p = 0.351	p = 0.957	p = 0.871
NatRep:Size30:SubjectPartyDem	0.105	0.094	-0.020	-0.003
	(0.117)	(0.121)	(0.026)	(0.035)
	t = 0.901	t = 0.776	t = -0.788	t = -0.075
	p = 0.368	p = 0.438	p = 0.431	p = 0.941
NatRep:Size3000:SubjectPartyDem	0.128	0.080	0.027	0.032
	(0.114)	(0.122)	(0.052)	(0.058)
	t = 1.125	t = 0.660	t = 0.518	t = 0.541
	p = 0.261	p = 0.510	p = 0.605	p = 0.589
SMUsers:Size30:SubjectPartyInd	0.157	0.243	0.040	0.045
	(0.142)	(0.142)	(0.071)	(0.069)
	t = 1.100	t = 1.706	t = 0.561	t = 0.643
	p = 0.272	p = 0.088	p = 0.575	p = 0.521
SMUsers:Size3000:SubjectPartyInd	0.010	0.058	0.077	0.081
	(0.141)	(0.146)	(0.047)	(0.049)
	t = 0.068	t = 0.401	t = 1.643	t = 1.643
	p = 0.946	p = 0.689	p = 0.101	p = 0.101
SMUsers:Size30:SubjectPartyDem	0.311^{**}	0.294^{*}	-0.011	-0.006
	(0.115)	(0.120)	(0.032)	(0.035)
	t = 2.702	t = 2.446	t = -0.331	t = -0.166
	p = 0.007	p = 0.015	p = 0.741	p = 0.868
SMUsers:Size3000:SubjectPartyDem	0.097	0.093	-0.045	-0.024
	(0.115)	(0.121)	(0.042)	(0.037)
	t = 0.845	t = 0.769	t = -1.070	t = -0.635
	p = 0.398	p = 0.442	p = 0.285	p = 0.526
PolBal:Size30:SubjectPartyInd	-0.172	-0.175	-0.030	-0.039

	(0.124)	(0.128)	(0.073)	(0.088)
	t = -1.383	t = -1.369	t = -0.414	t = -0.449
	p = 0.167	p = 0.172	p = 0.679	p = 0.654
PolBal:Size3000:SubjectPartyInd	-0.289*	-0.257	-0.084*	-0.063
	(0.132)	(0.134)	(0.042)	(0.056)
	t = -2.199	t = -1.919	t = -2.004	t = -1.119
	p = 0.028	p = 0.056	p = 0.046	p = 0.263
PolBal:Size30:SubjectPartyDem	-0.072	-0.024	-0.063	-0.052
	(0.104)	(0.109)	(0.048)	(0.049)
	t = -0.690	t = -0.220	t = -1.309	t = -1.062
	p = 0.490	p = 0.826	p = 0.191	p = 0.289
PolBal:Size3000:SubjectPartyDem	-0.203	-0.157	-0.107*	-0.086
	(0.113)	(0.116)	(0.051)	(0.052)
	t = -1.802	t = -1.352	t = -2.106	t = -1.650
	p = 0.072	p = 0.177	p = 0.036	p = 0.100
NatRep:Qual:SubjectPartyInd	-0.010	0.009	-0.100	-0.076
	(0.134)	(0.136)	(0.057)	(0.064)
	t = -0.075	t = 0.068	t = -1.760	t = -1.181
	p = 0.940	p = 0.946	p = 0.079	p = 0.238
NatRep:Qual:SubjectPartyDem	0.104	0.088	-0.008	0.005
	(0.110)	(0.117)	(0.051)	(0.067)
	t = 0.943	t = 0.757	t = -0.148	t = 0.079
	p = 0.346	p = 0.449	p = 0.883	p = 0.937
SMUsers:Qual:SubjectPartyInd	0.149	0.226	0.088	0.101
	(0.147)	(0.150)	(0.081)	(0.072)
	t = 1.011	t = 1.505	t = 1.083	t = 1.408
	p = 0.313	p = 0.133	p = 0.279	p = 0.160
SMUsers:Qual:SubjectPartyDem	0.092	0.119	0.061	0.087
	(0.119)	(0.128)	(0.047)	(0.053)
	t = 0.773	t = 0.927	t = 1.297	t = 1.636
	p = 0.440	p = 0.354	p = 0.195	p = 0.102
PolBal:Qual:SubjectPartyInd	0.202	0.215	0.046	0.054
	(0.137)	(0.140)	(0.067)	(0.081)
	t = 1.471	t = 1.544	t = 0.692	t = 0.673
	p = 0.142	p = 0.123	p = 0.489	p = 0.502
PolBal:Qual:SubjectPartyDem	0.225	0.252*	-0.018	-0.001
	(0.116)	(0.118)	(0.050)	(0.060)
	t = 1.945	t = 2.134	t = -0.360	t = -0.011
	p = 0.052	p = 0.033	p = 0.719	p = 0.992

NatRep:Discuss:SubjectPartyInd	-0.007	-0.007	-0.087	-0.090
	(0.137)	(0.147)	(0.051)	(0.051)
	t = -0.052	t = -0.047	t = -1.703	t = -1.758
	p = 0.959	p = 0.963	p = 0.089	p = 0.079
NatRep:Discuss:SubjectPartyDem	0.177	0.167	-0.064	-0.034
	(0.116)	(0.126)	(0.054)	(0.062)
	t = 1.524	t = 1.331	t = -1.185	t = -0.547
	p = 0.128	p = 0.184	p = 0.237	p = 0.585
SMUsers:Discuss:SubjectPartyInd	0.255	0.353*	0.003	0.001
	(0.145)	(0.151)	(0.102)	(0.100)
	t = 1.756	t = 2.336	t = 0.032	t = 0.012
	p = 0.080	p = 0.020	p = 0.975	p = 0.991
SMUsers:Discuss:SubjectPartyDem	0.123	0.155	-0.009	0.008
	(0.118)	(0.122)	(0.059)	(0.056)
	t = 1.047	t = 1.268	t = -0.144	t = 0.143
	p = 0.296	p = 0.205	p = 0.886	p = 0.887
PolBal:Discuss:SubjectPartyInd	0.040	0.016	-0.075	-0.071
	(0.141)	(0.146)	(0.078)	(0.080)
	t = 0.282	t = 0.109	t = -0.961	t = -0.898
	p = 0.779	p = 0.914	p = 0.337	p = 0.369
PolBal:Discuss:SubjectPartyDem	-0.190	-0.187	-0.131*	-0.132
	(0.121)	(0.126)	(0.064)	(0.074)
	t = -1.576	t = -1.486	t = -2.048	t = -1.789
	p = 0.116	p = 0.138	p = 0.041	p = 0.074
Discuss:FCers:SubjectPartyInd	-0.00003	0.007	-0.056	-0.060
	(0.062)	(0.066)	(0.042)	(0.047)
	t = -0.001	t = 0.107	t = -1.330	t = -1.282
	p = 1.000	p = 0.915	p = 0.184	p = 0.200
Discuss:FCers:SubjectPartyDem	0.005	-0.001	-0.044	-0.056
	(0.052)	(0.057)	(0.036)	(0.049)
	t = 0.095	t = -0.012	t = -1.235	t = -1.128
	p = 0.924	p = 0.991	p = 0.217	p = 0.260
Discuss:Journ:SubjectPartyInd	-0.012	-0.006	-0.006	-0.008
	(0.062)	(0.066)	(0.044)	(0.046)
	t = -0.186	t = -0.087	t = -0.136	t = -0.169
	p = 0.853	p = 0.931	p = 0.892	p = 0.866
Discuss:Journ:SubjectPartyDem	0.029	0.035	-0.009	-0.007
	(0.052)	(0.057)	(0.021)	(0.029)
	t = 0.555	t = 0.606	t = -0.435	t = -0.251
	p = 0.580	p = 0.545	p = 0.664	p = 0.802

NatRep:Size30:Qual:Discuss	-0.122	-0.150	0.008	-0.008
	(0.171)	(0.180)	(0.060)	(0.069)
	t = -0.718	t = -0.832	t = 0.137	t = -0.112
	p = 0.473	p = 0.406	p = 0.891	p = 0.911
NatRep:Size3000:Qual:Discuss	-0.127	-0.148	-0.006	-0.012
	(0.180)	(0.194)	(0.067)	(0.080)
	t = -0.707	t = -0.761	t = -0.094	t = -0.151
	p = 0.480	p = 0.447	p = 0.925	p = 0.881
SMUsers:Size30:Qual:Discuss	0.132	0.009	0.013	-0.007
	(0.175)	(0.187)	(0.049)	(0.058)
	t = 0.756	t = 0.048	t = 0.265	t = -0.119
	p = 0.450	p = 0.962	p = 0.791	p = 0.906
SMUsers:Size3000:Qual:Discuss	0.155	0.052	0.096	0.074
	(0.182)	(0.196)	(0.052)	(0.057)
	t = 0.852	t = 0.268	t = 1.849	t = 1.314
	p = 0.395	p = 0.789	p = 0.065	p = 0.189
PolBal:Size30:Qual:Discuss	-0.291	-0.324	0.064	0.046
	(0.159)	(0.175)	(0.092)	(0.095)
	t = -1.828	t = -1.855	t = 0.690	t = 0.490
	p = 0.068	p = 0.064	p = 0.491	p = 0.624
PolBal:Size3000:Qual:Discuss	-0.122	-0.176	0.071	0.014
	(0.182)	(0.187)	(0.074)	(0.080)
	t = -0.673	t = -0.941	t = 0.968	t = 0.178
	p = 0.502	p = 0.347	p = 0.333	p = 0.859
NatRep:Size30:Qual:SubjectPartyInd	0.076	0.008	0.016	-0.012
	(0.199)	(0.204)	(0.079)	(0.099)
	t = 0.383	t = 0.038	t = 0.198	t = -0.122
	p = 0.702	p = 0.970	p = 0.843	p = 0.903
NatRep:Size3000:Qual:SubjectPartyInd	-0.048	-0.035	0.077	0.066
	(0.190)	(0.193)	(0.085)	(0.089)
	t = -0.254	t = -0.181	t = 0.907	t = 0.738
	p = 0.800	p = 0.857	p = 0.365	p = 0.461
NatRep:Size30:Qual:SubjectPartyDem	0.102	0.125	0.134*	0.125
	(0.162)	(0.172)	(0.061)	(0.092)
	t = 0.628	t = 0.723	t = 2.187	t = 1.359
	p = 0.531	p = 0.470	p = 0.029	p = 0.175
NatRep:Size3000:Qual:SubjectPartyDem	0.093	0.108	0.074	0.076
	(0.160)	(0.169)	(0.073)	(0.089)
	t = 0.581	t = 0.637	t = 1.022	t = 0.855

	p = 0.562	p = 0.525	p = 0.308	p = 0.393
SMUsers:Size30:Qual:SubjectPartyInd	-0.345	-0.451*	-0.044	-0.056
	(0.191)	(0.194)	(0.124)	(0.114)
	t = -1.804	t = -2.323	t = -0.350	t = -0.491
	p = 0.072	p = 0.021	p = 0.727	p = 0.624
SMUsers:Size3000:Qual:SubjectPartyInd	-0.027	-0.123	-0.081	-0.071
	(0.197)	(0.209)	(0.095)	(0.095)
	t = -0.138	t = -0.588	t = -0.844	t = -0.742
	p = 0.891	p = 0.557	p = 0.399	p = 0.458
SMUsers:Size30:Qual:SubjectPartyDem	-0.160	-0.160	-0.008	-0.011
	(0.149)	(0.159)	(0.046)	(0.053)
	t = -1.071	t = -1.005	t = -0.167	t = -0.217
	p = 0.284	p = 0.315	p = 0.867	p = 0.828
SMUsers:Size3000:Qual:SubjectPartyDem	0.108	0.065	0.049	0.020
	(0.161)	(0.176)	(0.063)	(0.065)
	t = 0.671	t = 0.368	t = 0.774	t = 0.310
	p = 0.503	p = 0.713	p = 0.440	p = 0.757
PolBal:Size30:Qual:SubjectPartyInd	-0.015	-0.027	-0.010	0.003
	(0.176)	(0.188)	(0.112)	(0.124)
	t = -0.086	t = -0.142	t = -0.093	t = 0.023
	p = 0.932	p = 0.888	p = 0.926	p = 0.982
PolBal:Size3000:Qual:SubjectPartyInd	-0.048	-0.093	0.029	0.007
	(0.188)	(0.191)	(0.064)	(0.072)
	t = -0.254	t = -0.486	t = 0.454	t = 0.104
	p = 0.800	p = 0.628	p = 0.650	p = 0.918
PolBal:Size30:Qual:SubjectPartyDem	-0.170	-0.222	0.102	0.086
	(0.148)	(0.155)	(0.087)	(0.084)
	t = -1.149	t = -1.426	t = 1.170	t = 1.020
	p = 0.251	p = 0.154	p = 0.243	p = 0.308
PolBal:Size3000:Qual:SubjectPartyDem	0.046	-0.028	0.096	0.051
	(0.158)	(0.163)	(0.057)	(0.074)
	t = 0.294	t = -0.173	t = 1.670	t = 0.694
	p = 0.769	p = 0.863	p = 0.096	p = 0.488
NatRep:Size30:Discuss:SubjectPartyInd	-0.029	-0.075	-0.034	-0.082
	(0.184)	(0.191)	(0.070)	(0.078)
	t = -0.160	t = -0.392	t = -0.482	t = -1.054
	p = 0.873	p = 0.696	p = 0.630	p = 0.293
NatRep:Size3000:Discuss:SubjectPartyInd	-0.052	-0.012	0.013	0.003
	(0.185)	(0.196)	(0.086)	(0.076)

	t = -0.282	t = -0.060	t = 0.152	t = 0.037
	p = 0.778	p = 0.953	p = 0.880	p = 0.971
NatRep:Size30:Discuss:SubjectPartyDem	-0.214	-0.276	0.130**	0.091
	(0.160)	(0.170)	(0.048)	(0.069)
	t = -1.341	t = -1.629	t = 2.725	t = 1.317
	p = 0.181	p = 0.104	p = 0.007	p = 0.188
NatRep:Size3000:Discuss:SubjectPartyDem	-0.257	-0.252	-0.018	-0.048
	(0.158)	(0.169)	(0.073)	(0.081)
	t = -1.626	t = -1.485	t = -0.252	t = -0.593
	p = 0.104	p = 0.138	p = 0.802	p = 0.554
SMUsers:Size30:Discuss:SubjectPartyInd	-0.202	-0.445*	-0.096	-0.102
	(0.203)	(0.208)	(0.089)	(0.092)
	t = -0.995	t = -2.141	t = -1.082	t = -1.110
	p = 0.320	p = 0.033	p = 0.280	p = 0.267
SMUsers:Size3000:Discuss:SubjectPartyInd	-0.177	-0.278	-0.107	-0.123
	(0.191)	(0.203)	(0.121)	(0.116)
	t = -0.930	t = -1.373	t = -0.879	t = -1.065
	p = 0.353	p = 0.170	p = 0.380	p = 0.287
SMUsers:Size30:Discuss:SubjectPartyDem	-0.194	-0.262	0.016	0.003
	(0.160)	(0.165)	(0.055)	(0.064)
	t = -1.215	t = -1.589	t = 0.291	t = 0.045
	p = 0.225	p = 0.113	p = 0.772	p = 0.964
SMUsers:Size3000:Discuss:SubjectPartyDem	0.055	0.032	0.033	0.017
	(0.158)	(0.169)	(0.062)	(0.060)
	t = 0.351	t = 0.192	t = 0.529	t = 0.275
	p = 0.726	p = 0.848	p = 0.597	p = 0.784
PolBal:Size30:Discuss:SubjectPartyInd	0.045	0.022	-0.033	-0.047
	(0.191)	(0.197)	(0.123)	(0.126)
	t = 0.236	t = 0.110	t = -0.270	t = -0.369
	p = 0.814	p = 0.913	p = 0.788	p = 0.712
PolBal:Size3000:Discuss:SubjectPartyInd	0.194	0.166	0.177*	0.127
	(0.185)	(0.193)	(0.086)	(0.089)
	t = 1.052	t = 0.860	t = 2.049	t = 1.429
	p = 0.293	p = 0.390	p = 0.041	p = 0.154
PolBal:Size30:Discuss:SubjectPartyDem	0.117	0.058	0.070	0.072
	(0.156)	(0.163)	(0.057)	(0.069)
	t = 0.751	t = 0.354	t = 1.216	t = 1.044
	p = 0.453	p = 0.724	p = 0.225	p = 0.297
PolBal:Size3000:Discuss:SubjectPartyDem	0.419**	0.354*	0.221***	0.187**

	(0.161)	(0.170)	(0.051)	(0.060)
	t = 2.594	t = 2.087	t = 4.329	t = 3.116
	p = 0.010	p = 0.037	p = 0.00002	p = 0.002
NatRep:Qual:Discuss:SubjectPartyInd	-0.025	-0.019	0.146*	0.138*
	(0.186)	(0.201)	(0.060)	(0.067)
	t = -0.137	t = -0.096	t = 2.427	t = 2.053
	p = 0.892	p = 0.924	p = 0.016	p = 0.041
NatRep:Qual:Discuss:SubjectPartyDem	-0.302	-0.276	0.091	0.067
	(0.156)	(0.170)	(0.056)	(0.075)
	t = -1.937	t = -1.620	t = 1.628	t = 0.887
	p = 0.053	p = 0.106	p = 0.104	p = 0.376
SMUsers:Qual:Discuss:SubjectPartyInd	-0.299	-0.453*	-0.022	-0.062
	(0.214)	(0.226)	(0.079)	(0.079)
	t = -1.401	t = -2.000	t = -0.285	t = -0.781
	p = 0.162	p = 0.046	p = 0.776	p = 0.436
SMUsers:Qual:Discuss:SubjectPartyDem	0.106	0.074	-0.032	-0.046
	(0.174)	(0.186)	(0.056)	(0.051)
	t = 0.608	t = 0.400	t = -0.578	t = -0.896
	p = 0.544	p = 0.690	p = 0.564	p = 0.371
PolBal:Qual:Discuss:SubjectPartyInd	-0.474*	-0.447*	0.037	-0.002
	(0.186)	(0.192)	(0.136)	(0.136)
	t = -2.544	t = -2.328	t = 0.270	t = -0.013
	p = 0.011	p = 0.020	p = 0.788	p = 0.990
PolBal:Qual:Discuss:SubjectPartyDem	-0.048	-0.019	0.135	0.109
	(0.158)	(0.164)	(0.093)	(0.117)
	t = -0.305	t = -0.113	t = 1.455	t = 0.929
	p = 0.761	p = 0.910	p = 0.146	p = 0.353
NatRep:Size30:Qual:Discuss:SubjectPartyInd	-0.127	-0.031	0.002	0.037
	(0.272)	(0.291)	(0.086)	(0.103)
	t = -0.468	t = -0.105	t = 0.025	t = 0.357
	p = 0.640	p = 0.917	p = 0.980	p = 0.722
NatRep:Size3000:Qual:Discuss:SubjectPartyInd	0.185	0.130	-0.054	-0.085
	(0.263)	(0.277)	(0.108)	(0.108)
	t = 0.702	t = 0.471	t = -0.505	t = -0.783
	p = 0.483	p = 0.638	p = 0.614	p = 0.434
NatRep:Size30:Qual:Discuss:SubjectPartyDem	0.408	0.414	-0.167	-0.144
	(0.223)	(0.241)	(0.094)	(0.116)
	t = 1.827	t = 1.718	t = -1.767	t = -1.246
	p = 0.068	p = 0.086	p = 0.078	p = 0.213

NatRep:Size3000:Qual:Discuss:SubjectPartyDem	0.304	0.293	-0.067	-0.056
	(0.231)	(0.249)	(0.105)	(0.125)
	t = 1.317	t = 1.176	t = -0.641	t = -0.447
	p = 0.188	p = 0.240	p = 0.522	p = 0.655
SMUsers:Size30:Qual:Discuss:SubjectPartyInd	0.488	0.757*	0.078	0.115
	(0.281)	(0.296)	(0.123)	(0.128)
	t = 1.735	t = 2.557	t = 0.634	t = 0.902
	p = 0.083	p = 0.011	p = 0.527	p = 0.368
SMUsers:Size3000:Qual:Discuss:SubjectPartyInd	0.199	0.404	0.003	0.036
	(0.279)	(0.300)	(0.126)	(0.103)
	t = 0.714	t = 1.343	t = 0.025	t = 0.348
	p = 0.476	p = 0.180	p = 0.980	p = 0.728
SMUsers:Size30:Qual:Discuss:SubjectPartyDem	-0.190	-0.119	-0.012	-0.013
	(0.228)	(0.240)	(0.091)	(0.091)
	t = -0.833	t = -0.494	t = -0.130	t = -0.140
	p = 0.405	p = 0.621	p = 0.897	p = 0.889
SMUsers:Size3000:Qual:Discuss:SubjectPartyDem	-0.283	-0.263	-0.108	-0.115
	(0.232)	(0.247)	(0.102)	(0.114)
	t = -1.218	t = -1.066	t = -1.057	t = -1.010
	p = 0.224	p = 0.287	p = 0.291	p = 0.313
PolBal:Size30:Qual:Discuss:SubjectPartyInd	0.342	0.365	0.012	0.040
	(0.246)	(0.264)	(0.191)	(0.202)
	t = 1.390	t = 1.382	t = 0.063	t = 0.197
	p = 0.165	p = 0.167	p = 0.951	p = 0.844
PolBal:Size3000:Qual:Discuss:SubjectPartyInd	0.269	0.322	-0.203	-0.136
	(0.270)	(0.278)	(0.157)	(0.164)
	t = 0.995	t = 1.158	t = -1.294	t = -0.826
	p = 0.320	p = 0.247	p = 0.196	p = 0.409
PolBal:Size30:Qual:Discuss:SubjectPartyDem	0.137	0.204	-0.167	-0.148
	(0.208)	(0.223)	(0.129)	(0.155)
	t = 0.662	t = 0.917	t = -1.290	t = -0.958
	p = 0.509	p = 0.360	p = 0.198	p = 0.338
PolBal:Size3000:Qual:Discuss:SubjectPartyDem	-0.257	-0.156	-0.218*	-0.141
	(0.231)	(0.244)	(0.100)	(0.127)
	t = -1.111	t = -0.640	t = -2.173	t = -1.114
	p = 0.267	p = 0.523	p = 0.030	p = 0.266
Constant	3.835***	3.812***	0.582***	0.588***
	(0.041)	(0.043)	(0.017)	(0.016)
	t = 92.775	t = 88.523	t = 34.000	t = 37.462
	p = 0.000	p = 0.000	p = 0.000	p = 0.000

Note: *p<.05, **p<.01, ***p<.001

OLS predicting legitimacy or choice of juries by multiple jury categories, with partisanship moderator. Baseline levels are domain expert jury, size of 3, no qualifications or discussion; and Republican party.

4. Supplemental Main Results - Collapsed Jury Type Analyses.

As pre-registered, in addition to conducting analyses with dummy variables for each of our nine jury types, we also analyzed models that collapse the nine possible jury compositions into three groups: non-jury, layperson, and expert. Non-jury includes coin flip, head of the social media company (e.g., Mark Zuckerberg of Facebook), or computer algorithm. Layperson includes nationally representative, politically balanced, or users of the social media platform. Expert includes professional fact-checkers, professional journalists, or domain experts.

For each individual jury, we use linear regression to predict average perceived legitimacy rating by a dummy variable for if the jury was a non-jury, a dummy variable for if the jury was a layperson jury, and all interactions between layperson dummy, size (30, 3,000), qualifications, and discussion, with cluster robust standard errors by participant. This model specifies expert juries (without discussion) as the baseline level (as this is the current standard of care for major social media companies like Meta), and aggregates across specific juries at the non-jury, layperson, and expert levels. We specify baseline jury features as a size of 3, no qualifications (for laypeople), and no discussion.

We additionally conduct similar analyses at the jury pair level, using a linear probability model to predict the choice proportion of juries again by non-jury and layperson dummies and all interactions between the layperson dummy and jury features, with two-way cluster robust standard errors by participant and jury pair. Expert juries (without discussion) are again our baseline level.

Legitimacy of Collapsed Jury Types.

We find that participants perceived experts as the most legitimate baseline (i.e., size of 3, no discussion) jury category (intercept=4.007, SE=0.021, t=191.954, p<.001; see SI Figure S2; SI Table S1[1]). Participants gave significantly lower legitimacy ratings to baseline layperson juries (b=-0.603, SE=0.027, t=-22.138, p<.001) and non-juries (b=-1.165, SE=0.024, t=-48.606, p<.001).

Main text Figure 3 shows the effects of varying single attributes. Allowing jurors to deliberate and discuss their evaluations with one another increased the perceived legitimacy of both expert juries (b=0.129, SE=0.012, t=10.863, p<.001), and layperson juries (no evidence of significant interaction with layperson dummy: b=0.014, SE=0.028, t=0.515, p=.607).

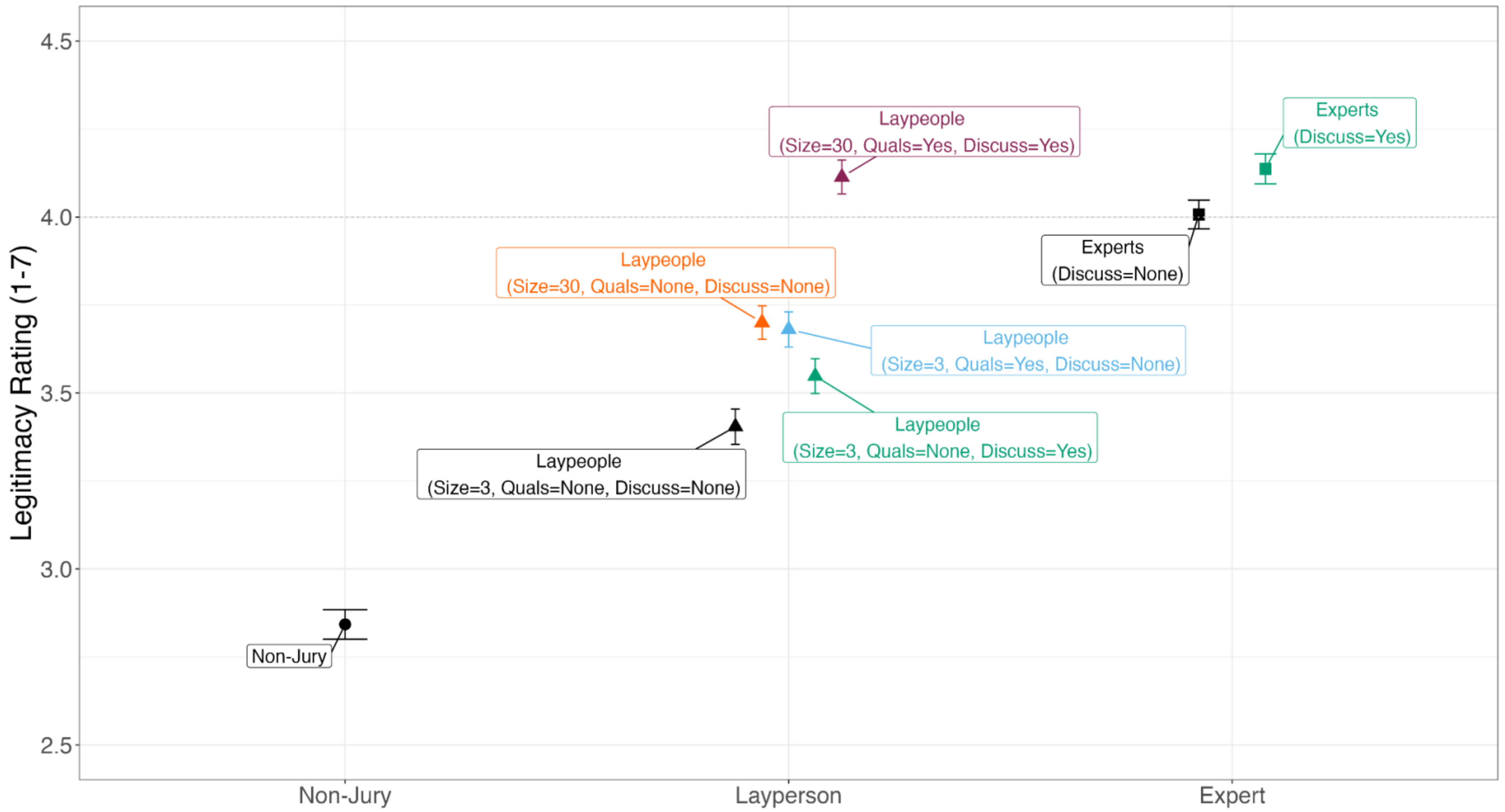

Figure S2. Category-level legitimacy ratings. The average legitimacy ratings for non-jury, layperson, and expert juries, with different size, qualification, and discussion features. Estimates reflect sample-level averages. Error bars reflect 95% confidence intervals. See SI Figure S3 for corresponding choice outcome variable plot.

For lay juries, increasing the size or requiring minimal news knowledge and reasoning ability qualifications increased perceived legitimacy. Increasing the size 3 to 30 significantly increased legitimacy (b=0.296, SE=0.027, t=10.851, p<.001), while further increasing from 30 to 3,000 had no additional benefit (b=0.296 vs b=0.323; coefficients compared via linear hypothesis test: p=.328). Requiring minimal news knowledge and reasoning ability qualifications also had an effect of similar magnitude (b=0.276, SE=0.027, t=10.313, p<.001).

We did not observe any interactions between additional jury features on layperson legitimacy perceptions (ps>.446) – rather, the legitimacy benefits of increasing size, adding qualifications, and allowing for jury discussion appear additive. Indeed, we find that layperson juries with maximal feasible features (size 30, qualifications, discussion) exhibit similar overall legitimacy perceptions as expert juries with discussion (Figure S2).

Choice of Collapsed Jury Types in Paired Analyses.

Our choice results are qualitatively similar to those predicting individual jury legitimacy. Expert juries were the most chosen baseline jury (intercept=0.544, SE=0.006, t=84.128, p<.001; SI Table S1[3]), significantly more so than baseline layperson (b=-0.216, SE=0.011, t=-18.830, p<.001) or non-jury (b=-0.342, SE=0.010, t=-35.258, p<.001). Increasing layperson jury size to 30 (b=0.124, SE=0.012, t=10.799, p<.001) or 3,000 (b=0.158, SE=0.012, t=13.305, p<.001) and adding minimum layperson qualifications (b=0.129, SE=0.008, t=16.342, p<.001) increased the choice probability of layperson juries relative to the expert baseline. Allowing discussion also increased jury choice proportion similarly for expert (b=0.053, SE=0.006, t=9.282, p<.001) and layperson (intercept b=0.0001, SE=0.012, t=0.008, p=.994) juries. Again, the benefits of individual jury features appeared largely additive when combining layperson panel characteristics (ps>.144). Here, layperson juries with maximal feasible features were actually chosen at higher rates than even expert juries with discussion (z-test of coefficients: z=4.92, p<.001; see SI Figure S3).

5. Additional Supplemental Figures.

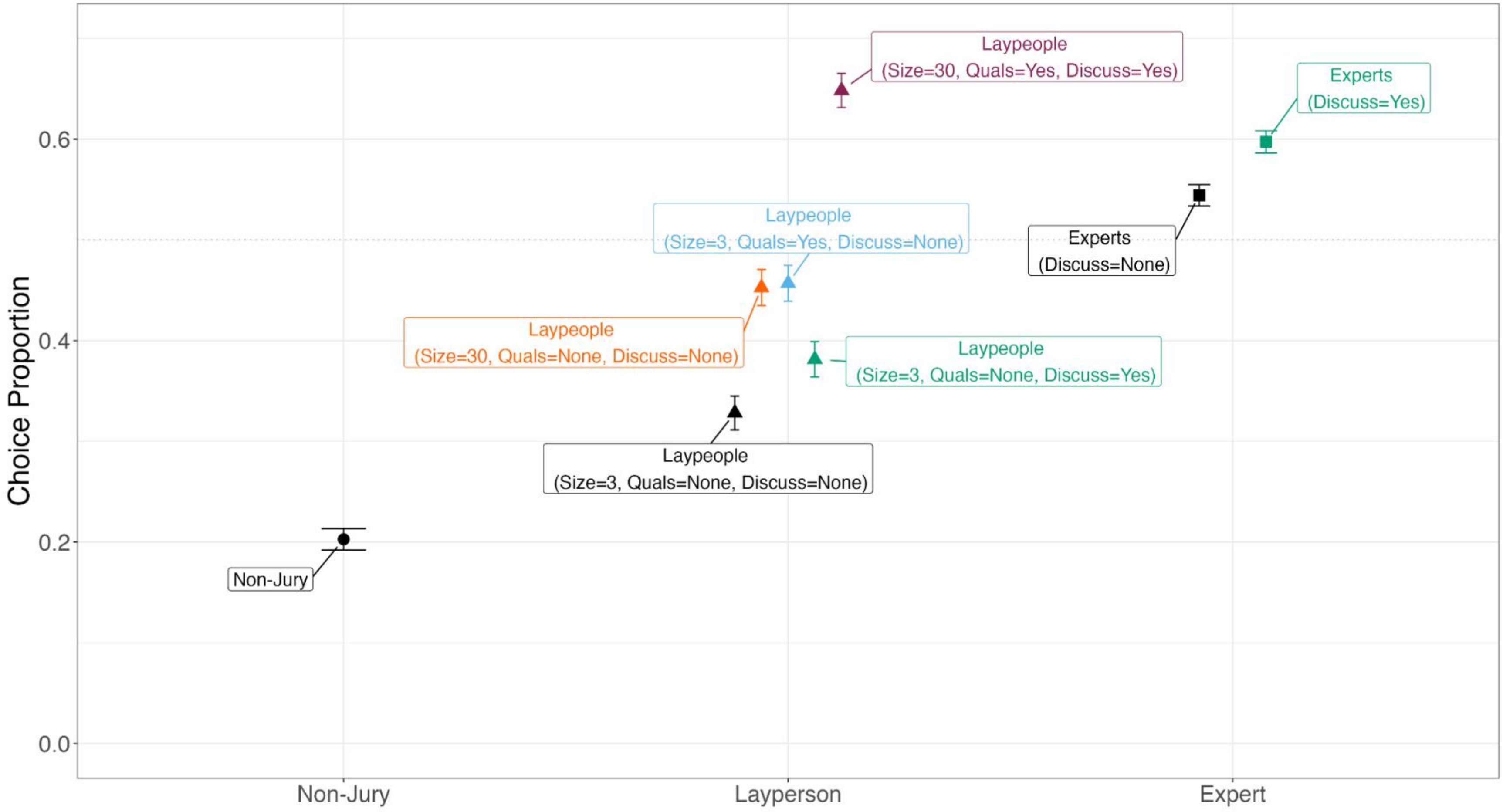

Figure S3. Category-level choice proportions. The jury choice probability for non-jury, layperson, and expert juries, with different size, qualification, and discussion features. Estimates reflect sample-level averages. Error bars reflect 95% confidence intervals.

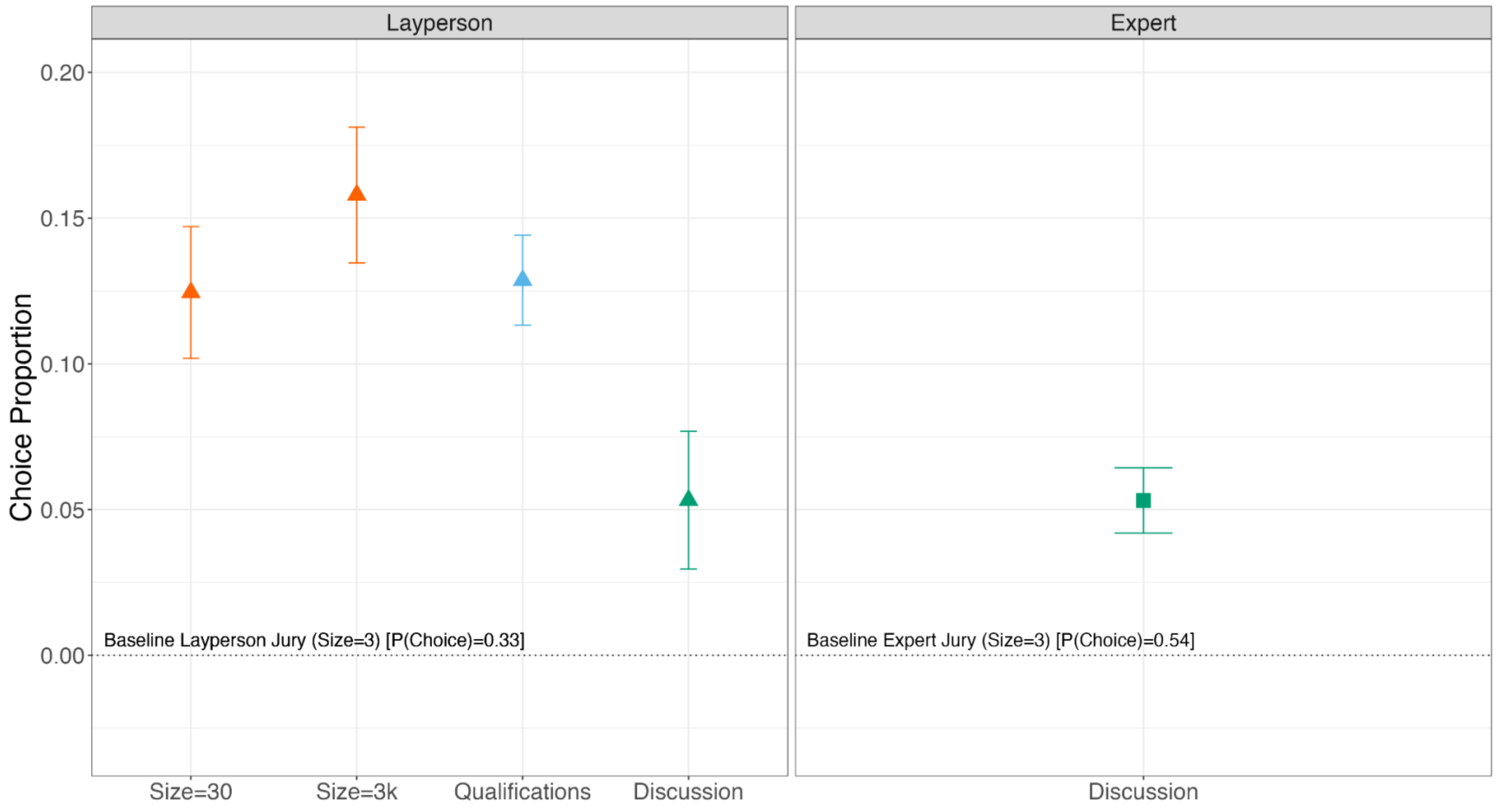

Figure S4. Category-level effects of jury features on jury choice. (Left) Relative to the baseline layperson jury with a size of 3 members (average legitimacy=3.40), the effects of individually increasing jury size to 30, increasing jury size to 3,000, adding minimum qualifications, or allowing discussion among jury members. (Right) Relative to the baseline expert jury (average legitimacy=4.01), the effect of allowing discussion among jury members. Estimates reflect sample-level effects. Error bars reflect 95% confidence intervals.

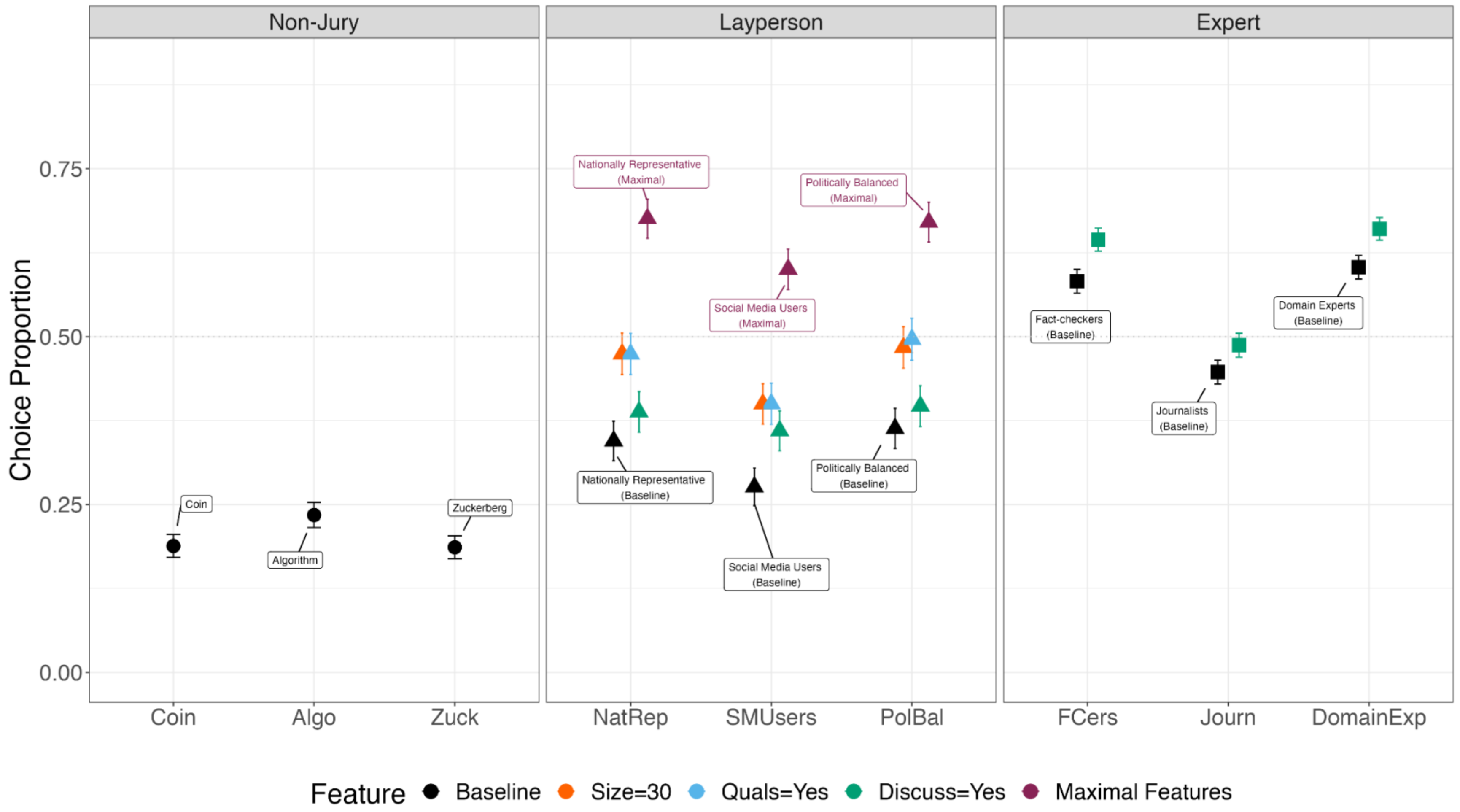

Figure S5. Jury-level choice proportions. The jury choice probability for all nine juries, with different size, qualification, and discussion features. Estimates reflect sample-level averages. Error bars reflect 95% confidence intervals.

(a)

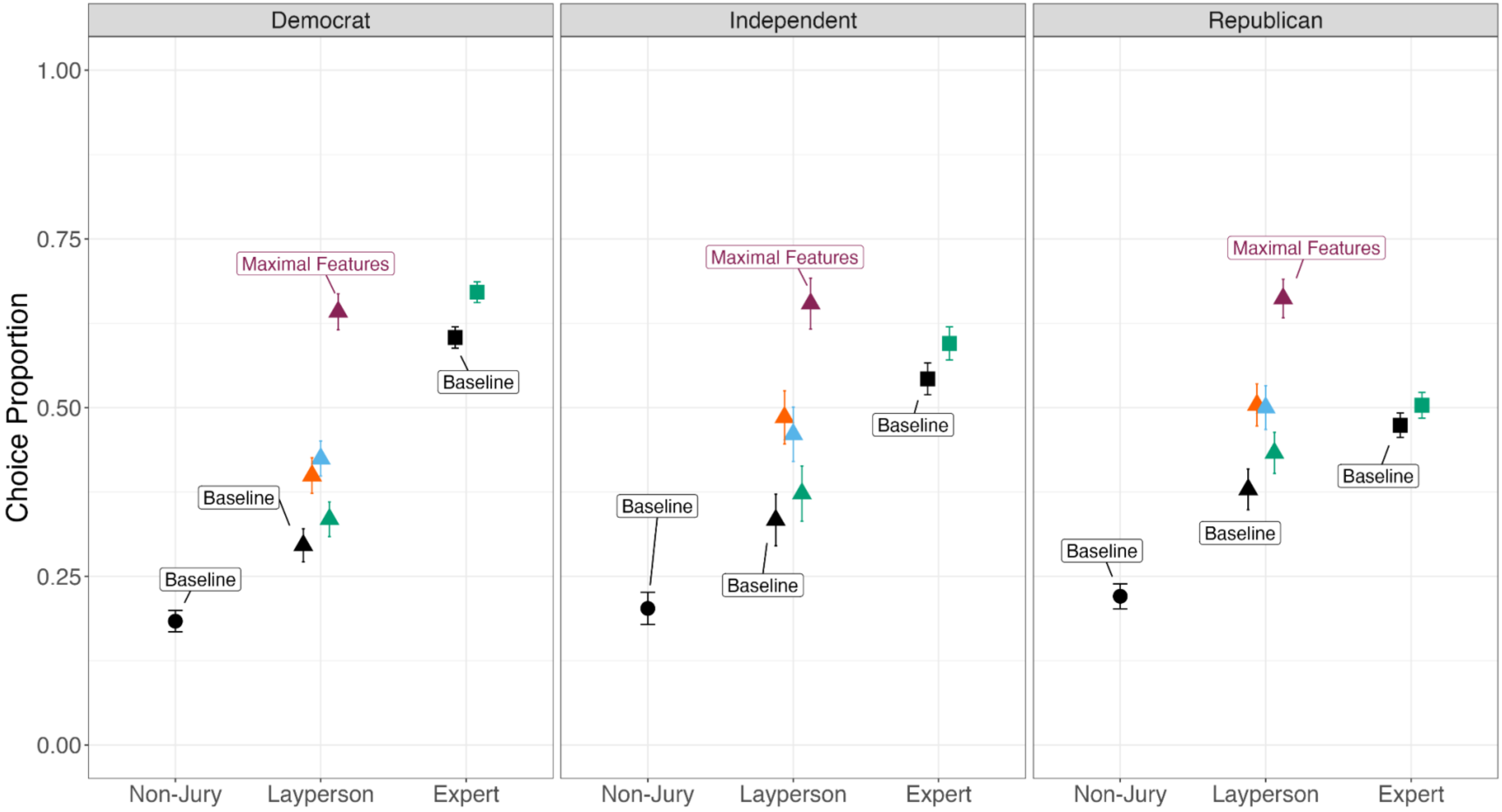
Democrat
Independent
Republican
Choice Proportion
1.00
0.75
0.50
0.25
0.00
Maximal Features
Baseline
Non-Jury
Layperson
Expert

(b)

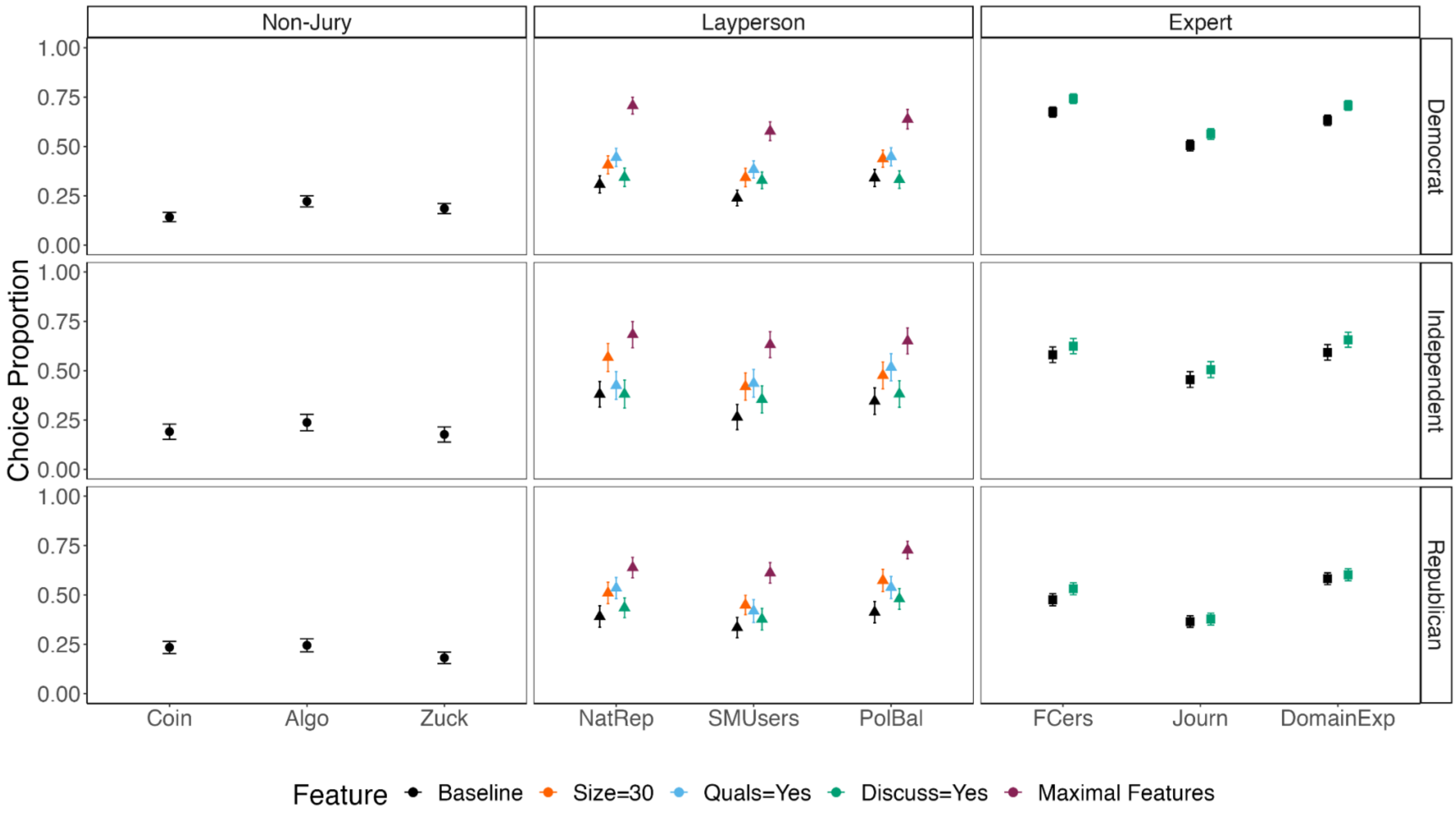

Figure S6. Jury choice proportions by partisanship. (a) The jury choice probability for non-jury, layperson, and expert juries, with different size, qualification, and discussion features, by partisanship. (b) The jury choice probability for all nine juries, with different size, qualification, and discussion features, by partisanship. Estimates reflect sample-level averages. Error bars reflect 95% confidence intervals.

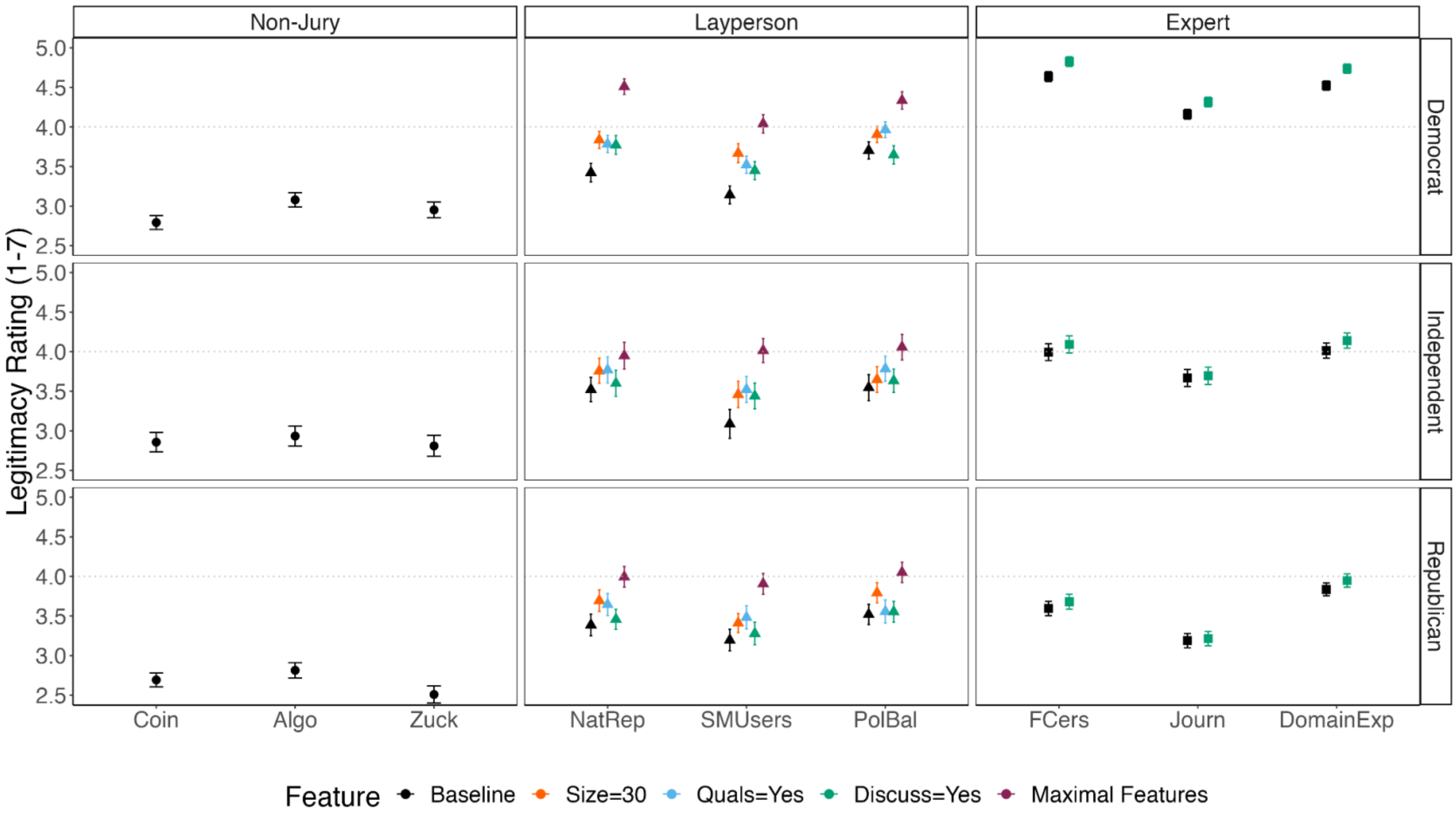

Figure S7. Legitimacy ratings by partisanship. All nine jury composition types. Estimates reflect sample-level averages (see SI tables for population-weighted estimates). Error bars reflect 95% confidence intervals. Absolute legitimacy ratings should be interpreted as those under our objection precondition wherein participants were told to suppose they disagreed with a decision from the content moderation jury. See SI Figure S6b above for corresponding choice outcome variable plot.

6. Secondary Analyses – Attention Filter.

Here we conduct robustness analyses on our primary models, filtering for only participants who correctly answered a pre-treatment instructional attention check (*N*=1,741 participants answered pre-treatment attention check correctly, while 1,259 participants answered incorrectly and are excluded from the analyses in this section; see Q2 in survey codebook, adapted from (1)).

Table S5. *Legitimacy rating or jury choice predicted by category-level jury types and features, filtering for attention. Columns (2) and (4) show results with analytic weighting by population-weights.*

	Dependent variable:			
	Legitimacy (1-7) Sample	Legitimacy (1-7) Population	Choice Sample	Choice Population
	(1)	(2)	(3)	(4)
NonJury	-1.380***	-1.368***	-0.376***	-0.373***
	(0.032)	(0.034)	(0.011)	(0.011)
	t = -42.650	t = -40.345	t = -32.999	t = -32.719
	p = 0.000	p = 0.000	p = 0.000	p = 0.000
Layperson	-0.759***	-0.755***	-0.257***	-0.249***
	(0.037)	(0.038)	(0.009)	(0.009)
	t = -20.559	t = -19.852	t = -27.805	t = -28.796
	p = 0.000	p = 0.000	p = 0.000	p = 0.000
Discuss	0.159***	0.166***	0.053***	0.056***
	(0.016)	(0.018)	(0.008)	(0.010)
	t = 9.769	t = 9.411	t = 6.309	t = 5.558
	p = 0.000	p = 0.000	p = 0.000	p = 0.00000

Layperson:Size30	0.381^{***}	0.385^{***}	0.156^{***}	0.146^{***}
	(0.037)	(0.037)	(0.015)	(0.015)
	t = 10.412	t = 10.316	t = 10.614	t = 9.965
	p = 0.000	p = 0.000	p = 0.000	p = 0.000
Layperson:Size3000	0.460^{***}	0.457^{***}	0.203^{***}	0.188^{***}
	(0.038)	(0.039)	(0.022)	(0.022)
	t = 12.086	t = 11.751	t = 9.145	t = 8.389
	p = 0.000	p = 0.000	p = 0.000	p = 0.000
Layperson:Qual	0.352^{***}	0.343^{***}	0.158^{***}	0.148^{***}
	(0.036)	(0.038)	(0.010)	(0.012)
	t = 9.730	t = 8.958	t = 15.803	t = 12.616
	p = 0.000	p = 0.000	p = 0.000	p = 0.000
Layperson:Discuss	0.024	0.034	0.013	0.009
	(0.037)	(0.038)	(0.011)	(0.010)
	t = 0.666	t = 0.881	t = 1.200	t = 0.877
	p = 0.506	p = 0.379	p = 0.231	p = 0.381
Layperson:Size30:Qual	-0.004	0.003	0.011	0.013
	(0.049)	(0.053)	(0.017)	(0.021)
	t = -0.081	t = 0.066	t = 0.655	t = 0.623
	p = 0.936	p = 0.948	p = 0.513	p = 0.534
Layperson:Size3000:Qual	-0.007	-0.004	0.002	0.015
	(0.052)	(0.055)	(0.025)	(0.027)
	t = -0.132	t = -0.076	t = 0.072	t = 0.544
	p = 0.895	p = 0.940	p = 0.943	p = 0.587
Layperson:Size30:Discuss	0.010	-0.0004	-0.002	0.005
	(0.050)	(0.052)	(0.019)	(0.021)
	t = 0.197	t = -0.008	t = -0.118	t = 0.216
	p = 0.845	p = 0.994	p = 0.907	p = 0.830
Layperson:Size3000:Discuss	-0.014	-0.015	-0.025	-0.017
	(0.049)	(0.052)	(0.028)	(0.031)
	t = -0.278	t = -0.288	t = -0.917	t = -0.568

	p = 0.782	p = 0.773	p = 0.360	p = 0.571
Layperson:Qual:Discuss	-0.048	-0.038	-0.022	-0.014
	(0.051)	(0.054)	(0.017)	(0.017)
	t = -0.954	t = -0.706	t = -1.310	t = -0.814
	p = 0.341	p = 0.481	p = 0.191	p = 0.416
Layperson:Size30:Qual:Discuss	0.029	0.026	0.001	0.004
	(0.069)	(0.075)	(0.025)	(0.027)
	t = 0.414	t = 0.346	t = 0.055	t = 0.154
	p = 0.679	p = 0.729	p = 0.957	p = 0.878
Layperson:Size3000:Qual:Discuss	-0.013	-0.012	0.003	-0.010
	(0.074)	(0.078)	(0.035)	(0.034)
	t = -0.172	t = -0.151	t = 0.085	t = -0.285
	p = 0.864	p = 0.880	p = 0.932	p = 0.776
Constant	3.949***	3.944***	0.549***	0.548***
	(0.029)	(0.031)	(0.008)	(0.007)
	t = 134.504	t = 128.288	t = 73.147	t = 74.806
	p = 0.000	p = 0.000	p = 0.000	p = 0.000

Note: *p<.05; **p<.01; ***p<.001

OLS predicting legitimacy or choice of juries by non-jury, layperson, and expert categories. Baseline levels are expert jury, size of 3, no qualifications or discussion.

Table S6. *Legitimacy rating or jury choice predicted by jury-specific levels and features, filtering for attention. Columns (2) and (4) show results with analytic weighting by population-weights.*

	Dependent variable:			
	Legitimacy (1-7) Sample	Legitimacy (1-7) Population	Choice Sample	Choice Population
	(1)	(2)	(3)	(4)
Coin	-1.659***	-1.637***	-0.461***	-0.466***
	(0.044)	(0.046)	(0.014)	(0.015)
	t = -37.307	t = -35.577	t = -32.751	t = -31.797
	p = 0.000	p = 0.000	p = 0.000	p = 0.000
Algo	-1.422***	-1.415***	-0.404***	-0.405***
	(0.044)	(0.046)	(0.020)	(0.023)
	t = -32.633	t = -30.802	t = -19.847	t = -17.753
	p = 0.000	p = 0.000	p = 0.000	p = 0.000
Zuck	-1.657***	-1.627***	-0.462***	-0.467***
	(0.047)	(0.049)	(0.010)	(0.009)
	t = -35.536	t = -32.865	t = -47.829	t = -50.080
	p = 0.000	p = 0.000	p = 0.000	p = 0.000
NatRep	-0.891***	-0.883***	-0.310***	-0.311***
	(0.051)	(0.053)	(0.023)	(0.028)
	t = -17.316	t = -16.763	t = -13.275	t = -11.284
	p = 0.000	p = 0.000	p = 0.000	p = 0.000
SMUsers	-1.209***	-1.198***	-0.374***	-0.378***
	(0.054)	(0.057)	(0.016)	(0.020)

	t = -22.427	t = -21.063	t = -23.966	t = -19.156
	p = 0.000	p = 0.000	p = 0.000	p = 0.000
PolBal	-0.765***	-0.750***	-0.285***	-0.275***
	(0.053)	(0.054)	(0.022)	(0.026)
	t = -14.412	t = -13.912	t = -13.054	t = -10.644
	p = 0.000	p = 0.000	p = 0.000	p = 0.000
FCers	-0.034	-0.023	-0.019*	-0.033**
	(0.028)	(0.029)	(0.009)	(0.011)
	t = -1.192	t = -0.815	t = -2.214	t = -2.928
	p = 0.234	p = 0.416	p = 0.027	p = 0.004
Journ	-0.570***	-0.559***	-0.183***	-0.188***
	(0.029)	(0.030)	(0.021)	(0.027)
	t = -19.547	t = -18.379	t = -8.832	t = -6.978
	p = 0.000	p = 0.000	p = 0.000	p = 0.000
Discuss	0.184***	0.187***	0.054***	0.048**
	(0.023)	(0.025)	(0.010)	(0.017)
	t = 8.025	t = 7.612	t = 5.275	t = 2.786
	p = 0.000	p = 0.000	p = 0.00000	p = 0.006
NatRep:Size30	0.402***	0.429***	0.168***	0.159***
	(0.064)	(0.065)	(0.032)	(0.034)
	t = 6.260	t = 6.607	t = 5.345	t = 4.662
	p = 0.000	p = 0.000	p = 0.00000	p = 0.00001
NatRep:Size3000	0.459***	0.460***	0.231***	0.219***
	(0.061)	(0.064)	(0.038)	(0.034)
	t = 7.508	t = 7.133	t = 6.100	t = 6.354
	p = 0.000	p = 0.000	p = 0.000	p = 0.000
SMUsers:Size30	0.421***	0.421***	0.157***	0.147***

	(0.065)	(0.068)	(0.020)	(0.022)
	t = 6.443	t = 6.212	t = 7.814	t = 6.553
	p = 0.000	p = 0.000	p = 0.000	p = 0.000
SMUsers:Size3000	0.575^{***}	0.547^{***}	0.196^{***}	0.186^{***}
	(0.064)	(0.067)	(0.032)	(0.034)
	t = 9.024	t = 8.178	t = 6.089	t = 5.398
	p = 0.000	p = 0.000	p = 0.000	p = 0.00000
PolBal:Size30	0.301^{***}	0.285^{***}	0.138^{***}	0.127^{***}
	(0.061)	(0.062)	(0.026)	(0.028)
	t = 4.930	t = 4.617	t = 5.209	t = 4.560
	p = 0.00000	p = 0.00001	p = 0.00000	p = 0.00001
PolBal:Size3000	0.325^{***}	0.347^{***}	0.176^{***}	0.152^{***}
	(0.066)	(0.067)	(0.018)	(0.025)
	t = 4.914	t = 5.164	t = 9.776	t = 6.069
	p = 0.00000	p = 0.00000	p = 0.000	p = 0.000
NatRep:Qual	0.359^{***}	0.343^{***}	0.152^{***}	0.141^{***}
	(0.062)	(0.066)	(0.017)	(0.023)
	t = 5.780	t = 5.189	t = 8.741	t = 6.217
	p = 0.000	p = 0.00000	p = 0.000	p = 0.000
SMUsers:Qual	0.371^{***}	0.378^{***}	0.153^{***}	0.148^{***}
	(0.065)	(0.068)	(0.024)	(0.029)
	t = 5.689	t = 5.538	t = 6.390	t = 5.058
	p = 0.000	p = 0.00000	p = 0.000	p = 0.00000
PolBal:Qual	0.306^{***}	0.290^{***}	0.167^{***}	0.151^{***}
	(0.066)	(0.066)	(0.031)	(0.040)
	t = 4.649	t = 4.386	t = 5.352	t = 3.825
	p = 0.00001	p = 0.00002	p = 0.00000	p = 0.0002

NatRep:Discuss	-0.002	-0.005	0.024	0.026
	(0.066)	(0.071)	(0.039)	(0.046)
	t = -0.027	t = -0.073	t = 0.615	t = 0.563
	p = 0.979	p = 0.942	p = 0.539	p = 0.574
SMUsers:Discuss	0.076	0.092	0.022	0.033
	(0.064)	(0.067)	(0.026)	(0.031)
	t = 1.194	t = 1.381	t = 0.834	t = 1.056
	p = 0.233	p = 0.168	p = 0.405	p = 0.291
PolBal:Discuss	-0.095	-0.070	-0.012	-0.013
	(0.069)	(0.071)	(0.030)	(0.036)
	t = -1.376	t = -0.984	t = -0.402	t = -0.362
	p = 0.169	p = 0.326	p = 0.688	p = 0.718
Discuss:FCers	-0.002	-0.007	0.008**	0.022*
	(0.029)	(0.031)	(0.002)	(0.011)
	t = -0.058	t = -0.212	t = 3.282	t = 2.042
	p = 0.954	p = 0.832	p = 0.002	p = 0.042
Discuss:Journ	-0.074*	-0.056	-0.010	0.003
	(0.030)	(0.032)	(0.020)	(0.028)
	t = -2.478	t = -1.741	t = -0.489	t = 0.110
	p = 0.014	p = 0.082	p = 0.625	p = 0.913
NatRep:Size30:Qual	-0.070	-0.073	0.015	0.024
	(0.092)	(0.096)	(0.030)	(0.038)
	t = -0.768	t = -0.761	t = 0.481	t = 0.633
	p = 0.443	p = 0.447	p = 0.631	p = 0.527
NatRep:Size3000:Qual	0.003	0.010	-0.006	0.009
	(0.092)	(0.097)	(0.043)	(0.047)
	t = 0.033	t = 0.101	t = -0.131	t = 0.186
	p = 0.974	p = 0.920	p = 0.897	p = 0.853
SMUsers:Size30:Qual	0.015	0.021	-0.001	-0.015
	(0.085)	(0.089)	(0.033)	(0.040)
	t = 0.183	t = 0.241	t = -0.029	t = -0.373

	p = 0.855	p = 0.810	p = 0.977	p = 0.709
SMUsers:Size3000:Qual	-0.064	-0.059	0.001	0.003
	(0.089)	(0.095)	(0.043)	(0.048)
	t = -0.717	t = -0.628	t = 0.030	t = 0.057
	p = 0.474	p = 0.531	p = 0.977	p = 0.955
PolBal:Size30:Qual	0.059	0.079	0.021	0.033
	(0.085)	(0.088)	(0.036)	(0.044)
	t = 0.691	t = 0.901	t = 0.594	t = 0.746
	p = 0.490	p = 0.368	p = 0.553	p = 0.456
PolBal:Size3000:Qual	0.059	0.057	0.014	0.039
	(0.093)	(0.094)	(0.038)	(0.050)
	t = 0.633	t = 0.612	t = 0.382	t = 0.766
	p = 0.528	p = 0.541	p = 0.703	p = 0.444
NatRep:Size30:Discuss	0.034	0.019	-0.033	-0.033
	(0.090)	(0.094)	(0.046)	(0.052)
	t = 0.379	t = 0.200	t = -0.703	t = -0.628
	p = 0.706	p = 0.842	p = 0.483	p = 0.530
NatRep:Size3000:Discuss	0.003	0.022	-0.063	-0.056
	(0.087)	(0.093)	(0.063)	(0.061)
	t = 0.038	t = 0.241	t = -1.011	t = -0.925
	p = 0.970	p = 0.810	p = 0.312	p = 0.355
SMUsers:Size30:Discuss	-0.077	-0.051	-0.014	0.006
	(0.093)	(0.096)	(0.038)	(0.040)
	t = -0.833	t = -0.536	t = -0.360	t = 0.147
	p = 0.405	p = 0.593	p = 0.719	p = 0.884
SMUsers:Size3000:Discuss	-0.129	-0.099	-0.067	-0.068
	(0.087)	(0.094)	(0.042)	(0.048)
	t = -1.486	t = -1.056	t = -1.608	t = -1.409
	p = 0.138	p = 0.292	p = 0.108	p = 0.159
PolBal:Size30:Discuss	0.094	0.059	0.044	0.047

	(0.092)	(0.095)	(0.049)	(0.065)
	t = 1.026	t = 0.620	t = 0.898	t = 0.724
	p = 0.306	p = 0.535	p = 0.370	p = 0.470
PolBal:Size3000:Discuss	0.093	0.046	0.053	0.072
	(0.093)	(0.096)	(0.047)	(0.059)
	t = 1.006	t = 0.480	t = 1.138	t = 1.226
	p = 0.315	p = 0.632	p = 0.255	p = 0.221
NatRep:Qual:Discuss	-0.033	-0.0001	0.006	0.024
	(0.087)	(0.097)	(0.037)	(0.042)
	t = -0.372	t = -0.001	t = 0.174	t = 0.576
	p = 0.710	p = 1.000	p = 0.862	p = 0.565
SMUsers:Qual:Discuss	-0.120	-0.129	-0.057*	-0.051*
	(0.097)	(0.104)	(0.023)	(0.024)
	t = -1.233	t = -1.239	t = -2.439	t = -2.093
	p = 0.218	p = 0.216	p = 0.015	p = 0.037
PolBal:Qual:Discuss	0.027	0.040	-0.013	-0.010
	(0.090)	(0.092)	(0.058)	(0.072)
	t = 0.301	t = 0.431	t = -0.231	t = -0.138
	p = 0.764	p = 0.667	p = 0.818	p = 0.891
NatRep:Size30:Qual:Discuss	0.080	0.083	0.003	-0.0004
	(0.127)	(0.137)	(0.043)	(0.054)
	t = 0.628	t = 0.604	t = 0.079	t = -0.008
	p = 0.531	p = 0.546	p = 0.937	p = 0.994
NatRep:Size3000:Qual:Discuss	-0.048	-0.094	-0.018	-0.054
	(0.130)	(0.141)	(0.078)	(0.080)
	t = -0.371	t = -0.671	t = -0.234	t = -0.675
	p = 0.711	p = 0.503	p = 0.815	p = 0.500
SMUsers:Size30:Qual:Discuss	0.125	0.091	0.057	0.065
	(0.132)	(0.139)	(0.042)	(0.039)
	t = 0.953	t = 0.658	t = 1.349	t = 1.662
	p = 0.341	p = 0.511	p = 0.178	p = 0.097

SMUsers:Size3000:Qual:Discuss	0.137	0.145	0.117*	0.119*
	(0.129)	(0.137)	(0.054)	(0.052)
	t = 1.063	t = 1.061	t = 2.170	t = 2.283
	p = 0.288	p = 0.289	p = 0.031	p = 0.023
PolBal:Size30:Qual:Discuss	-0.118	-0.107	-0.052	-0.052
	(0.119)	(0.125)	(0.064)	(0.076)
	t = -0.989	t = -0.855	t = -0.819	t = -0.689
	p = 0.323	p = 0.393	p = 0.413	p = 0.491
PolBal:Size3000:Qual:Discuss	-0.137	-0.109	-0.086	-0.095
	(0.132)	(0.137)	(0.059)	(0.075)
	t = -1.037	t = -0.793	t = -1.476	t = -1.261
	p = 0.300	p = 0.428	p = 0.141	p = 0.208
Constant	4.150***	4.138***	0.616***	0.622***
	(0.031)	(0.033)	(0.011)	(0.014)
	t = 132.927	t = 126.722	t = 57.144	t = 43.209
	p = 0.000	p = 0.000	p = 0.000	p = 0.000

Note: *p<.05, **p<.01, ***p<.001

OLS predicting legitimacy or choice of juries by multiple jury categories. Baseline levels are domain expert jury, size of 3, no qualifications or discussion.

7. Partisanship Moderation Analyses – Attention Filter.

Here we conduct robustness analyses on our partisanship moderation models, filtering for only participants who correctly answered a pre-treatment instructional attention check (*N*=1,741 participants answered pre-treatment attention check correctly, while 1,259 participants answered incorrectly and are excluded from the analyses in this section; see Q2 in survey codebook, adapted from (1)).

Table S7. *Legitimacy rating or jury choice predicted by category-level jury types, features, and partisanship, filtering for attention. Columns (2) and (4) show results with analytic weighting by population-weights.*

	Dependent variable:			
	Legitimacy (1-7) Sample	Legitimacy (1-7) Population	Choice Sample	Choice Population
	(1)	(2)	(3)	(4)
NonJury	-0.989***	-0.969***	-0.253***	-0.257***
	(0.049)	(0.049)	(0.024)	(0.024)
	t = -20.353	t = -19.744	t = -10.737	t = -10.595
	p = 0.000	p = 0.000	p = 0.000	p = 0.000
Layperson	-0.223***	-0.220***	-0.105***	-0.094***
	(0.058)	(0.058)	(0.022)	(0.023)
	t = -3.856	t = -3.796	t = -4.720	t = -4.084
	p = 0.0002	p = 0.0002	p = 0.00001	p = 0.00005
Discuss	0.073**	0.076**	0.029	0.024
	(0.026)	(0.029)	(0.017)	(0.018)
	t = 2.753	t = 2.654	t = 1.687	t = 1.326
	p = 0.006	p = 0.008	p = 0.092	p = 0.185
SubjectPartyInd	0.337***	0.344***	0.085***	0.077***

	(0.084)	(0.088)	(0.021)	(0.023)
	t = 4.007	t = 3.928	t = 3.967	t = 3.410
	p = 0.0001	p = 0.0001	p = 0.0001	p = 0.001
SubjectPartyDem	1.038***	1.072***	0.167***	0.161***
	(0.062)	(0.064)	(0.015)	(0.017)
	t = 16.650	t = 16.653	t = 10.933	t = 9.602
	p = 0.000	p = 0.000	p = 0.000	p = 0.000
Layperson:Size30	0.302***	0.293***	0.152***	0.131***
	(0.063)	(0.065)	(0.026)	(0.029)
	t = 4.814	t = 4.529	t = 5.747	t = 4.518
	p = 0.00001	p = 0.00001	p = 0.000	p = 0.00001
Layperson:Size3000	0.472***	0.481***	0.203***	0.175***
	(0.068)	(0.068)	(0.029)	(0.030)
	t = 6.902	t = 7.029	t = 6.930	t = 5.939
	p = 0.000	p = 0.000	p = 0.000	p = 0.000
Layperson:Qual	0.240***	0.221**	0.163***	0.141***
	(0.065)	(0.072)	(0.023)	(0.030)
	t = 3.694	t = 3.050	t = 7.080	t = 4.709
	p = 0.0003	p = 0.003	p = 0.000	p = 0.00001
Layperson:Discuss	0.015	0.033	0.050*	0.044
	(0.062)	(0.064)	(0.024)	(0.024)
	t = 0.246	t = 0.524	t = 2.104	t = 1.810
	p = 0.806	p = 0.601	p = 0.036	p = 0.071
NonJury:SubjectPartyInd	-0.208*	-0.211*	-0.116**	-0.108**
	(0.089)	(0.091)	(0.036)	(0.037)

	t = -2.348	t = -2.319	t = -3.253	t = -2.959
	p = 0.019	p = 0.021	p = 0.002	p = 0.004
NonJury:SubjectPartyDem	-0.801***	-0.817***	-0.222***	-0.209***
	(0.069)	(0.072)	(0.021)	(0.024)
	t = -11.582	t = -11.388	t = -10.717	t = -8.846
	p = 0.000	p = 0.000	p = 0.000	p = 0.000
Layperson:SubjectPartyInd	-0.418***	-0.438***	-0.141***	-0.140***
	(0.100)	(0.102)	(0.036)	(0.040)
	t = -4.177	t = -4.302	t = -3.874	t = -3.546
	p = 0.00003	p = 0.00002	p = 0.0002	p = 0.0004
Layperson:SubjectPartyDem	-1.024***	-1.006***	-0.267***	-0.272***
	(0.079)	(0.081)	(0.029)	(0.031)
	t = -12.951	t = -12.405	t = -9.056	t = -8.879
	p = 0.000	p = 0.000	p = 0.000	p = 0.000
Discuss:SubjectPartyInd	0.076	0.095*	0.013	0.036
	(0.043)	(0.047)	(0.023)	(0.026)
	t = 1.759	t = 2.000	t = 0.550	t = 1.344
	p = 0.079	p = 0.046	p = 0.583	p = 0.179
Discuss:SubjectPartyDem	0.158***	0.152***	0.046*	0.055**
	(0.037)	(0.039)	(0.019)	(0.021)
	t = 4.285	t = 3.860	t = 2.388	t = 2.665
	p = 0.00002	p = 0.0002	p = 0.017	p = 0.008
Layperson:Size30:Qual	0.086	0.128	-0.022	0.001

	(0.084)	(0.093)	(0.040)	(0.045)
	t = 1.023	t = 1.370	t = -0.534	t = 0.022
	p = 0.307	p = 0.171	p = 0.594	p = 0.983
Layperson:Size3000:Qual	-0.001	0.014	-0.037	-0.005
	(0.091)	(0.098)	(0.027)	(0.031)
	t = -0.010	t = 0.141	t = -1.379	t = -0.169
	p = 0.992	p = 0.889	p = 0.169	p = 0.866
Layperson:Size30:Discuss	0.089	0.146	-0.032	-0.008
	(0.083)	(0.088)	(0.022)	(0.030)
	t = 1.068	t = 1.661	t = -1.492	t = -0.255
	p = 0.286	p = 0.097	p = 0.136	p = 0.799
Layperson:Size3000:Discuss	-0.066	-0.054	-0.072**	-0.042
	(0.089)	(0.090)	(0.028)	(0.039)
	t = -0.745	t = -0.594	t = -2.609	t = -1.084
	p = 0.457	p = 0.553	p = 0.010	p = 0.279
Layperson:Qual:Discuss	0.030	0.035	-0.083*	-0.060
	(0.086)	(0.096)	(0.037)	(0.042)
	t = 0.344	t = 0.362	t = -2.251	t = -1.444
	p = 0.731	p = 0.718	p = 0.025	p = 0.149
Layperson:Size30:SubjectPartyInd	0.087	0.129	0.064**	0.075*
	(0.100)	(0.101)	(0.021)	(0.038)
	t = 0.863	t = 1.278	t = 3.084	t = 1.985
	p = 0.388	p = 0.202	p = 0.003	p = 0.048

Layperson:Size3000:SubjectPartyInd	-0.070	-0.084	0.021	0.028
	(0.111)	(0.111)	(0.046)	(0.043)
	t = -0.628	t = -0.756	t = 0.453	t = 0.663
	p = 0.530	p = 0.450	p = 0.651	p = 0.508
Layperson:Size30:SubjectPartyDem	0.153	0.159	-0.021	0.001
	(0.084)	(0.086)	(0.039)	(0.038)
	t = 1.820	t = 1.834	t = -0.545	t = 0.032
	p = 0.069	p = 0.067	p = 0.587	p = 0.975
Layperson:Size3000:SubjectPartyDem	0.005	-0.016	-0.019	0.003
	(0.088)	(0.090)	(0.037)	(0.033)
	t = 0.060	t = -0.179	t = -0.519	t = 0.105
	p = 0.953	p = 0.858	p = 0.604	p = 0.917
Layperson:Qual:SubjectPartyInd	0.177	0.225^{*}	-0.030	-0.014
	(0.107)	(0.109)	(0.040)	(0.048)
	t = 1.643	t = 2.056	t = -0.742	t = -0.298
	p = 0.101	p = 0.040	p = 0.458	p = 0.766
Layperson:Qual:SubjectPartyDem	0.172^{*}	0.175	-0.005	0.013
	(0.083)	(0.091)	(0.035)	(0.048)
	t = 2.056	t = 1.924	t = -0.146	t = 0.263
	p = 0.040	p = 0.055	p = 0.884	p = 0.793
Layperson:Discuss:SubjectPartyInd	0.101	0.099	-0.044	-0.055
	(0.104)	(0.105)	(0.052)	(0.039)
	t = 0.970	t = 0.945	t = -0.849	t = -1.411
	p = 0.332	p = 0.345	p = 0.397	p = 0.159

Layperson:Discuss:SubjectPartyDem	-0.027	-0.047	-0.077*	-0.073*
	(0.083)	(0.087)	(0.036)	(0.036)
	t = -0.323	t = -0.540	t = -2.124	t = -2.034
	p = 0.747	p = 0.589	p = 0.034	p = 0.042
Layperson:Size30:Qual:Discuss	-0.072	-0.140	0.077	0.050
	(0.114)	(0.130)	(0.047)	(0.062)
	t = -0.628	t = -1.077	t = 1.648	t = 0.796
	p = 0.531	p = 0.282	p = 0.100	p = 0.427
Layperson:Size3000:Qual:Discuss	-0.007	-0.032	0.107*	0.070
	(0.130)	(0.134)	(0.044)	(0.053)
	t = -0.057	t = -0.240	t = 2.413	t = 1.326
	p = 0.955	p = 0.811	p = 0.016	p = 0.185
Layperson:Size30:Qual:SubjectPartyInd	-0.184	-0.273	-0.007	-0.028
	(0.138)	(0.146)	(0.056)	(0.082)
	t = -1.339	t = -1.876	t = -0.122	t = -0.338
	p = 0.181	p = 0.061	p = 0.903	p = 0.736
Layperson:Size3000:Qual:SubjectPartyInd	-0.164	-0.186	0.027	0.025
	(0.153)	(0.157)	(0.055)	(0.070)
	t = -1.076	t = -1.190	t = 0.485	t = 0.351
	p = 0.282	p = 0.235	p = 0.628	p = 0.726
Layperson:Size30:Qual:SubjectPartyDem	-0.107	-0.137	0.081	0.048
	(0.113)	(0.122)	(0.057)	(0.060)
	t = -0.945	t = -1.123	t = 1.418	t = 0.790
	p = 0.345	p = 0.262	p = 0.157	p = 0.430
Layperson:Size3000:Qual:SubjectPartyDem	0.085	0.068	0.089*	0.048

	(0.119)	(0.127)	(0.034)	(0.048)
	t = 0.719	t = 0.530	t = 2.571	t = 1.010
	p = 0.473	p = 0.596	p = 0.011	p = 0.313
Layperson:Size30:Discuss:SubjectPartyInd	-0.175	-0.300*	-0.042	-0.072
	(0.140)	(0.146)	(0.053)	(0.054)
	t = -1.247	t = -2.059	t = -0.786	t = -1.350
	p = 0.213	p = 0.040	p = 0.432	p = 0.178
Layperson:Size3000:Discuss:SubjectPartyInd	-0.029	-0.051	0.068	0.046
	(0.138)	(0.140)	(0.078)	(0.080)
	t = -0.214	t = -0.365	t = 0.875	t = 0.573
	p = 0.831	p = 0.716	p = 0.382	p = 0.567
Layperson:Size30:Discuss:SubjectPartyDem	-0.104	-0.194	0.088	0.065
	(0.113)	(0.117)	(0.046)	(0.057)
	t = -0.923	t = -1.657	t = 1.925	t = 1.158
	p = 0.356	p = 0.098	p = 0.055	p = 0.247
Layperson:Size3000:Discuss:SubjectPartyDem	0.154	0.135	0.086	0.051
	(0.115)	(0.120)	(0.051)	(0.058)
	t = 1.335	t = 1.122	t = 1.676	t = 0.874
	p = 0.182	p = 0.263	p = 0.094	p = 0.382
Layperson:Qual:Discuss:SubjectPartyInd	-0.334*	-0.360*	0.122	0.093
	(0.145)	(0.155)	(0.071)	(0.065)
	t = -2.297	t = -2.331	t = 1.716	t = 1.427
	p = 0.022	p = 0.020	p = 0.087	p = 0.154
Layperson:Qual:Discuss:SubjectPartyDem	-0.034	-0.011	0.099*	0.085
	(0.115)	(0.124)	(0.039)	(0.055)
	t = -0.297	t = -0.089	t = 2.526	t = 1.555

	p = 0.767	p = 0.929	p = 0.012	p = 0.120
Layperson:Size30:Qual:Discuss:SubjectPartyInd	0.270	0.401	-0.043	0.017
	(0.190)	(0.215)	(0.083)	(0.104)
	t = 1.420	t = 1.860	t = -0.511	t = 0.158
	p = 0.156	p = 0.063	p = 0.610	p = 0.875
Layperson:Size3000:Qual:Discuss:SubjectPartyInd	0.325	0.372	-0.176*	-0.162
	(0.211)	(0.216)	(0.087)	(0.100)
	t = 1.545	t = 1.722	t = -2.014	t = -1.621
	p = 0.123	p = 0.085	p = 0.044	p = 0.106
Layperson:Size30:Qual:Discuss:SubjectPartyDem	0.123	0.208	-0.157*	-0.123
	(0.157)	(0.170)	(0.079)	(0.098)
	t = 0.780	t = 1.226	t = -1.975	t = -1.260
	p = 0.436	p = 0.221	p = 0.049	p = 0.208
Layperson:Size3000:Qual:Discuss:SubjectPartyDem	-0.149	-0.106	-0.168*	-0.131
	(0.170)	(0.180)	(0.066)	(0.090)
	t = -0.873	t = -0.591	t = -2.554	t = -1.463
	p = 0.383	p = 0.555	p = 0.011	p = 0.144
Constant	3.421***	3.398***	0.457***	0.460***
	(0.051)	(0.053)	(0.014)	(0.014)
	t = 66.627	t = 63.891	t = 32.621	t = 31.904
	p = 0.000	p = 0.000	p = 0.000	p = 0.000

Note: *p<.05, **p<.01, ***p<.001

OLS predicting legitimacy or choice of juries by non-jury, layperson, and expert categories, with partisanship moderator. Baseline levels are expert jury, size of 3, no qualifications or discussion; and Republican party.

Table S8. *Legitimacy rating or jury choice predicted by jury-specific levels, features, and partisanship, filtering for attention. Columns (2) and (4) show results with analytic weighting by population-weights.*

	Dependent variable:			
	Legitimacy (1-7) Sample	Legitimacy (1-7) Population	Choice Sample	Choice Population
	(1)	(2)	(3)	(4)
Coin	-1.332***	-1.310***	-0.371***	-0.381***
	(0.073)	(0.074)	(0.019)	(0.023)
	t = -18.235	t = -17.810	t = -19.785	t = -16.710
	p = 0.000	p = 0.000	p = 0.000	p = 0.000
Algo	-1.159***	-1.145***	-0.335***	-0.337***
	(0.070)	(0.072)	(0.030)	(0.031)
	t = -16.531	t = -15.918	t = -11.288	t = -10.744
	p = 0.000	p = 0.000	p = 0.000	p = 0.000
Zuck	-1.540***	-1.509***	-0.423***	-0.438***
	(0.074)	(0.078)	(0.024)	(0.023)
	t = -20.775	t = -19.433	t = -17.348	t = -19.470
	p = 0.000	p = 0.000	p = 0.000	p = 0.000
NatRep	-0.501***	-0.522***	-0.216***	-0.208***
	(0.090)	(0.095)	(0.035)	(0.042)
	t = -5.593	t = -5.514	t = -6.115	t = -4.933
	p = 0.00000	p = 0.00000	p = 0.000	p = 0.00000

SMUsers	-0.718***	-0.718***	-0.280***	-0.278***
	(0.087)	(0.092)	(0.037)	(0.036)
	t = -8.222	t = -7.794	t = -7.627	t = -7.606
	p = 0.000	p = 0.000	p = 0.000	p = 0.000
PolBal	-0.489***	-0.451***	-0.179***	-0.171***
	(0.092)	(0.094)	(0.035)	(0.034)
	t = -5.310	t = -4.803	t = -5.078	t = -5.031
	p = 0.00000	p = 0.00001	p = 0.00000	p = 0.00000
FCers	-0.296***	-0.284***	-0.121***	-0.132***
	(0.053)	(0.052)	(0.028)	(0.031)
	t = -5.626	t = -5.459	t = -4.385	t = -4.341
	p = 0.00000	p = 0.00000	p = 0.00002	p = 0.00002
Journ	-0.765***	-0.771***	-0.248***	-0.252***
	(0.054)	(0.055)	(0.030)	(0.034)
	t = -14.208	t = -13.916	t = -8.228	t = -7.497
	p = 0.000	p = 0.000	p = 0.000	p = 0.000
Discuss	0.080*	0.065	0.010	-0.001
	(0.040)	(0.043)	(0.022)	(0.029)
	t = 1.989	t = 1.518	t = 0.448	t = -0.051
	p = 0.047	p = 0.129	p = 0.654	p = 0.960
SubjectPartyInd	0.174	0.166	0.026	0.018
	(0.090)	(0.093)	(0.028)	(0.035)
	t = 1.934	t = 1.788	t = 0.915	t = 0.520

	p = 0.054	p = 0.074	p = 0.361	p = 0.603
SubjectPartyDem	0.775***	0.809***	0.072**	0.070**
	(0.070)	(0.072)	(0.023)	(0.024)
	t = 11.135	t = 11.205	t = 3.124	t = 2.919
	p = 0.000	p = 0.000	p = 0.002	p = 0.004
NatRep:Size30	0.310**	0.334**	0.135***	0.095*
	(0.114)	(0.115)	(0.040)	(0.043)
	t = 2.707	t = 2.900	t = 3.361	t = 2.180
	p = 0.007	p = 0.004	p = 0.001	p = 0.030
NatRep:Size3000	0.383***	0.438***	0.173***	0.143***
	(0.112)	(0.120)	(0.040)	(0.040)
	t = 3.431	t = 3.658	t = 4.297	t = 3.545
	p = 0.001	p = 0.0003	p = 0.00002	p = 0.0004
SMUsers:Size30	0.194	0.194	0.163***	0.153***
	(0.105)	(0.108)	(0.036)	(0.038)
	t = 1.850	t = 1.796	t = 4.485	t = 4.015
	p = 0.065	p = 0.073	p = 0.00001	p = 0.0001
SMUsers:Size3000	0.443***	0.413***	0.213***	0.196***
	(0.114)	(0.120)	(0.040)	(0.035)
	t = 3.874	t = 3.428	t = 5.370	t = 5.563
	p = 0.0002	p = 0.001	p = 0.00000	p = 0.00000
PolBal:Size30	0.455***	0.400***	0.164**	0.152**
	(0.114)	(0.121)	(0.063)	(0.053)
	t = 4.004	t = 3.315	t = 2.606	t = 2.879
	p = 0.0001	p = 0.001	p = 0.010	p = 0.004

PolBal:Size3000	0.565***	0.567***	0.213***	0.180***
	(0.124)	(0.123)	(0.043)	(0.039)
	t = 4.564	t = 4.599	t = 4.917	t = 4.577
	p = 0.00001	p = 0.00001	p = 0.00000	p = 0.00001
NatRep:Qual	0.280*	0.267*	0.158***	0.131*
	(0.113)	(0.119)	(0.043)	(0.055)
	t = 2.478	t = 2.237	t = 3.649	t = 2.368
	p = 0.014	p = 0.026	p = 0.0003	p = 0.018
SMUsers:Qual	0.207	0.214	0.134*	0.134*
	(0.121)	(0.131)	(0.060)	(0.065)
	t = 1.717	t = 1.639	t = 2.219	t = 2.062
	p = 0.087	p = 0.102	p = 0.027	p = 0.040
PolBal:Qual	0.176	0.125	0.173***	0.138**
	(0.124)	(0.127)	(0.039)	(0.050)
	t = 1.415	t = 0.977	t = 4.459	t = 2.755
	p = 0.157	p = 0.329	p = 0.00001	p = 0.006
NatRep:Discuss	-0.074	-0.009	0.077	0.078*
	(0.114)	(0.126)	(0.043)	(0.037)
	t = -0.653	t = -0.071	t = 1.774	t = 2.142
	p = 0.514	p = 0.944	p = 0.077	p = 0.033
SMUsers:Discuss	-0.029	-0.037	0.020	0.018
	(0.117)	(0.121)	(0.044)	(0.047)
	t = -0.250	t = -0.306	t = 0.451	t = 0.397
	p = 0.803	p = 0.760	p = 0.653	p = 0.692
PolBal:Discuss	0.069	0.109	0.080*	0.078*
	(0.122)	(0.123)	(0.040)	(0.034)

	t = 0.571	t = 0.884	t = 1.985	t = 2.297
	p = 0.568	p = 0.377	p = 0.048	p = 0.022
Discuss:FCers	0.038	0.057	0.056	0.069
	(0.052)	(0.056)	(0.035)	(0.041)
	t = 0.736	t = 1.027	t = 1.615	t = 1.692
	p = 0.462	p = 0.305	p = 0.107	p = 0.091
Discuss:Journ	-0.061	-0.025	0.003	0.007
	(0.053)	(0.059)	(0.037)	(0.041)
	t = -1.156	t = -0.424	t = 0.088	t = 0.158
	p = 0.248	p = 0.672	p = 0.930	p = 0.875
Coin:SubjectPartyInd	-0.085	-0.055	-0.041	-0.034
	(0.126)	(0.133)	(0.035)	(0.049)
	t = -0.675	t = -0.411	t = -1.160	t = -0.696
	p = 0.500	p = 0.682	p = 0.247	p = 0.487
Coin:SubjectPartyDem	-0.711***	-0.721***	-0.188***	-0.181***
	(0.099)	(0.100)	(0.023)	(0.028)
	t = -7.217	t = -7.180	t = -8.021	t = -6.472
	p = 0.000	p = 0.000	p = 0.000	p = 0.000
Algo:SubjectPartyInd	-0.139	-0.137	-0.068	-0.064
	(0.121)	(0.122)	(0.062)	(0.059)
	t = -1.146	t = -1.126	t = -1.094	t = -1.078
	p = 0.252	p = 0.261	p = 0.274	p = 0.282
Algo:SubjectPartyDem	-0.549***	-0.579***	-0.127**	-0.128**
	(0.097)	(0.102)	(0.041)	(0.043)

	t = -5.652	t = -5.680	t = -3.084	t = -2.973
	p = 0.00000	p = 0.000	p = 0.003	p = 0.003
Zuck:SubjectPartyInd	0.093	0.101	-0.062	-0.051
	(0.123)	(0.130)	(0.039)	(0.055)
	t = 0.757	t = 0.774	t = -1.611	t = -0.923
	p = 0.450	p = 0.440	p = 0.108	p = 0.356
Zuck:SubjectPartyDem	-0.350***	-0.363***	-0.064*	-0.047
	(0.105)	(0.110)	(0.029)	(0.025)
	t = -3.343	t = -3.305	t = -2.223	t = -1.893
	p = 0.001	p = 0.001	p = 0.027	p = 0.059
NatRep:SubjectPartyInd	-0.218	-0.197	-0.065	-0.068
	(0.143)	(0.146)	(0.036)	(0.042)
	t = -1.522	t = -1.348	t = -1.828	t = -1.633
	p = 0.129	p = 0.178	p = 0.068	p = 0.103
NatRep:SubjectPartyDem	-0.784***	-0.735***	-0.173***	-0.187***
	(0.117)	(0.122)	(0.040)	(0.052)
	t = -6.674	t = -6.000	t = -4.347	t = -3.617
	p = 0.000	p = 0.000	p = 0.00002	p = 0.0003
SMUsers:SubjectPartyInd	-0.457**	-0.486**	-0.076	-0.085
	(0.153)	(0.158)	(0.079)	(0.078)
	t = -2.996	t = -3.080	t = -0.956	t = -1.084
	p = 0.003	p = 0.003	p = 0.340	p = 0.279

SMUsers:SubjectPartyDem	-0.912***	-0.874***	-0.169**	-0.180**
	(0.118)	(0.126)	(0.052)	(0.057)
	t = -7.699	t = -6.959	t = -3.261	t = -3.137
	p = 0.000	p = 0.000	p = 0.002	p = 0.002
PolBal:SubjectPartyInd	-0.156	-0.161	-0.122*	-0.109
	(0.144)	(0.144)	(0.059)	(0.067)
	t = -1.081	t = -1.114	t = -2.059	t = -1.628
	p = 0.280	p = 0.266	p = 0.040	p = 0.104
PolBal:SubjectPartyDem	-0.585***	-0.627***	-0.177***	-0.181***
	(0.122)	(0.124)	(0.041)	(0.048)
	t = -4.802	t = -5.046	t = -4.327	t = -3.768
	p = 0.00001	p = 0.00000	p = 0.00002	p = 0.0002
FCers:SubjectPartyInd	0.246**	0.256***	0.118***	0.126***
	(0.079)	(0.077)	(0.034)	(0.032)
	t = 3.129	t = 3.341	t = 3.418	t = 3.906
	p = 0.002	p = 0.001	p = 0.001	p = 0.0001
FCers:SubjectPartyDem	0.465***	0.449***	0.176***	0.164**
	(0.065)	(0.066)	(0.045)	(0.054)
	t = 7.135	t = 6.764	t = 3.942	t = 3.030
	p = 0.000	p = 0.000	p = 0.0001	p = 0.003
Journ:SubjectPartyInd	0.243**	0.279***	0.060	0.049
	(0.083)	(0.084)	(0.041)	(0.045)
	t = 2.937	t = 3.322	t = 1.457	t = 1.089
	p = 0.004	p = 0.001	p = 0.146	p = 0.277

Journ:SubjectPartyDem	0.325***	0.338***	0.112**	0.109***
	(0.067)	(0.071)	(0.036)	(0.027)
	t = 4.819	t = 4.793	t = 3.147	t = 4.004
	p = 0.00001	p = 0.00001	p = 0.002	p = 0.0001
Discuss:SubjectPartyInd	0.101	0.143*	0.040	0.055
	(0.064)	(0.066)	(0.048)	(0.059)
	t = 1.577	t = 2.188	t = 0.824	t = 0.933
	p = 0.115	p = 0.029	p = 0.411	p = 0.351
Discuss:SubjectPartyDem	0.178***	0.188***	0.075**	0.076*
	(0.053)	(0.056)	(0.026)	(0.037)
	t = 3.385	t = 3.357	t = 2.850	t = 2.041
	p = 0.001	p = 0.001	p = 0.005	p = 0.042
NatRep:Size30:Qual	-0.092	-0.062	-0.001	0.045
	(0.158)	(0.162)	(0.052)	(0.072)
	t = -0.581	t = -0.381	t = -0.018	t = 0.629
	p = 0.562	p = 0.704	p = 0.986	p = 0.530
NatRep:Size3000:Qual	0.022	0.008	0.001	0.038
	(0.167)	(0.177)	(0.053)	(0.063)
	t = 0.130	t = 0.044	t = 0.028	t = 0.601
	p = 0.897	p = 0.965	p = 0.978	p = 0.548
SMUsers:Size30:Qual	0.167	0.176	-0.024	-0.040
	(0.150)	(0.159)	(0.071)	(0.077)
	t = 1.115	t = 1.104	t = -0.332	t = -0.519
	p = 0.266	p = 0.270	p = 0.741	p = 0.604
SMUsers:Size3000:Qual	0.025	0.037	-0.038	-0.051

	(0.163)	(0.181)	(0.068)	(0.070)
	t = 0.155	t = 0.204	t = -0.558	t = -0.731
	p = 0.878	p = 0.839	p = 0.578	p = 0.465
PolBal:Size30:Qual	0.111	0.203	-0.053	-0.022
	(0.155)	(0.162)	(0.089)	(0.084)
	t = 0.714	t = 1.253	t = -0.594	t = -0.265
	p = 0.476	p = 0.211	p = 0.553	p = 0.792
PolBal:Size3000:Qual	0.006	0.053	-0.056	0.013
	(0.165)	(0.164)	(0.053)	(0.060)
	t = 0.034	t = 0.320	t = -1.069	t = 0.223
	p = 0.973	p = 0.750	p = 0.286	p = 0.824
NatRep:Size30:Discuss	0.186	0.212	-0.077	-0.044
	(0.158)	(0.163)	(0.063)	(0.055)
	t = 1.176	t = 1.307	t = -1.230	t = -0.809
	p = 0.240	p = 0.192	p = 0.219	p = 0.419
NatRep:Size3000:Discuss	0.104	0.063	-0.005	0.024
	(0.160)	(0.173)	(0.080)	(0.081)
	t = 0.647	t = 0.363	t = -0.059	t = 0.289
	p = 0.518	p = 0.717	p = 0.954	p = 0.773
SMUsers:Size30:Discuss	0.095	0.243	0.018	0.044
	(0.161)	(0.166)	(0.044)	(0.053)
	t = 0.594	t = 1.461	t = 0.402	t = 0.833
	p = 0.553	p = 0.145	p = 0.688	p = 0.405
SMUsers:Size3000:Discuss	-0.063	0.023	-0.071	-0.055

	(0.160)	(0.176)	(0.052)	(0.065)
	t = -0.395	t = 0.130	t = -1.353	t = -0.838
	p = 0.693	p = 0.897	p = 0.176	p = 0.403
PolBal:Size30:Discuss	-0.028	-0.010	-0.017	-0.004
	(0.157)	(0.161)	(0.057)	(0.050)
	t = -0.179	t = -0.064	t = -0.300	t = -0.072
	p = 0.859	p = 0.950	p = 0.764	p = 0.943
PolBal:Size3000:Discuss	-0.192	-0.189	-0.111*	-0.065
	(0.163)	(0.163)	(0.056)	(0.052)
	t = -1.183	t = -1.159	t = -1.981	t = -1.240
	p = 0.237	p = 0.247	p = 0.048	p = 0.215
NatRep:Qual:Discuss	0.149	0.134	-0.041	-0.017
	(0.154)	(0.166)	(0.056)	(0.051)
	t = 0.966	t = 0.807	t = -0.731	t = -0.327
	p = 0.335	p = 0.420	p = 0.465	p = 0.744
SMUsers:Qual:Discuss	0.018	0.046	-0.038	-0.033
	(0.175)	(0.193)	(0.047)	(0.055)
	t = 0.103	t = 0.237	t = -0.811	t = -0.604
	p = 0.918	p = 0.813	p = 0.418	p = 0.546
PolBal:Qual:Discuss	0.109	0.123	-0.092	-0.051
	(0.162)	(0.169)	(0.075)	(0.084)
	t = 0.670	t = 0.728	t = -1.228	t = -0.614
	p = 0.503	p = 0.467	p = 0.220	p = 0.540

NatRep:Size30:SubjectPartyInd	0.168	0.178	0.188*	0.257***
	(0.180)	(0.178)	(0.078)	(0.078)
	t = 0.938	t = 1.001	t = 2.417	t = 3.304
	p = 0.349	p = 0.317	p = 0.016	p = 0.001
NatRep:Size3000:SubjectPartyInd	0.005	-0.078	0.075*	0.106*
	(0.176)	(0.180)	(0.030)	(0.049)
	t = 0.031	t = -0.432	t = 2.518	t = 2.162
	p = 0.976	p = 0.667	p = 0.012	p = 0.031
NatRep:Size30:SubjectPartyDem	0.128	0.140	0.003	0.046
	(0.150)	(0.152)	(0.057)	(0.060)
	t = 0.852	t = 0.924	t = 0.060	t = 0.770
	p = 0.395	p = 0.356	p = 0.952	p = 0.442
NatRep:Size3000:SubjectPartyDem	0.161	0.087	0.078	0.102
	(0.142)	(0.151)	(0.055)	(0.065)
	t = 1.134	t = 0.577	t = 1.416	t = 1.578
	p = 0.257	p = 0.565	p = 0.157	p = 0.115
SMUsers:Size30:SubjectPartyInd	0.350	0.488**	0.062	0.057
	(0.189)	(0.187)	(0.094)	(0.087)
	t = 1.856	t = 2.615	t = 0.661	t = 0.658
	p = 0.064	p = 0.009	p = 0.509	p = 0.511
SMUsers:Size3000:SubjectPartyInd	0.305	0.321	0.075	0.058
	(0.182)	(0.185)	(0.084)	(0.075)
	t = 1.681	t = 1.736	t = 0.886	t = 0.774
	p = 0.093	p = 0.083	p = 0.376	p = 0.440
SMUsers:Size30:SubjectPartyDem	0.381**	0.320*	-0.060	-0.060
	(0.146)	(0.153)	(0.049)	(0.061)

	t = 2.605	t = 2.093	t = -1.220	t = -0.973
	p = 0.010	p = 0.037	p = 0.223	p = 0.331
SMUsers:Size3000:SubjectPartyDem	0.155	0.147	-0.075	-0.056
	(0.147)	(0.155)	(0.051)	(0.044)
	t = 1.055	t = 0.952	t = -1.470	t = -1.269
	p = 0.292	p = 0.342	p = 0.142	p = 0.205
PolBal:Size30:SubjectPartyInd	-0.254	-0.253	-0.037	-0.056
	(0.173)	(0.176)	(0.097)	(0.092)
	t = -1.471	t = -1.442	t = -0.385	t = -0.603
	p = 0.142	p = 0.150	p = 0.701	p = 0.547
PolBal:Size3000:SubjectPartyInd	-0.448*	-0.430*	-0.067	-0.065
	(0.188)	(0.188)	(0.086)	(0.085)
	t = -2.377	t = -2.283	t = -0.778	t = -0.755
	p = 0.018	p = 0.023	p = 0.437	p = 0.450
PolBal:Size30:SubjectPartyDem	-0.159	-0.096	-0.032	-0.016
	(0.144)	(0.149)	(0.063)	(0.054)
	t = -1.110	t = -0.646	t = -0.507	t = -0.289
	p = 0.268	p = 0.519	p = 0.612	p = 0.773
PolBal:Size3000:SubjectPartyDem	-0.297	-0.281	-0.057	-0.035
	(0.155)	(0.157)	(0.057)	(0.045)
	t = -1.917	t = -1.786	t = -0.994	t = -0.784
	p = 0.056	p = 0.075	p = 0.321	p = 0.434
NatRep:Qual:SubjectPartyInd	0.106	0.124	-0.075	-0.046

	(0.182)	(0.180)	(0.092)	(0.107)
	t = 0.584	t = 0.689	t = -0.813	t = -0.425
	p = 0.560	p = 0.491	p = 0.417	p = 0.671
NatRep:Qual:SubjectPartyDem	0.128	0.126	0.004	0.020
	(0.144)	(0.155)	(0.071)	(0.092)
	t = 0.888	t = 0.817	t = 0.054	t = 0.220
	p = 0.375	p = 0.415	p = 0.958	p = 0.826
SMUsers:Qual:SubjectPartyInd	0.365	0.439*	0.014	-0.012
	(0.200)	(0.203)	(0.102)	(0.100)
	t = 1.825	t = 2.160	t = 0.139	t = -0.121
	p = 0.069	p = 0.031	p = 0.890	p = 0.904
SMUsers:Qual:SubjectPartyDem	0.209	0.177	0.035	0.040
	(0.151)	(0.162)	(0.074)	(0.083)
	t = 1.381	t = 1.093	t = 0.468	t = 0.488
	p = 0.168	p = 0.275	p = 0.640	p = 0.626
PolBal:Qual:SubjectPartyInd	0.172	0.211	0.002	0.039
	(0.183)	(0.184)	(0.075)	(0.092)
	t = 0.939	t = 1.144	t = 0.025	t = 0.425
	p = 0.348	p = 0.253	p = 0.980	p = 0.671
PolBal:Qual:SubjectPartyDem	0.240	0.293	-0.031	-0.003
	(0.157)	(0.159)	(0.035)	(0.053)
	t = 1.526	t = 1.843	t = -0.895	t = -0.052
	p = 0.127	p = 0.066	p = 0.371	p = 0.959
NatRep:Discuss:SubjectPartyInd	-0.025	-0.123	-0.111	-0.134***
	(0.191)	(0.199)	(0.058)	(0.032)

	t = -0.133	t = -0.621	t = -1.922	t = -4.178
	p = 0.895	p = 0.535	p = 0.055	p = 0.00003
NatRep:Discuss:SubjectPartyDem	0.179	0.084	-0.090	-0.082
	(0.151)	(0.164)	(0.055)	(0.062)
	t = 1.187	t = 0.515	t = -1.619	t = -1.320
	p = 0.236	p = 0.607	p = 0.106	p = 0.187
SMUsers:Discuss:SubjectPartyInd	0.373*	0.486*	-0.020	-0.007
	(0.184)	(0.190)	(0.143)	(0.147)
	t = 2.032	t = 2.561	t = -0.139	t = -0.046
	p = 0.043	p = 0.011	p = 0.890	p = 0.964
SMUsers:Discuss:SubjectPartyDem	0.057	0.063	-0.012	0.012
	(0.150)	(0.155)	(0.050)	(0.057)
	t = 0.383	t = 0.403	t = -0.247	t = 0.218
	p = 0.703	p = 0.687	p = 0.806	p = 0.828
PolBal:Discuss:SubjectPartyInd	-0.041	-0.125	-0.047	-0.050
	(0.190)	(0.191)	(0.087)	(0.085)
	t = -0.215	t = -0.654	t = -0.533	t = -0.584
	p = 0.830	p = 0.514	p = 0.595	p = 0.560
PolBal:Discuss:SubjectPartyDem	-0.306	-0.317	-0.183**	-0.183*
	(0.161)	(0.166)	(0.056)	(0.073)
	t = -1.908	t = -1.916	t = -3.283	t = -2.507
	p = 0.057	p = 0.056	p = 0.002	p = 0.013

Discuss:FCers:SubjectPartyInd	-0.051	-0.092	-0.096	-0.101
	(0.080)	(0.081)	(0.055)	(0.063)
	t = -0.633	t = -1.137	t = -1.746	t = -1.608
	p = 0.527	p = 0.256	p = 0.081	p = 0.108
Discuss:FCers:SubjectPartyDem	-0.050	-0.079	-0.063	-0.057
	(0.067)	(0.072)	(0.055)	(0.079)
	t = -0.744	t = -1.094	t = -1.147	t = -0.722
	p = 0.457	p = 0.275	p = 0.252	p = 0.471
Discuss:Journ:SubjectPartyInd	-0.024	-0.054	0.015	0.043
	(0.081)	(0.083)	(0.074)	(0.075)
	t = -0.291	t = -0.644	t = 0.207	t = 0.569
	p = 0.771	p = 0.520	p = 0.837	p = 0.570
Discuss:Journ:SubjectPartyDem	-0.011	-0.030	-0.024	-0.007
	(0.069)	(0.076)	(0.030)	(0.037)
	t = -0.161	t = -0.392	t = -0.799	t = -0.183
	p = 0.873	p = 0.695	p = 0.425	p = 0.855
NatRep:Size30:Qual:Discuss	-0.132	-0.164	0.080	0.043
	(0.220)	(0.223)	(0.071)	(0.073)
	t = -0.600	t = -0.736	t = 1.138	t = 0.588
	p = 0.549	p = 0.462	p = 0.256	p = 0.557
NatRep:Size3000:Qual:Discuss	-0.229	-0.210	-0.047	-0.093
	(0.233)	(0.249)	(0.099)	(0.096)
	t = -0.980	t = -0.843	t = -0.480	t = -0.962
	p = 0.328	p = 0.400	p = 0.632	p = 0.337

SMUsers:Size30:Qual:Discuss	0.038	-0.108	0.010	0.016
	(0.233)	(0.251)	(0.082)	(0.096)
	t = 0.161	t = -0.431	t = 0.125	t = 0.161
	p = 0.872	p = 0.667	p = 0.901	p = 0.873
SMUsers:Size3000:Qual:Discuss	0.116	0.068	0.181*	0.199*
	(0.229)	(0.245)	(0.085)	(0.091)
	t = 0.506	t = 0.278	t = 2.134	t = 2.191
	p = 0.613	p = 0.781	p = 0.033	p = 0.029
PolBal:Size30:Qual:Discuss	-0.188	-0.231	0.091	0.047
	(0.208)	(0.220)	(0.118)	(0.124)
	t = -0.906	t = -1.048	t = 0.772	t = 0.382
	p = 0.365	p = 0.295	p = 0.441	p = 0.703
PolBal:Size3000:Qual:Discuss	-0.065	-0.124	0.111	0.028
	(0.241)	(0.247)	(0.075)	(0.089)
	t = -0.269	t = -0.500	t = 1.488	t = 0.317
	p = 0.789	p = 0.618	p = 0.137	p = 0.752
NatRep:Size30:Qual:SubjectPartyInd	-0.128	-0.205	-0.156	-0.234
	(0.262)	(0.255)	(0.098)	(0.123)
	t = -0.487	t = -0.802	t = -1.588	t = -1.899
	p = 0.626	p = 0.423	p = 0.113	p = 0.058
NatRep:Size3000:Qual:SubjectPartyInd	-0.226	-0.188	-0.038	-0.069
	(0.266)	(0.269)	(0.103)	(0.124)
	t = -0.850	t = -0.698	t = -0.364	t = -0.553
	p = 0.396	p = 0.486	p = 0.716	p = 0.581
NatRep:Size30:Qual:SubjectPartyDem	0.150	0.109	0.105	0.061
	(0.209)	(0.220)	(0.098)	(0.112)

	t = 0.715	t = 0.494	t = 1.070	t = 0.547
	p = 0.475	p = 0.622	p = 0.285	p = 0.585
NatRep:Size3000:Qual:SubjectPartyDem	0.086	0.109	0.033	0.008
	(0.212)	(0.226)	(0.088)	(0.105)
	t = 0.408	t = 0.484	t = 0.372	t = 0.072
	p = 0.684	p = 0.629	p = 0.711	p = 0.943
SMUsers:Size30:Qual:SubjectPartyInd	-0.492*	-0.648**	0.005	0.024
	(0.249)	(0.251)	(0.164)	(0.172)
	t = -1.979	t = -2.581	t = 0.032	t = 0.141
	p = 0.048	p = 0.010	p = 0.975	p = 0.888
SMUsers:Size3000:Qual:SubjectPartyInd	-0.463	-0.501	-0.011	0.071
	(0.261)	(0.272)	(0.126)	(0.116)
	t = -1.771	t = -1.845	t = -0.085	t = 0.612
	p = 0.077	p = 0.066	p = 0.933	p = 0.541
SMUsers:Size30:Qual:SubjectPartyDem	-0.166	-0.105	0.071	0.072
	(0.196)	(0.208)	(0.086)	(0.101)
	t = -0.848	t = -0.507	t = 0.831	t = 0.707
	p = 0.397	p = 0.612	p = 0.407	p = 0.480
SMUsers:Size3000:Qual:SubjectPartyDem	0.005	0.004	0.091	0.091
	(0.205)	(0.225)	(0.093)	(0.096)
	t = 0.026	t = 0.019	t = 0.984	t = 0.948
	p = 0.980	p = 0.985	p = 0.325	p = 0.344
PolBal:Size30:Qual:SubjectPartyInd	0.067	0.030	0.118	0.108
	(0.243)	(0.256)	(0.143)	(0.141)
	t = 0.275	t = 0.117	t = 0.822	t = 0.769
	p = 0.784	p = 0.908	p = 0.411	p = 0.442

PolBal:Size3000:Qual:SubjectPartyInd	0.056	0.015	0.096	0.048
	(0.264)	(0.263)	(0.092)	(0.100)
	t = 0.214	t = 0.057	t = 1.043	t = 0.484
	p = 0.831	p = 0.955	p = 0.297	p = 0.629
PolBal:Size30:Qual:SubjectPartyDem	-0.175	-0.292	0.111	0.068
	(0.199)	(0.204)	(0.092)	(0.068)
	t = -0.879	t = -1.435	t = 1.195	t = 0.999
	p = 0.380	p = 0.152	p = 0.233	p = 0.318
PolBal:Size3000:Qual:SubjectPartyDem	0.108	0.030	0.121	0.029
	(0.215)	(0.219)	(0.072)	(0.078)
	t = 0.504	t = 0.135	t = 1.668	t = 0.374
	p = 0.615	p = 0.893	p = 0.096	p = 0.709
NatRep:Size30:Discuss:SubjectPartyInd	-0.180	-0.208	-0.051	-0.131
	(0.254)	(0.257)	(0.123)	(0.114)
	t = -0.708	t = -0.808	t = -0.412	t = -1.150
	p = 0.479	p = 0.419	p = 0.681	p = 0.251
NatRep:Size3000:Discuss:SubjectPartyInd	-0.007	0.124	-0.012	-0.028
	(0.249)	(0.255)	(0.095)	(0.104)
	t = -0.030	t = 0.485	t = -0.127	t = -0.265
	p = 0.977	p = 0.628	p = 0.900	p = 0.792
NatRep:Size30:Discuss:SubjectPartyDem	-0.239	-0.328	0.132	0.099
	(0.208)	(0.217)	(0.092)	(0.104)
	t = -1.147	t = -1.512	t = 1.440	t = 0.955
	p = 0.252	p = 0.131	p = 0.150	p = 0.340
NatRep:Size3000:Discuss:SubjectPartyDem	-0.232	-0.167	-0.095	-0.129

	(0.204)	(0.219)	(0.094)	(0.103)
	t = -1.137	t = -0.762	t = -1.015	t = -1.246
	p = 0.256	p = 0.447	p = 0.311	p = 0.213
SMUsers:Size30:Discuss:SubjectPartyInd	-0.396	-0.801**	-0.134	-0.142
	(0.276)	(0.282)	(0.128)	(0.134)
	t = -1.435	t = -2.838	t = -1.047	t = -1.060
	p = 0.152	p = 0.005	p = 0.296	p = 0.290
SMUsers:Size3000:Discuss:SubjectPartyInd	-0.425	-0.582*	-0.056	-0.076
	(0.247)	(0.268)	(0.162)	(0.160)
	t = -1.720	t = -2.169	t = -0.345	t = -0.477
	p = 0.086	p = 0.031	p = 0.730	p = 0.634
SMUsers:Size30:Discuss:SubjectPartyDem	-0.213	-0.314	0.018	-0.0004
	(0.211)	(0.219)	(0.079)	(0.097)
	t = -1.007	t = -1.435	t = 0.226	t = -0.004
	p = 0.314	p = 0.152	p = 0.822	p = 0.997
SMUsers:Size3000:Discuss:SubjectPartyDem	0.099	0.051	0.067	0.042
	(0.204)	(0.220)	(0.061)	(0.064)
	t = 0.484	t = 0.232	t = 1.099	t = 0.651
	p = 0.629	p = 0.817	p = 0.272	p = 0.516
PolBal:Size30:Discuss:SubjectPartyInd	0.015	0.025	0.013	-0.005
	(0.269)	(0.269)	(0.157)	(0.152)
	t = 0.056	t = 0.093	t = 0.081	t = -0.032
	p = 0.956	p = 0.926	p = 0.936	p = 0.975
PolBal:Size3000:Discuss:SubjectPartyInd	0.251	0.213	0.223	0.194
	(0.256)	(0.262)	(0.132)	(0.125)

	t = 0.981	t = 0.814	t = 1.686	t = 1.557
	p = 0.327	p = 0.416	p = 0.092	p = 0.120
PolBal:Size30:Discuss:SubjectPartyDem	0.179	0.100	0.111	0.104
	(0.207)	(0.216)	(0.073)	(0.086)
	t = 0.866	t = 0.463	t = 1.535	t = 1.206
	p = 0.387	p = 0.644	p = 0.125	p = 0.228
PolBal:Size3000:Discuss:SubjectPartyDem	0.519*	0.445*	0.258***	0.214*
	(0.215)	(0.222)	(0.078)	(0.084)
	t = 2.415	t = 2.010	t = 3.317	t = 2.556
	p = 0.016	p = 0.045	p = 0.001	p = 0.011
NatRep:Qual:Discuss:SubjectPartyInd	-0.164	-0.099	0.146	0.152
	(0.259)	(0.272)	(0.114)	(0.098)
	t = -0.633	t = -0.363	t = 1.283	t = 1.558
	p = 0.527	p = 0.717	p = 0.200	p = 0.120
NatRep:Qual:Discuss:SubjectPartyDem	-0.352	-0.293	0.078	0.074
	(0.199)	(0.220)	(0.051)	(0.067)
	t = -1.764	t = -1.330	t = 1.523	t = 1.103
	p = 0.078	p = 0.184	p = 0.128	p = 0.270
SMUsers:Qual:Discuss:SubjectPartyInd	-0.563	-0.716*	0.037	0.032
	(0.288)	(0.309)	(0.120)	(0.133)
	t = -1.959	t = -2.319	t = 0.310	t = 0.240
	p = 0.051	p = 0.021	p = 0.757	p = 0.811
SMUsers:Qual:Discuss:SubjectPartyDem	-0.008	-0.007	-0.045	-0.038
	(0.225)	(0.243)	(0.089)	(0.096)
	t = -0.036	t = -0.029	t = -0.509	t = -0.398
	p = 0.972	p = 0.978	p = 0.611	p = 0.691

PolBal:Qual:Discuss:SubjectPartyInd	-0.499*	-0.474	0.093	0.016
	(0.248)	(0.252)	(0.136)	(0.144)
	t = -2.016	t = -1.881	t = 0.681	t = 0.111
	p = 0.044	p = 0.060	p = 0.497	p = 0.912
PolBal:Qual:Discuss:SubjectPartyDem	-0.014	-0.014	0.159*	0.120
	(0.210)	(0.217)	(0.070)	(0.111)
	t = -0.066	t = -0.065	t = 2.257	t = 1.080
	p = 0.948	p = 0.949	p = 0.024	p = 0.281
NatRep:Size30:Qual:Discuss:SubjectPartyInd	0.030	0.094	0.035	0.127
	(0.371)	(0.386)	(0.159)	(0.146)
	t = 0.081	t = 0.244	t = 0.223	t = 0.872
	p = 0.936	p = 0.807	p = 0.824	p = 0.384
NatRep:Size3000:Qual:Discuss:SubjectPartyInd	0.390	0.235	0.034	0.002
	(0.367)	(0.375)	(0.141)	(0.145)
	t = 1.063	t = 0.627	t = 0.241	t = 0.015
	p = 0.288	p = 0.531	p = 0.810	p = 0.988
NatRep:Size30:Qual:Discuss:SubjectPartyDem	0.404	0.449	-0.219	-0.198
	(0.287)	(0.304)	(0.132)	(0.147)
	t = 1.407	t = 1.478	t = -1.657	t = -1.346
	p = 0.160	p = 0.140	p = 0.098	p = 0.179
NatRep:Size3000:Qual:Discuss:SubjectPartyDem	0.267	0.202	-0.004	0.009
	(0.303)	(0.328)	(0.091)	(0.115)
	t = 0.881	t = 0.615	t = -0.047	t = 0.075

	p = 0.379	p = 0.539	p = 0.963	p = 0.941
SMUsers:Size30:Qual:Discuss:SubjectPartyInd	0.569	0.947*	0.101	0.117
	(0.383)	(0.407)	(0.192)	(0.228)
	t = 1.486	t = 2.329	t = 0.527	t = 0.512
	p = 0.138	p = 0.020	p = 0.598	p = 0.609
SMUsers:Size3000:Qual:Discuss:SubjectPartyInd	0.500	0.720	-0.132	-0.173
	(0.375)	(0.405)	(0.198)	(0.171)
	t = 1.331	t = 1.775	t = -0.667	t = -1.013
	p = 0.184	p = 0.076	p = 0.505	p = 0.312
SMUsers:Size30:Qual:Discuss:SubjectPartyDem	-0.072	0.020	0.040	0.037
	(0.302)	(0.320)	(0.130)	(0.145)
	t = -0.237	t = 0.063	t = 0.308	t = 0.253
	p = 0.813	p = 0.951	p = 0.759	p = 0.801
SMUsers:Size3000:Qual:Discuss:SubjectPartyDem	-0.193	-0.165	-0.115	-0.142
	(0.298)	(0.316)	(0.141)	(0.162)
	t = -0.646	t = -0.522	t = -0.814	t = -0.880
	p = 0.519	p = 0.602	p = 0.416	p = 0.379
PolBal:Size30:Qual:Discuss:SubjectPartyInd	0.340	0.302	-0.179	-0.109
	(0.334)	(0.354)	(0.235)	(0.252)
	t = 1.018	t = 0.852	t = -0.760	t = -0.433
	p = 0.309	p = 0.395	p = 0.448	p = 0.666
PolBal:Size3000:Qual:Discuss:SubjectPartyInd	0.297	0.357	-0.334	-0.225

	(0.373)	(0.379)	(0.179)	(0.190)
	t = 0.797	t = 0.942	t = -1.865	t = -1.184
	p = 0.426	p = 0.347	p = 0.063	p = 0.237
PolBal:Size30:Qual:Discuss:SubjectPartyDem	0.133	0.249	-0.256	-0.196
	(0.275)	(0.286)	(0.148)	(0.179)
	t = 0.485	t = 0.870	t = -1.730	t = -1.093
	p = 0.628	p = 0.385	p = 0.084	p = 0.275
PolBal:Size3000:Qual:Discuss:SubjectPartyDem	-0.291	-0.131	-0.288**	-0.173
	(0.310)	(0.323)	(0.102)	(0.134)
	t = -0.942	t = -0.405	t = -2.835	t = -1.289
	p = 0.347	p = 0.686	p = 0.005	p = 0.198
Constant	3.775***	3.750***	0.580***	0.588***
	(0.057)	(0.059)	(0.018)	(0.021)
	t = 66.487	t = 63.621	t = 32.858	t = 28.352
	p = 0.000	p = 0.000	p = 0.000	p = 0.000

Note: *p<.05, **p<.01, ***p<.001

OLS predicting legitimacy or choice of juries by multiple jury categories, with partisanship moderator. Baseline levels are domain expert jury, size of 3, no qualifications or discussion; and Republican party.

8. Secondary Analyses – Measurement Error Corrections.

Here we conduct robustness analyses on our primary binary choice outcome models, as recommended by (2). Following their recommendations, we include a repeat of the first jury pair as a final jury pair decision, with the order of the two jury columns switched, in order to estimate intra-respondent reliability (IRR). In line with prior conjoint experiment research, we observe an IRR of 0.752 (τ=0.145). We then use the alternative estimators specified in equation 11 of (2) to calculate adjusted means and coefficients for our category-level and jury-specific choice models, and use bootstrapping (1k iterations of sampling respondents with replacement and calculating alternative estimates) to estimate standard errors.

Table S9. *Jury choice predicted by category-level jury types and features, with IRR measurement error correction. Holdout jury is experts (no discussion).*

	Est_corrected	SEs_corrected	CI_Lower	CI_Upper
NonJury	-0.481	0.02	-0.521	-0.441
Layperson	-0.304	0.027	-0.357	-0.251
Discuss	0.075	0.018	0.04	0.109
Layperson:Size30	0.175	0.031	0.115	0.235
Layperson:Size3000	0.222	0.032	0.159	0.286
Layperson:Qual	0.181	0.031	0.12	0.242
Layperson:Discuss	0	0.034	-0.067	0.067
Layperson:Size30:Qual	0.034	0.044	-0.052	0.121
Layperson:Size3000:Qual	0.025	0.043	-0.06	0.109
Layperson:Size30:Discuss	-0.001	0.045	-0.089	0.088
Layperson:Size3000:Discuss	-0.03	0.043	-0.114	0.055
Layperson:Qual:Discuss	-0.004	0.045	-0.092	0.085
Layperson:Size30:Qual:Discuss	-0.011	0.064	-0.137	0.115
Layperson:Size3000:Qual:Discuss	-0.02	0.062	-0.142	0.102

Table S10. *Jury choice predicted by all juries and features, with IRR measurement error correction. Holdout jury is domain experts (no discussion).*

	Est_corrected	SEs_corrected	CI_Lower	CI_Upper
Coin	-0.584	0.031	-0.646	-0.523
Algo	-0.52	0.033	-0.585	-0.454
Zuck	-0.587	0.031	-0.648	-0.527
NatRep	-0.364	0.044	-0.45	-0.279
SMUsers	-0.461	0.042	-0.543	-0.378
PolBal	-0.338	0.045	-0.425	-0.251
FCers	-0.029	0.029	-0.087	0.028
Journ	-0.22	0.031	-0.28	-0.159
Discuss	0.081	0.029	0.023	0.138
NatRep:Size30	0.183	0.054	0.077	0.289
NatRep:Size3000	0.268	0.053	0.163	0.372
SMUsers:Size30	0.174	0.05	0.076	0.272
SMUsers:Size3000	0.229	0.051	0.128	0.33
PolBal:Size30	0.17	0.052	0.068	0.272
PolBal:Size3000	0.17	0.052	0.069	0.271
NatRep:Qual	0.182	0.051	0.082	0.282

SMUsers:Qual	0.174	0.05	0.076	0.273
PolBal:Qual	0.187	0.054	0.081	0.293
NatRep:Discuss	-0.019	0.062	-0.142	0.103
SMUsers:Discuss	0.037	0.059	-0.079	0.153
PolBal:Discuss	-0.034	0.059	-0.149	0.082
Discuss:FCers	0.007	0.039	-0.069	0.082
Discuss:Journ	-0.024	0.041	-0.103	0.056
NatRep:Size30:Qual	0.035	0.072	-0.106	0.175
NatRep:Size3000:Qual	-0.005	0.072	-0.146	0.136
SMUsers:Size30:Qual	0.054	0.074	-0.091	0.198
SMUsers:Size3000:Qual	0.039	0.072	-0.102	0.18
PolBal:Size30:Qual	0.014	0.075	-0.133	0.161
PolBal:Size3000:Qual	0.04	0.074	-0.104	0.185
NatRep:Size30:Discuss	0.013	0.075	-0.134	0.159
NatRep:Size3000:Discuss	-0.076	0.074	-0.222	0.07
SMUsers:Size30:Discuss	-0.046	0.073	-0.19	0.097
SMUsers:Size3000:Discuss	-0.1	0.075	-0.247	0.046
PolBal:Size30:Discuss	0.032	0.073	-0.111	0.175
PolBal:Size3000:Discuss	0.089	0.072	-0.052	0.231
NatRep:Qual:Discuss	0.051	0.073	-0.092	0.194
SMUsers:Qual:Discuss	-0.058	0.075	-0.205	0.089
PolBal:Qual:Discuss	-0.003	0.074	-0.149	0.142
NatRep:Size30:Qual:Discuss	-0.058	0.102	-0.258	0.141
NatRep:Size3000:Qual:Discuss	-0.029	0.102	-0.229	0.172
SMUsers:Size30:Qual:Discuss	0.041	0.109	-0.173	0.255
SMUsers:Size3000:Qual:Discuss	0.075	0.103	-0.128	0.277
PolBal:Size30:Qual:Discuss	-0.014	0.107	-0.223	0.195
PolBal:Size3000:Qual:Discuss	-0.11	0.106	-0.318	0.097

9. References.

1. A. J. Berinsky, M. F. Margolis, M. W. Sances, Can we turn shirkers into workers? *J. Exp. Soc. Psychol.* **66**, 20–28 (2016).

2. K. Clayton, *et al.*, Correcting Measurement Error Bias in Conjoint Survey Experiments. *Am. J. Polit. Sci.* **12**, 1–11 (2023).
